# Multi‐Dimensional Synergistic Engineering for Boosting Nanozyme Catalysis

**DOI:** 10.1002/advs.202508150

**Published:** 2025-08-29

**Authors:** Yuechun Li, Zhaowen Cui, Chenxin Ji, Cancan Jia, Wentao Zhang, Jianlong Wang, Yizhong Shen

**Affiliations:** ^1^ College of Food Science and Engineering Northwest A&F University 22 Xinong Road Yangling Shaanxi 712100 China; ^2^ School of Food and Biological Engineering Hefei University of Technology 193 Tunxi Road Hefei Anhui 230009 China

**Keywords:** boosting nanozyme catalysis, electronic structure, external regulation, machine learning, morphological structure

## Abstract

Pursuing nanozymes with predominant catalytic activities holds great potential in meeting the needs of multi‐field applications, such as in the environment, yet remains challenging. Herein, the cutting‐edge strategies for boosting nanozyme catalysis are systematically analyzed from four synergistic dimensions, and a theoretical framework is developed integrating morphological structure, electronic structure, external stimulation, and machine learning (ML)‐aided design. From the morphological perspective, the structure‐activity relationship between nanostructures and catalytic performance is elucidated, revealing the regulatory mechanism underlying active site exposure, substrate accessibility, and electron transfer kinetics. Subsequently, the electronic structure governed by catalytic activities through precisely optimizing adsorption energy and reaction pathways, such as *d*‐band center, *e_g_* occupancy, defect engineering, spin state, etc., is discussed in depth. Then, the mechanism underlying the dynamic regulation of catalytic activity via external stimulations, such as ultrasound, light, electric field, etc., is systematically summarized. Notably, the revolutionary role of ML‐driven high‐throughput screening in analyzing complex structure‐activity relationships and accelerating the discovery of high‐performance nanozymes is emphasized. Ultimately, this paper highlights the key role of interdisciplinary integration, encompassing material science, catalytic engineering, and artificial intelligence, etc., in overcoming current bottlenecks, unlocking the potential of nanozymes to address global challenges, and providing a new perspective for advancing the development of nanozymology.

## Graphical abstract

This review systematically analyzes cutting‐edge strategies for boosting nanozyme catalysis through multi‐dimensional synergistic engineering, containing morphology, electronic structure modulation, external stimulation, and machine learning‐aided design. A theoretical framework is proposed to decode structure‐activity relationships, offering interdisciplinary insights to improve the nanozymes' catalysis in addressing global challenges, thus advancing nanozymology.

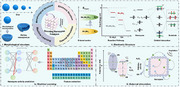

## 1 Introduction

**Figure 1:**
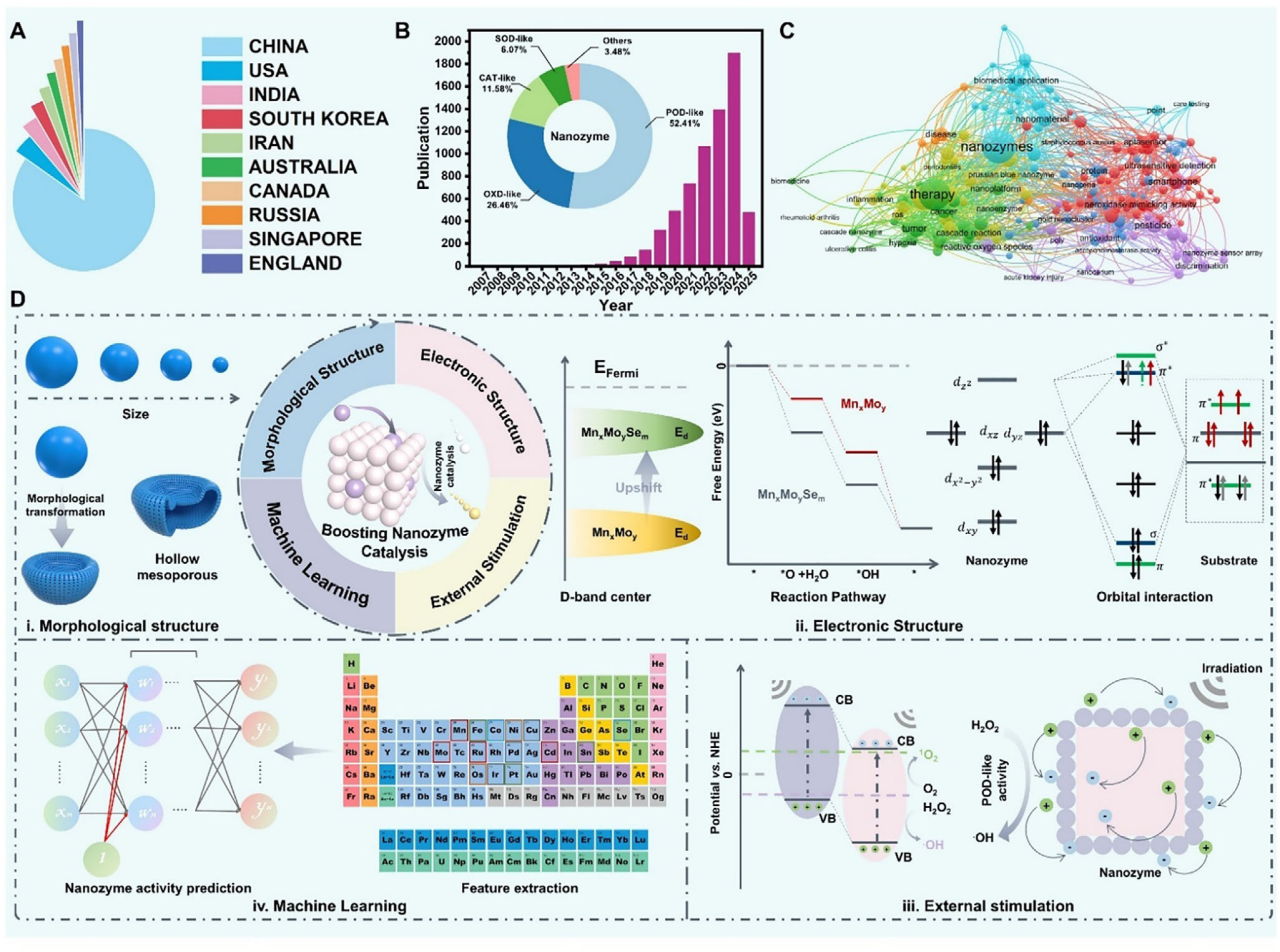
A) Top ten countries publishing papers on nanozyme research. B) Publication trends of nanozyme‐related papers and classification analysis in nanozyme development. C) Distribution of research fields in nanozymology. D) Multi‐dimensional synergistic engineering for boosting nanozyme catalysis, encompassing morphological structure, electronic structure, external stimulation, and ML.

Nanozymes, engineered nanomaterials with enzyme‐mimicking activities, have emerged as a transformative class of catalytic systems over the past two decades,^[^ ^1^, ^2^ ^]^ and have been widely applied in diverse fields, such as environmental monitoring and control.^[^ ^3^ ^]^ By integrating the tunable physicochemical properties of nanomaterials with the catalytic specificity of natural enzymes, nanozymes exhibit unprecedented advantages in terms of stability, scalability, and multifunctionality.^[^ ^4^, ^5^ ^]^ To date, over 60 countries and regions have contributed to more than 6600 publications concerning nanozyme research (**Figure** **1**A), covering peroxidase (POD)‐like, oxidase (OXD)‐like, catalase (CAT)‐like, superoxide dismutase (SOD)‐like, and other activities (Figure 1B). Their applications span a diverse range of fields, including biosensing, therapeutics, environmental remediation, and industrial catalysis (Figure 1C).^[^ ^6^, ^7^, ^8^ ^]^ However, despite substantial advancements, the catalytic efficiency of nanozymes still often lags behind that of their natural counterparts, a limitation stemming from fundamental challenges in controlling active site architectures, modulating electron transfer kinetics, and engineering adaptive responses to dynamic reaction environments.^[^ ^9^, ^10^ ^]^ Addressing the limitation in catalytic activity demands a paradigm shift: moving from empirical material discovery toward rational design strategies rooted in atomic‐level insights into structure‐activity relationships.^[^ ^11^, ^12^ ^]^ Recent pivotal breakthroughs in nanotechnology, computational modeling, and advanced characterization techniques have ushered in a new era of precision engineering for nanozyme development.^[^ ^13^, ^14^ ^]^ Advances in morphological control facilitate the fabrication of hierarchical nanostructures with maximized active surface areas and optimized mass transport pathways.^[^ ^15^, ^16^ ^]^ Concurrently, innovations in electronic structure manipulation, encompassing defect engineering, heteroatom doping, and interface engineering, offer atomic‐level levers to modulate adsorption energetics and reaction coordinates.^[^ ^17^, ^18^ ^]^ Furthermore, the integration of stimuli‐responsive functionalities enables dynamic modulation of catalytic behavior, thereby mimicking the allosteric regulation of natural enzymes.^[^ ^19^ ^]^ Additionally, to achieve the broad range of optimal parameters for nanozymes, including composition, size, morphology, surface chemistry, and defects, conventional strategies are often hampered by the formidable challenge of empirical, “trial‐and‐error” material discovery. This is precisely where machine learning (ML) has begun to play a pivotal role, emerging as a powerful accelerant, decoding complex correlations between material descriptors and catalytic outcomes and predicting novel nanozyme candidates beyond the scope of conventional design.^[^ ^20^ ^]^

This review presents a systematic examination of state‐of‐the‐art strategies for boosting nanozyme catalysis across four synergistic dimensions, and outlines a comprehensive framework integrating morphological structure, electronic structure optimization, external stimulus responsiveness engineering, and ML‐assisted design (Figure 1D). The catalytic activity of nanozyme, encompassing Michaelis–Menten constants (*K*_m_), maximum reaction velocity (*V*_max_), catalytic rate constant (*K*_cat_), and specific activity (SA), is tabulated in **Table** **1** to facilitate comparative analysis of their catalytic performance. By elucidating the interplay between structural features, including size, morphology, hollow, and pore, and catalytic performance, we critically analyze how nanoscale architecture governs active site exposure, substrate accessibility, and electron transfer dynamics. Additionally, we delve into the tuning of electronic properties, encompassing *d*‐band center, *e_g_* occupancy, defect engineering, and spin‐state modulation, and critically analyze how these strategies enable precise control over adsorption energies and reaction pathways. Furthermore, we explore the burgeoning role of external stimuli, including ultrasound (US), light, electric fields, etc., in dynamically boosting nanozyme catalytic activity. Finally, we showcase how ML‐driven approaches are revolutionizing the discovery of high‐performance nanozymes by deciphering complex structure‐activity relationships and accelerating materials discovery and design. Collectively, these strategies not only deepen fundamental understanding of nanozyme catalysis but also pave the way for next‐generation nanozymes custom‐engineered for real‐world applications. This review closes with perspectives on future challenges and opportunities, highlighting the transformative potential of interdisciplinary approaches that integrate materials science, catalysis, and artificial intelligence to unleash the full potential of nanozymes for tackling global challenges.

**Table 1:** Summary of nanozyme catalytic activities in terms of K_m_, V_max_, K_cat_, and SA.

Nanozyme	Enzyme‐like activity	K_m_ (mM)	V_max_ (10^−8^ M s^−1^)	K_cat_ (10^4^ s^−1^)	Specific activity (U mg^−1^)	Refs.
Fe_3_O_4_NPs	POD‐like	H_2_O_2_: 154 TMB: 0.098	H_2_O_2_: 9.78 TMB: 3.44	H_2_O_2_: 8.58 TMB: 3.02	/	[29]
RuRhPdPtIr	POD‐like	H_2_O_2_: 4094 TMB: 0.0344	H_2_O_2_: 18.82 TMB: 24.57	/	/	[31]
Ca_2_Mn_8_O_16_NSs	GSH POD‐like	GSH: 0.76	GSH:121.4	/	/	[37]
Mn_3_O_4_/CeO_x_	CAT‐like	H_2_O_2_: 7.212	H_2_O_2_: 588.2	/	/	[32]
Mn_3_O_4_/CeO_x_	SOD‐like	/	/	/	19 176.05	[32]
Co‐based HNCs	OXD‐like	TMB: 0.375	2.56	/	/	[48]
Cu_1.5_Mn_1.5_O_4_	POD‐like	H_2_O_2_: 1.453	H_2_O_2_: 714.091	/	/	[49]
MHNs	POD‐like	H_2_O_2_: 4.904 TMB: 0.076	H_2_O_2_: 12.02 TMB: 1.96	/	/	[52]
Fe‐Co‐Mn SAzymes	CAT‐like OXD‐like	TMB: 0.86	TMB: 304.8	/	/	[33]
FeNPs@Co_3_O_4_ HNCs	POD‐like	H_2_O_2_: 0.019 TMB: 0.488	H_2_O_2_: 1.7 TMB: 20.6	/	/	[54]
PHMZCO‐AT	POD‐like	H_2_O_2_: 21.01	H_2_O_2_: 7.81	/	1.57	[34]
PHMZCO‐AT	CAT‐like	H_2_O_2_: 109.78	H_2_O_2_: 0.22 (mg L^−1^ mL^−1^)	/	/	[34]
PCN‐222@Cyt c	POD‐like	H_2_O_2_: 3.7	/	/	/	[59]
PtZnCd	POD‐like	H_2_O_2_: 0.092	H_2_O_2_: 12.15	0.16	231.589	[71]
PtZnCd	SOD‐like	/	/	/	108.70	[71]
Zn‐SAs@BNC_1000_	POD‐like	H_2_O_2_: 0.10	H_2_O_2_: 430	H_2_O_2_: 0.000269	31.7	[72]
HEzymes	POD‐like	H_2_O_2_: 0.6 TMB: 0.07	H_2_O_2_: 16.62 TMB: 6.26	H_2_O_2_: 538.3 TMB: 203.3	109.65	[73]
Fe_1_Mn_1_–NC_e_	POD‐like	H_2_O_2_: 0.39	/	H_2_O_2_: 0.0156	/	[74]
LaNiO_3‐_ *_δ_*	POD‐like	H_2_O_2_: 359.92	H_2_O_2_: 463	/	/	[82]
La_0.5_Sr_0.5_FeO_3‐δ_	POD‐like	H_2_O_2_: 8.14	H_2_O_2_: 13	/	/	[82]
LiCo_2_O_4_	POD‐like	H_2_O_2_: 265.84	H_2_O_2_: 128.63	/	/	[78]
H‐MoN_5_@PtN_4_/C	POD‐like	TMB: 0.024	TMB: 4.09	/	34.33	[84]
FeNC‐Pd_NC_	POD‐like	H_2_O_2_: 9.58 TMB: 1.97	H_2_O_2_: 1093 TMB: 1430	H_2_O_2_: 0.00051 TMB: 0.000668	95.68	[85]
Oligomeric nanozyme	GSH POD‐like	H_2_O_2_: 0.8204 GSH: 0.8688	H_2_O_2_: 121.62 GSH: 30.82	/	/	[86]
FeCDs	POD‐like	H_2_O_2_: 0.03	H_2_O_2_: 20	/	/	[87]
A‐Pd@MoO_3–_ *_x_* NH	OXD‐like	TMB: 0.054	TMB: 3.21	TMB: 0.0000057	/	[88]
Ru@PSS	POD‐like	H_2_O_2_: 0.31 TMB: 0.615	/	H_2_O_2_: 0.000004 TMB: 0.158	2820	[89]
Fe–CeO_v_	POD‐like	H_2_O_2_: 20.15	H_2_O_2_: 8.24	/	/	[94]
CP600−6	POD‐like	H_2_O_2_: 9.559 TMB: 0.253	H_2_O_2_: 130.89 TMB: 127.55	/	/	[97]
CP10000−6	POD‐like	H_2_O_2_: 9.559 TMB: 0.253	H_2_O_2_: 9.559 TMB: 0.253	/	/	[97]
MnMoO_x_	CAT‐like	H_2_O_2_: 30.052	H_2_O_2_: 1634.2	/	/	[99]
MnMoO_x_	OXD‐like	TMB: 0.376	TMB: 23.3	/	/	[99]
Co_4_N/C	LOX‐like	Lactate: 9.08	Lactate: 1330	Lactate: 532	/	[104]
Co_4_N/C	CAT‐like	H_2_O_2_: 173	H_2_O_2_: 530	H_2_O_2_: 212	/	[104]
Co_4_N/C	POD‐like	H_2_O_2_: 70.2	H_2_O_2_: 10.9	H_2_O_2_: 4.36	/	[104]
Co_4_N/C	OXD‐like	TMB: 0.0547	TMB: 1.91	TMB: 0.764	/	[104]
FePc@2D‐Cu–N–C	OXD‐like	TMB: 0.604	TMB: 89.13	TMB: 0.000243	/	[106]
Ir–N_5_	OXD‐like	TMB: 0.97	TMB: 6.14	/	/	[108]
Ir–N_5_	POD‐like	H_2_O_2_: 2.43	H_2_O_2_: 9.23	/	/	[108]
PEG@P@Ce–N/S–C	OXD‐like	TMB: 0.11	TMB: 197.8	TMB: 0.0000699	64.11	[109]
UIO‐66‐Au NPs	POD‐like	H_2_O_2_: 8.13 TMB: 0.0353	H_2_O_2_: 4.16 TMB: 11.2	/	/	[19]
UIO‐66‐Au NPs with US	POD‐like	H_2_O_2_: 5.91 TMB: 0.0222	H_2_O_2_: 7.964 TMB: 17.4	/	/	[19]
BTO/MoS_2_@CA	POD‐like	H_2_O_2_: 1.06	H_2_O_2_: 11.01	/	/	[119]
BTO/MoS_2_@CA with US	POD‐like	H_2_O_2_: 0.57	H_2_O_2_: 14.28	/	/	[119]
ZrO_2−_ *_x_* @Pt/AIPH	CAT‐like	H_2_O_2_: 14.756	H_2_O_2_: 19	/	/	[123]
Pt	CAT‐like	H_2_O_2_: 40.1	H_2_O_2_: 593.17	/	/	[124]
Pt	POD‐like	H_2_O_2_: 0.0163	H_2_O_2_: 9.44	/	/	[124]
LAC‐ZIF‐8	LAC	BPA: 0.15	/	/	/	[125]
LAC_US_‐ZIF‐8	LAC	BPA: 0.10	/	/	/	[125]
CAT‐ZIF‐8	/	/	/	H_2_O_2_: 2881	/	[125]
CAT_US_‐ZIF‐8	/	/	/	H_2_O_2_: 5408	/	[125]
Cyt C‐ZIF‐8	/	H_2_O_2_: 71.91	H_2_O_2_: 3.5×10^6^	H_2_O_2_: 10990	/	[125]
Cyt C_US_‐ZIF‐8	/	H_2_O_2_: 29.00	H_2_O_2_: 3×10^6^	H_2_O_2_: 41282	/	[125]
GOx‐ZIF‐8	/	Glucose: 12.16	Glucose: 3.49×10^5^	Glucose: 2595	/	[125]
GOx_US_‐ZIF‐8	/	Glucose: 27.33	Glucose: 6.42×10^5^	Glucose: 5483	/	[125]
CAT‐ZIF‐8‐ HRP	/	H_2_O_2_: 45.30	H_2_O_2_: 1.8×10^6^	H_2_O_2_: 9118	/	[125]
CAT‐ZIF‐8‐ HRP	/	H_2_O_2_: 79.49	H_2_O_2_: 1.4×10^6^	H_2_O_2_: 7219	/	[125]
Au@CeO_2_	POD‐like	H_2_O_2_: 0.007 TMB: 0.061	H_2_O_2_: 0.83 TMB: 0.15	H_2_O_2_: 0.0000049 TMB: 0.0000009	/	[136]
Au@CeO_2_ with light	POD‐like	H_2_O_2_: 0.006 TMB: 0.207	H_2_O_2_: 0.38 TMB: 1.33	H_2_O_2_: 0.0000080 TMB: 0.0000023	/	[136]
Au NBP@Cu_2_O@PVP	POD‐like	H_2_O_2_: 13.95 TMB: 26.62	H_2_O_2_: 16.67 TMB: 38.46	/	/	[137]
CuS@GDY	POD‐like	H_2_O_2_: 3.26 TMB: 0.773	H_2_O_2_: 3.44 TMB: 3.067	/	/	[138]
CuS@GDY with light	POD‐like	H_2_O_2_: 0.89 TMB: 0.37	H_2_O_2_: 7.70 TMB: 2.79	/	/	[138]
Pd‐Au dimers	POD‐like	H_2_O_2_: 169.52 TMB: 0.061	H_2_O_2_: 36.49 TMB: 23.81	/	/	[139]
Pd‐Au dimers	POD‐like	H_2_O_2_: 129.07 TMB: 0.059	H_2_O_2_: 120.62 TMB: 80.45	/	/	[139]
Cu_2_MoS_4_	POD‐like	H_2_O_2_: 25.46 TMB: 1.36	H_2_O_2_: 42.81 TMB: 27.29	/	/	[140]
Cu_2_MoS_4_	OXD‐like	L‐ascorbic acid: 0.012	L‐ascorbic acid:11	/	/	[140]
IrNCs	CAT‐like	H_2_O_2_: 132	/	/	/	[141]
Ru800	POD‐like	H_2_O_2_: 26.4	H_2_O_2_: 13	/	/	[146]
Ru900	POD‐like	H_2_O_2_: 44.9	H_2_O_2_: 101	/	/	[146]
Ru1000	POD‐like	H_2_O_2_: 18.4	H_2_O_2_: 116	/	/	[146]
Ru800	OXD‐like	GSH: 1.2	GSH: 28	/	/	[146]
Ru900	OXD‐like	GSH: 0.5	GSH: 71	/	/	[146]
Ru1000	OXD‐like	GSH: 0.2	GSH: 87	/	/	[146]
PtMnIr	POD‐like	H_2_O_2_: 0.42	H_2_O_2_: 2.37	/	/	[147]
COF‐CNT	POD‐like	TMB: 0.8	TMB: 220	/	/	[143]
COF‐CNT with electric field	POD‐like	TMB: 3.2	TMB: 900	/	/	[143]
hCOF	POD‐like	TMB: 0.829	TMB:	/	/	[148]
hCOF with electric field	POD‐like	TMB: 2.368	TMB: 220	/	/	[148]
AuAgPtMn‐Ag electrodes	CAT‐like	H_2_O_2_: 197.99	H_2_O_2_: 3516.7	/	/	[149]
Iron oxide	POD‐like	TMB: 0.019	TMB: 5.66	TMB:0.128	/	[153]
Iron oxide with magnetic field	POD‐like	TMB: 0.03	TMB: 6.18	TMB: 0.14	/	[153]
Fe_3_O_4_ NR (D2)	POD‐like	H_2_O_2_: 125.71	H_2_O_2_: 3.88	H_2_O_2_: 0.049	/	[156]
PCN@ZF	POD‐like	H_2_O_2_: 14.23	H_2_O_2_: 5.3×10^4^	/	/	[157]
PCN@ZF with magnetic field	POD‐like	H_2_O_2_: 13.98	H_2_O_2_: 2.61×10^5^	/	/	[157]
Fe_3_O_4_	POD‐like	H_2_O_2_: 12.35	H_2_O_2_: 5.8×10^4^	/	/	[157]
Fe_3_O_4_ with magnetic field	POD‐like	H_2_O_2_: 9.70	H_2_O_2_: 2.13×10^5^	/	/	[157]
FeN_4_ SAzymes	POD‐like	H_2_O_2_: 285	H_2_O_2_: 10.33	H_2_O_2_: 0.216	41.71	[160]
FeN_4_ SAzymes with X‐ray	POD‐like	H_2_O_2_: 167	H_2_O_2_: 8.38	H_2_O_2_: 0.31	60.51	[160]
FeN_4_ SAzymes	OXD‐like	GSH: 0.13	GSH: 60.67	GSH: 0.022	1.57	[160]
FeN_4_ SAzymes with X‐ray	OXD‐like	GSH: 0.08	GSH: 78.83	GSH: 0.029	1.18	[160]
P–RuCu	POD‐like	H_2_O_2_: 0.25 TMB: 0.07	H_2_O_2_: 8.17 TMB: 9.91	/	/	[163]
Fe‐SAzymes	POD‐like	H_2_O_2_: 4.73	H_2_O_2_: 40.5	/	/	[167]
Fe‐SAzymes with NIR‐I illumination	POD‐like	H_2_O_2_: 1.49	H_2_O_2_: 71.3	/	/	[167]
ZnSnO_3_@MXene	POD‐like	H_2_O_2_: 58.87	H_2_O_2_: 10.6	/	/	[170]
ZnSnO_3_@MXene with water bath	POD‐like	H_2_O_2_: 74.75	H_2_O_2_: 16.4	/	/	[170]
Bi_2_Fe_4_O_9_ NSs	OXD‐like	GSH: 1.85	GSH: 92	/	/	[173]
Bi_2_Fe_4_O_9_ NSs	CAT‐like	H_2_O_2_: 142.52	/	/	/	[173]
Bi_2_Fe_4_O_9_ NSs	POD‐like	H_2_O_2_: 211.29	H_2_O_2_: 56	/	/	[173]
RuNPs	POD‐like	/	/	TMB: 0.000625	/	[20]
RhNPs	POD‐like	/	/	TMB: 0.000429	/	[20]
PdNPs	POD‐like	/	/	TMB: 0.000128	/	[20]
AgNPs	POD‐like	/	/	TMB: 0.000003	/	[20]
MnIn_2_Se_4_	CAT‐like	/	/	/	169.53	[181]
NiCo_2_Se_4_	CAT‐like	/	/	/	103.56	[181]
SrDy_2_O_4_	CAT‐like	/	/	/	284.32	[181]
CuIn_2_S_4_	CAT‐like	/	/	/	149.86	[181]
MnIn_2_Se_4_	SOD‐like	/	/	/	42.58	[181]
NiCo_2_Se_4_	SOD‐like	/	/	/	31.54	[181]
SrDy_2_O_4_	SOD‐like	/	/	/	86.37	[181]
CuIn_2_S_4_	SOD‐like	/	/	/	36.84	[181]
MnPS_3_	SOD‐like	/	Pyrogallol: 2.76×10^−8^	/	721.12	[182]
B‐GDY	POD‐like	H_2_O_2_: 2.46 TMB: 0.62	H_2_O_2_: 4.61 TMB: 6.47	/	/	[183]
N‐GDY	POD‐like	H_2_O_2_: 2.11 TMB: 0.37	H_2_O_2_: 5.88 TMB: 2.60	/	/	[183]
O‐GDY	POD‐like	H_2_O_2_: 1.58 TMB: 0.22	H_2_O_2_: 0.80 TMB: 1.88	/	/	[183]
Si‐GDY	POD‐like	H_2_O_2_: 1.33 TMB: 2.31	H_2_O_2_: 3.25 TMB: 13.84	/	/	[183]
P‐GDY	POD‐like	H_2_O_2_: 5.65 TMB: 2.27	H_2_O_2_: 3.42 TMB: 9.46	/	/	[183]
S‐GDY	POD‐like	H_2_O_2_: 3.60 TMB: 1.37	H_2_O_2_: 3.25 TMB: 7.08	/	/	[183]

## 2 Morphological Structure for Boosting Nanozyme Catalysis

In the expansive field of nanozyme research, exploring the mechanisms by which morphological features, such as size, overall morphology, hollow architectures, and porous structures, regulate catalytic activities is key to unlocking the full application potential of nanozymes, as these features jointly shape their unique catalytic performance landscape.^[^ ^21^, ^22^ ^]^ Different geometric morphologies, by modulating the arrangement and coordination number of surface atoms, profoundly influence the exposure extent, quantitative distribution, and electron transfer properties of active sites, thereby underpinning the catalytic performance of nanozyme.^[^ ^12^, ^23^ ^]^ Meanwhile, size variations function as a fine‐tuning tool, precisely modulating the specific surface area and the number of active sites in nanozymes, thereby facilitating distinct interaction scenarios between substrate molecules and nanozymes.^[^ ^24^, ^25^ ^]^ Hollow structures, endowed with their unique spatial merits, play pivotal roles across multiple dimensions, such as increasing the specific surface area, facilitating substrate diffusion, and stabilizing metal atoms, thereby establishing an efficient catalytic microenvironment for reactions.^[^ ^26^ ^]^ Pore structures exhibit even greater versatility within the catalytic system: they not only facilitate the distribution of active sites on the inner surface and aid in the efficient diffusion of substrates and products, but also finely regulate the electronic structure and chemical microenvironment of nanozymes, and enhance the selectivity and yield of catalytic reactions through their unique confinement effects.^[^ ^27^ ^]^ Systematic investigations and in‐depth understanding of these factors can pave the way to unravel the mysteries of nanozyme catalysis, offering solid theoretical support and practical guidance for their innovative applications across diverse fields.

### 2.1 Size

The catalytic performance of nanozymes is strongly dependent on their physical dimensions, with particle size standing as a critical determinant of enzymatic activity. This size‐activity relationship originates primarily from fundamental principles of surface physics: most notably through enhancing specific surface area, which significantly expands their total surface area and thereby exposes more active sites.^[^ ^15^, ^16^ ^]^ These abundant active sites offer more opportunities and possibilities for the interaction between nanozymes and substrate molecules, and thus play a pivotal role in facilitating the entire catalytic reaction process, making them a key factor influencing the catalytic activity of nanozymes.

**Figure 2:**
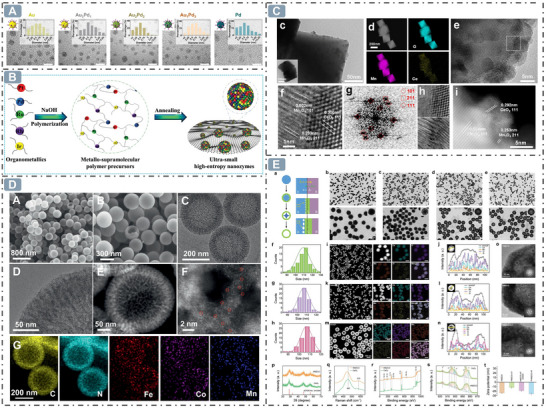
Boosting nanozyme catalysis through morphological engineering. A) The transmission electron microscope (TEM) image of small‐sized nanozymes. Reproduced with permission. ^[^ ^30^ ^]^ *Copyright 2024, The Author(s)*. B) Synthesis of ultra‐small high‐entropy nanozyme. Reproduced with permission.^[^ ^31^ ^]^ *2023 Wiley‐VCH GmbH*. C) TEM image of CeO_x_/Mn_3_O_4_ with lattice distortion. Reproduced with permission.^[^ ^32^ ^]^ *2024 Wiley‐VCH GmbH*. D) Morphology of Fe‐Co‐Mn SAzymes with hollow mesoporous structure. Reproduced with permission.^[^ ^33^ ^]^ *2023 Elsevier B.V. All rights reserved*. E) Morphology of PHMZCO‐AT nanozymes with mesoporous nanostructures. Reproduced with permission.^[^ ^34^ ^]^ *2022 Wiley‐VCH GmbH*.

Reductions in nanozyme size can dramatically enhance the specific surface area and expose more catalytically active sites, thereby increasing contact opportunities with substrate molecules and boosting the catalytic activity of nanozymes.^[^ ^28^ ^]^ For example, Fe_3_O_4_ nanoparticles (NPs) exhibit a notable enhancement in POD‐like activity when downsized from 300 nm to 30 nm, which directly correlates with the increase in surface‐to‐volume ratio. Such dimensional optimization enables more efficient substrate binding by enhancing molecular accessibility to surface atoms.^[^ ^29^ ^]^ The paradigm of “smaller is more active” applies to various nanozyme architectures. AuPd alloy nanozymes with an ultra‐small average size of 2–3 nm exhibit a high specific surface area, which means that the nanozymes per unit mass contain more surface atoms and enhance the utilization of noble metal components, and thereby provide more active sites to interact more effectively with substrate molecules and promote catalytic reaction (**Figure** **2**A).^[^ ^30^ ^]^ Recent breakthroughs with high‐entropy alloy nanozymes (RuRhPdPtIr, 1.5 nm) further confirm that smaller sizes lead to more surface‐exposed atoms, as evidenced by their low *K*_m_ value of 4.094 M and 34.40 µM for H_2_O_2_ and 3,3′,5,5′‐tetramethylbenzidine (TMB), respectively (Figure 2B).^[^ ^31^ ^]^ The size‐activity correlation in nanozymes offers distinct opportunities for bioinspired catalyst design. By precisely regulating NPs size through advanced synthesis techniques (e.g., microemulsion templating, atomic layer deposition), catalytic parameters, including turnover number, substrate affinity, and reaction selectivity, can be systematically tuned, providing a promising avenue for tailoring their catalytic activities. Ongoing exploration and understanding of how nanozyme size influences performance will not only deepen our knowledge of nanozymology, but also lay the groundwork for the rational design and optimization of next‐generation nanozymes with programmable catalytic activities.

### 2.2 Morphology

The catalytic performance of nanozymes is exquisitely regulated by their morphological features, which orchestrate atomic arrangements, coordination environments, and electron transport pathways.^[^ ^12^, ^35^ ^]^ Beyond mere size effects, advanced nanostructural engineering, encompassing 2D nanosheets (NSs), anisotropic nanorods (NRs), Janus architectures, and support‐anchored systems, allows for precise modulation of active site accessibility, density, and electronic states. This morphological tunability facilitates the elucidation of efficient nanozyme catalytic mechanisms and offers a robust toolkit for emulating, and even outperforming, the catalytic sophistication of natural enzymes.

Nanozymes with distinct morphologies exhibit variations in the arrangement and coordination number of surface atoms, directly influencing the exposure extent of active sites.^[^ ^36^ ^]^ For example, due to their large planar area, 2D NSs demonstrate morphology‐driven activity enhancement relative to NPs via maximized atomic exposure, which increases contact opportunities between substrates and active sites, thereby facilitating catalytic reactions. Their larger specific surface area allows 2D Ca_2_Mn_8_O_16_ NSs, endowed with glutathione (GSH) POD‐like activity, to interact more effectively with GSH and H_2_O_2_, accelerating the GSH consumption and H_2_O_2_ transformation, and thereby boosting nanozyme catalytic activity.^[^ ^37^ ^]^ Additionally, the coordination environment of atoms on the side surfaces versus at the end of NRs differs, rendering end atoms generally more active—a phenomenon that further underscores morphology‐dependent activity. Variations in the aspect ratio of the NRs alter the ratio of end atoms to side‐surface atoms, thereby modulating the total number and distribution of active sites, and consequently impacting catalytic activity.^[^ ^38^ ^]^ Additionally, Janus nanozymes utilize asymmetric spatial organization to overcome activity constraints of conventional core‐shell structures. The spatial isolation between CeO_2_‐Pt nanozymes and periodic mesoporous organosilica (PMO) allows for maximal exposure of their catalytically active sites. This maximal exposure offers more contact opportunities for reactants, thereby significantly enhancing the efficiency of catalytic reaction, which is the key factor underlying the improved catalytic activity of asymmetric structures. For example, in control experiments versus a conventional core‐shell structure nanoplatform, Janus‐nanoplatform exhibits superior catalytic ability, faster H_2_O_2_ decomposition rate, enhanced O_2_ release ability, higher inhibition rate of superoxide anion (•O_2_^−^) and hydroxyl radical (•OH), and stronger total antioxidant capacity.^[^ ^39^ ^]^ Furthermore, support‐based nanozymes utilize their supports to provide physical scaffolding, enabling uniform dispersion of nanozymes on their surfaces or within internal pores. This facilitates the exposure and dispersion of active sites, enhancing substrate accessibility to nanozyme active centers. Additionally, interactions between nanozymes and their supports boost electron transfer efficiency, thereby improving catalytic activity. For example, CeO_x_ nanoclusters anchored on the Mn_3_O_4_ nanosupports intertwine the CeO_x_ (111) and Mn_3_O_4_ (211) planes, inducing lattice distortion, which underpins rapid electron transfer during the catalytic interplay between CeO_x_ and Mn_3_O_4_, and ensures efficient electron transfer in the catalytic reactions, culminating in the highly efficient nanozyme activity (Figure 2C).^[^ ^32^ ^]^

In conclusion, the morphological diversity of nanozymes and their interaction with supports constitute an intricate system that exerts a profound influence on their catalytic activities. Key factors governing nanozyme catalytic activity include high exposure of active sites in NSs, tunable active site distribution in NRs, distinct spatial effect of Janus morphology, and activity‐enhancing mechanism of support‐based nanozymes. Each of these aspects reveals the intricacies of nanozyme catalysis. These findings not only advance our scientific understanding of this emerging material but also pave the way for the versatile application of nanozymes across diverse fields.

### 2.3 Hollow

Hollow nanostructures offer four key advantages that synergistically enhance nanozyme activity across both molecular and macroscopic scales. They markedly enhance specific surface area, directly facilitating the exposure of more active sites, affording ample adsorption space for substrate molecules, and substantially strengthening interaction between substrate and nanozyme active sites.^[^ ^40^, ^41^ ^]^ Meanwhile, their unique hollow architecture effectively facilitates substrate diffusion, shortening the distance between substrate molecules and active sites, reducing the energy loss and diffusion resistance during diffusion, and allowing the catalytic reaction to proceed more smoothly and efficiently.^[^ ^42^, ^43^ ^]^ Furthermore, hollow structures also exhibit localized effects that stabilize metal atoms and prevent their agglomeration, thereby maintaining the dispersed state of metal atoms, which is a critical factor for preserving the high catalytic activity of nanozyme.^[^ ^44^, ^45^ ^]^ Moreover, the confined space of hollow structures can enrich the substrate molecules, increase the substrate concentration, and locally form a microenvironment with high reactant concentration. This markedly enhances the effective collision probability between reactant molecules, ultimately boosting the overall chemical reaction rate of the nanozyme.^[^ ^46^ ^]^

Hollow nanostructures markedly enhance specific surface area, exposing abundant active sites while creating 3D adsorption spaces for substrate binding. Besides, their unique hollow architecture facilitates substrate diffusion. During the catalytic process, substrate molecules can diffuse rapidly within the hollow structure and access the active site more readily, thereby enhancing catalytic reaction rates.^[^ ^47^ ^]^ For example, the 3D hollow structure of Co‐based hollow nanocages (HNCs), inherited from the parent HNCs and constructed using staggered NSs as building blocks, offered favorable conditions for active site exposure, enhancing substrate accessibility to these active sites, and thereby facilitating the nanozyme‐catalyzed reactions. The hollow architecture also promotes substrate diffusion, allowing substrate molecules to rapidly reach the active site within the nanozyme structure and further enhancing the catalytic reaction rate.^[^ ^48^ ^]^ Cu_1.5_Mn_1.5_O_4_ cage‐like nanospheres featuring hollow, open, and macroporous nanostructures exhibit a specific surface area of 14.4 m^2^g^−1^, providing more catalytic centers to facilitate full contact with substrate molecules. Besides, substrate molecules can easily penetrate the hollow structure to bind to active sites and react, enhancing catalytic efficiency and accelerating the generation of reactive oxygen species (ROS).^[^ ^49^ ^]^ Furthermore, hollow structures shorten the interfacial transport distance between the active site and the substrate, reduce energy loss and diffusion resistance during the substrate and the product transport, and thus enable more efficient nanozyme‐catalyzed reaction.^[^ ^50^, ^51^ ^]^ For example, Mn_2_O_3_ hollow NPs (MHNs) with POD‐like and glucose oxidase (Gox)‐like activities can promote the cascade reaction. Their hollow architecture enables hydrogen peroxide to more readily react with the substrate TMB following the conversion of glucose to gluconic acid and hydrogen peroxide. This is attributed to enhanced proximity effects between reactants, allowing in situ reactions that minimize mass transfer steps and accelerate reaction rates.^[^ ^52^ ^]^ Additionally, hollow nanostructures can stabilize metal atoms, prevent their agglomeration, and maintain the dispersed state of these atoms, which is a critical factor for preserving the high catalytic activity of nanozyme. For example, Fe─Co─Mn single‐atom nanozymes (SAzymes) anchored in hollow carbon nanospheres maintain a highly dispersed state of metal atoms, ensuring enhanced nanozyme catalytic activity (Figure 2D).^[^ ^33^ ^]^ A confined hollow chamber structure provides a confined space for substrate enrichment and reaction, significantly increasing the substrate concentration and forming a local microenvironment with high reactant enrichment. This greatly enhances the effective collision probability of reactant molecules, thereby boosting the overall nanozyme reaction rate.^[^ ^53^ ^]^ For example, due to the confinement effect, substrate concentration is enriched inside the chamber of hollow FeNPs@Co_3_O_4_ HNCs, increasing the nanozyme catalytic reaction rate.^[^ ^54^ ^]^ Liu's group quantified the influence of reactant enrichment on catalytic performance (i.e., the cavity confinement effect of micro/nanoreactor) and proposed the concept of the micro/nanoreactor effect coefficient (*K_@_*) (Equation (1)), which establishes a direct relationship between cavity structure and catalytic performance. *TOF_@_*, *TOF_&_*, and $TOF_{@}^{\prime }$ denote the activity of nanoreactors, the activity of crushed samples, and the additional activity induced by cavity structures, respectively.^[^ ^53^ ^]^

(1) $$K_{@}=\frac{TOF_{@}}{TOF_{\&}}=\frac{TOF_{\&}+TOF_{@}^{\prime }}{TOF_{\&}}$$

These structural advantages collectively enable hollow nanozymes to achieve catalytic efficiencies comparable to natural enzymes, while retaining robustness under harsh conditions. Hollow nanozymes not only optimize the active site distribution and substrate interaction mechanism of nanozyme at the micro level, but also significantly enhance the rate and efficiency of catalytic reaction at the macro level, providing a solid foundation for the design of next‐generation artificial enzymes.

### 2.4 Pore

The strategic integration of pore nanostructures into nanozyme design has revolutionized catalytic systems through four interconnected mechanisms that synergistically enhance nanozyme performance. These structural features foster multifunctional catalytic microenvironments that transcend the limitations of conventional materials. Pore nanostructures markedly increase accessible surface areas, enabling high‐density distribution of active sites on inner channel surfaces, thereby significantly boosting catalytic reaction rate.^[^ ^27^, ^55^ ^]^

Regarding substance transport, porous nanostructures create efficient pathways for substrate molecules to access the active sites and for reaction products to diffuse. This effectively prevents substance accumulation at active sites, ensures continuous, smooth reaction progression, and thereby effectively enhances overall catalytic efficiency.^[^ ^56^ ^]^ Moreover, pore structures can finely tune the electronic structure and local chemical microenvironment of nanozymes, and precisely modulate their enzyme‐like activity.^[^ ^57^, ^58^ ^]^ Meanwhile, the unique confinement effect of porous nanostructures can confine the reaction molecules within a specific space, strengthen intermolecular interactions, and markedly influence the affinity between nanozymes and substrate. This thereby creates favorable conditions for enhancing the selectivity and yield of catalytic reaction, and comprehensively contributes to optimizing and enhancing the catalytic performance of nanozymes.^[^ ^59^, ^60^ ^]^ Numerous active sites of nanozymes are distributed on the inner surfaces of channels, enhancing reactant accessibility to these sites and thereby facilitating nanozyme‐catalyzed reactions.^[^ ^61^ ^]^ Moreover, porous nanostructures facilitate rapid diffusion of substrates to nanozyme active sites and simultaneously promote the diffusion of reaction products, thus increasing the rate of nanozyme‐catalyzed reactions.^[^ ^62^ ^]^ Constructing porous nanostructures represents a promising strategy to overcome kinetic bottlenecks by enhancing substrate diffusion. For example, SnO_2‐x_ NPs with abundant pore structures exhibit a high specific surface area and effectively increase catalytic active sites within hollow cavities. During nanozyme catalysis, the pore structures of SnO_2‐x_ NPs facilitate rapid diffusion of substrates (such as glucose and H_2_O_2_) to their active sites and promote the diffusion of reaction products (such as gluconic acid), thereby increasing the catalytic reaction rate.^[^ ^63^ ^]^ Additionally, mesoporous nanostructures in Mn/Zr‐co‐doped CeO_2_ tandem nanozymes (PHMZCO‐AT)) provide more adsorption and reaction active sites for substrate molecules, enhancing contact between nanozymes and O_2_^•−^ or H_2_O_2_, and promoting the diffusion of substrate molecules and products within the nanozymes, which thereby facilitates high nanozyme catalytic performance. Moreover, porous nanostructures can modulate the electronic structure and local chemical environment of nanozymes, thereby regulating their enzyme‐mimicking activities. In this work, Zr^4+^ and Mn^2+^ were introduced into CeO_2_ to form mesoporous PHMZCO‐AT. This structural modification accelerated the Ce^4+^ to Ce^3+^ redox cycle, enhancing SOD‐like and POD‐like activities while inhibiting CAT‐like activity. This is likely due to Mn doping inducing local structural reconstruction, generating oxygen vacancies, and promoting electron transfer from Mn to surrounding Ce atoms, which increases the number of reduced Ce^3+^ ions (Figure 2E).**^[^** ^34^ ^]^ Additionally, the confinement effect of porous nanostructures can restrict reaction molecules to some extent, confining them to a specific space for reactions. This can modulate the affinity of nanozymes for substrates and their catalytic efficiency.^[^ ^64^ ^]^ For example, cytochrome c (Cyt c) was confined within the mesoporous nanostructures of PCN‐222 metal–organic framework (MOF) NPs. Kinetic studies found that compared with free Cyt c, the affinity of Cyt c confined in PCN‐222 NPs for H_2_O_2_ was significantly enhanced. The *K*_m_ value decreased from 9.6 to 3.7 mM, the *V*_max_ increased slightly, and *V*_max_/*K*_m_ increased by ≈4–5 times, demonstrating that the catalytic efficiency was markedly improved due to the pore confinement effect.^[^ ^59^ ^]^

To sum up, pore nanostructures are undoubtedly a pivotal factor in the field of nanozyme catalysis. By facilitating the distribution of active sites on the inner pore surfaces, aiding the efficient diffusion of substrates and products, finely tuning the electronic structure and chemical environment of nanozymes, and exerting the confinement effect, these structures comprehensively enhance the catalytic performance of nanozymes. This pore‐nanostructure‐property paradigm establishes a versatile framework for engineering next‐generation nanozymes, laying a robust foundation for the widespread advancement and in‐depth application of nanozyme technologies.

## 3 Electronic Structure for Boosting Nanozyme Catalysis

From the intrinsic nature of nanozyme catalysis, electronic structure governs the catalytic activities by dictating substrate interactions, reaction pathways, and energy barriers.^[^ ^35^, ^65^ ^]^ Thus, nanozyme catalytic activity can be tailored by precisely modulating electronic structures, such as *d*‐band center, *e_g_* occupancy, electron spin, electron transfer, defect engineering, and coordination microenvironment, to mimic or surpass natural enzymes. Theoretical frameworks, such as *d*‐band center theory and *e_g_* occupancy descriptors, provide predictive insights into catalytic behavior, while advanced strategies, like spin‐state manipulation and defect engineering, enable atomic‐level control over reaction kinetics. By bridging fundamental electronic structures with nanozyme design, unprecedented catalytic efficiency of nanozymes can be unlocked, providing a roadmap for the rational development of next‐generation nanozymes.

### 3.1 D‐Band Center

The *d*‐band center theory, initially proposed by Norskov et al., can characterize the magnitude of adsorption energy, thereby revealing the interaction strength between nanozyme surfaces and reactants.^[^ ^66^, ^67^ ^]^ Specifically, the position of the *d*‐band center affects adsorption strength, which in turn influences the rate and efficiency of catalytic reactions.^[^ ^68^ ^]^ The shift of the *d*‐band center in nanozymes is critical for regulating their electronic structure, and changes in its position relative to the Fermi level can significantly alter electron donation and back‐donation processes.^[^ ^17^ ^]^ The energy level of the *d*‐band center determines the electron filling degree of the antibonding orbital, thereby governing the stability and strength of adsorption bonding between nanozymes and substrates.^[^ ^69^ ^]^ Such electronic structure modulation not only dictates the adsorption behavior of reactants on nanozyme surfaces, but also exerts a persistent influence on subsequent catalytic steps. A series of processes, from reactant activation to product generation and desorption, is governed by the electronic interaction dominated by the *d*‐band center position, thereby comprehensively regulating nanozyme catalytic activity.

**Figure 3:**
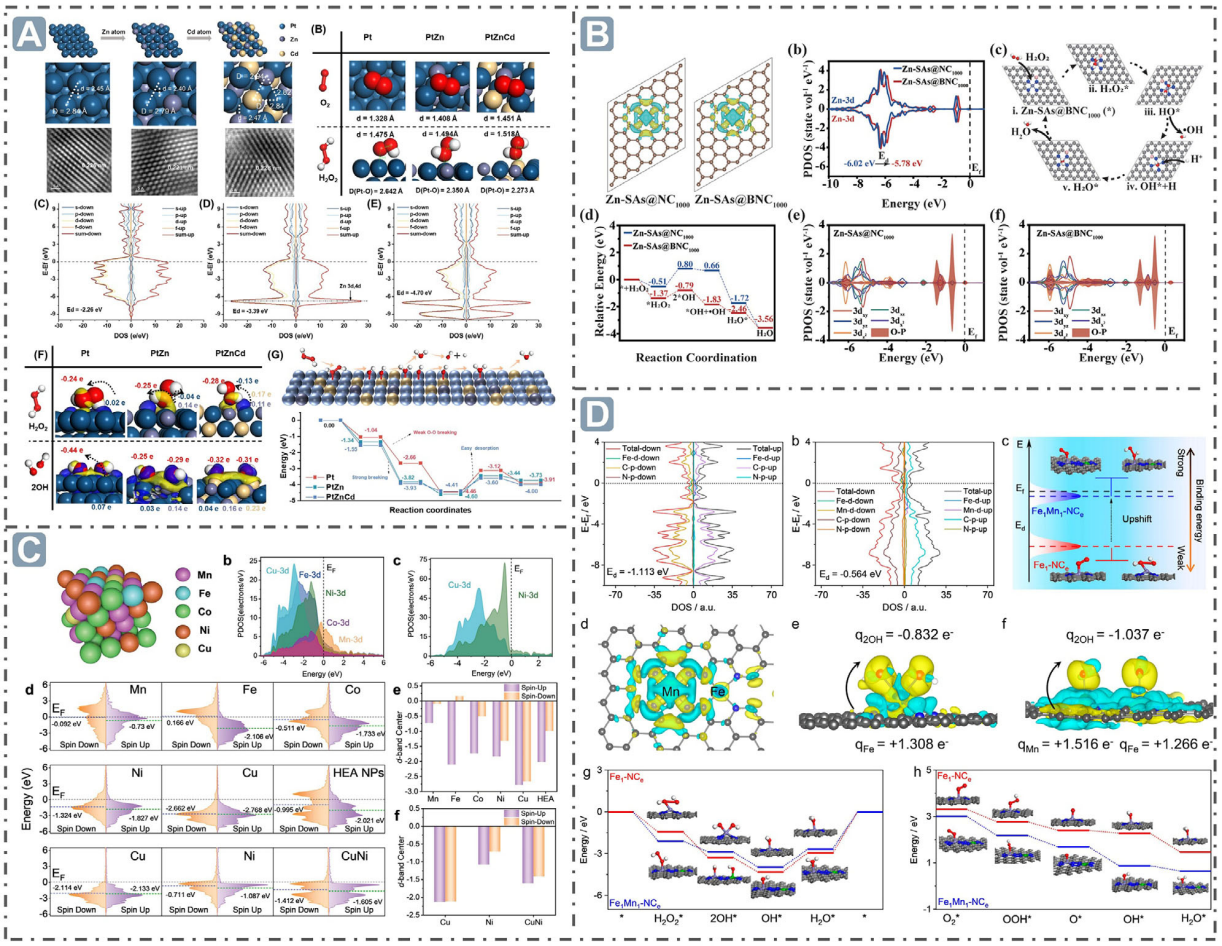
D‐band center optimization for boosting nanozyme catalysis. A) The *d*‐band center‐optimized PtZnCd nanozymes for enhanced nanozyme catalysis. Reproduced with permission.^[^ ^71^ ^]^ *2024 Wiley‐VCH GmbH*. B) B elements‐doped Zn‐SAzymes with the *d*‐band center upshift. Reproduced with permission.^[^ ^72^ ^]^ *2024 Wiley‐VCH GmbH*. C) The HEzymes with significant *d*‐band center obvious upshift. Reproduced with permission.^[^ ^73^ ^]^ *2023 The Authors. Advanced Science published by Wiley‐VCH GmbH*. D) Fe_1_Mn_1_–NC_e_ nanozymes with *d*‐band center upshift at the Fe site. Reproduced with permission.^[^ ^74^ ^]^ Copyright 2024 American Chemical Society.

The shift of *d*‐band center in nanozymes modulates their electronic structures. The position of the *d*‐band center with respect to the Fermi level substantially influences the electron donation and back‐donation processes, which critically affect the adsorption behavior and subsequent catalytic reaction, thereby governing nanozyme catalytic activity.^[^ ^70^ ^]^ For example, the relationship between *d*‐band center position of Zn‐ and Cd‐doped Pt‐based nanozymes and their catalytic activities was studied. As the calculated *d*‐band center shifts negatively, the energy of the potential rate‐determining step (RDS) for the entire system decreases, implying that the most critical thermodynamic step can be tuned by appropriately modulating the *d*‐band center, thereby influencing the catalytic reaction. PtZnCd nanozymes exhibit a more negative *d*‐band center at −4.70 eV, nearly half that of unoptimized Pt nanozymes (‐2.26 eV). Besides, PtZnCd nanozymes show stronger adsorption ability for H_2_O_2_ and more efficient O−O bond cleavage than Pt nanozymes, indicating a positive correlation between substrate adsorption energy and *d*‐band center position, which confirms their enhanced substrate adsorption ability. In terms of reaction rate, PtZnCd nanozymes display higher catalytic efficiency, with a *V*_max_ of 7.289 µM min^−1^ and *K*_m_ of 0.092 mM, whereas Pt nanozymes have a lower *K*cat, indicating a faster reaction rate for PtZnCd nanozymes. Furthermore, the predicted activation energy (*E_a_*) of PtZnCd nanozymes is 8.313 kJ mol^−1^, which is merely one‐third that of Pt nanozymes (24.298 kJ mol^−1^). This significant reduction in *E_a_* renders the reaction more facile (**Figure** **3**A).^[^ ^71^ ^]^ Additionally, the boron (B) element, as an electron‐deficient element, can optimize the electronic structures of Zn atoms, which have a fully occupied 3d^10^ configuration. Upon B doping in Zn‐SAzymes, the Zn atom exhibits increased electron loss, and the *d*‐band center of Zn 3d shifts closer to the Fermi level. This enhances the adsorption capacity of oxygen‐containing intermediates, promotes •OH formation, and renders the nanozyme‐catalyzed reaction more favorable both thermodynamically and kinetics (Figure 3B).^[^ ^72^ ^]^ Besides, the *d*‐electron interactions among different transition metal elements optimize the electron distribution around the Fermi level in transition metal high‐entropy nanozymes (HEzymes) comprising Mn, Fe, Co, Ni, and Cu elements. This enhances the adsorption capacity of intermediates on surface active sites and improves electron transfer efficiency during catalysis, thus enhancing the nanozyme performance. The *d*‐band center of HEzymes is significantly upshifted due to the synergistic effect of different metal elements, promoting the adsorption and stabilization of oxygen intermediates and facilitating the nanozyme catalysis. Notably, a moderate upshift of the *d*‐band center can enhance alloy catalytic activity, while an excessive upshift may exert the opposite effect, which is consistent with the predicted volcanic relationship between the *d*‐band center position of the alloy relative to the Fermi level and its catalytic activity. Although the *d*‐band center of FeCuNi is higher than that of HEzymes, •OH generation on the surface of HEzymes is more energetically favorable, demonstrating that the moderate d‐band center upshift in HEzymes enhances catalytic activity (Figure 3C).^[^ ^73^ ^]^ Moreover, the incorporation of Mn atoms to form Fe–Mn dual‐atom sites (Fe_1_Mn_1_–NC_e_) upshifts the *d*‐band center of Fe sites from −1.113 to −0.564 eV, as revealed by density of states (DOS). This upshift brings the *d*‐band center closer to the Fermi level, enhancing substrate adsorption capacity. Differential charge analysis shows that Fe sites gained electrons upon Mn incorporation, further confirming the shift in the Fe site d‐band center induced by dual‐atom‐site interactions. Charge density difference and Bader charge analysis reveal that 2OH* species accumulate more charge and gain more electrons from Fe_1_Mn_1_–NC_e_ than Fe_1_–NC_e_, indicating stronger substrate affinity for Fe_1_Mn_1_–NC_e_. Moreover, the desorption energy of H_2_O* on Fe_1_Mn_1_–NC_e_ is 2.67 eV, substantially lower than that of Fe_1_–NC_e_ (2.94 eV), facilitating high‐efficiency nanozyme catalysis (Figure 3D).^[^ ^74^ ^]^

In conclusion, the *d*‐band center theory serves an irreplaceable role in studying the electronic structures of nanozymes. Precise regulation of the *d*‐band center position of nanozymes can be achieved by doping specific elements or constructing a high‐entropy system, which effectively tunes the electron donation and back‐donation processes, and optimizes reactant adsorption behavior and catalytic reaction processes. This not only offers a crucial theoretical foundation for in‐depth understanding of nanozyme catalytic mechanisms, but also paves a broad way for designing and developing nanozymes with enhanced catalytic activity, selectivity, and stability.

### 3.2 *e_g_* Occupancy

In an octahedral field, the *d*‐orbitals of a metal ion split into higher‐energy *e_g_* orbitals (composed of $\;d_{z^{2}}$ and $d_{x^{2}-y^{2}}$ and lower‐energy *t_2g_* orbitals (composed of *d_xy_*, *d_yz_*, and *d_zx_*).^[^ ^75^ ^]^ The *e_g_* orbital occupancy refers to the degree of electron filling in the *e_g_* orbitals, typically expressed as the ratio of the number of electrons occupying these orbitals to their maximum electron‐holding capacity.^[^ ^76^ ^]^ The *e_g_* occupancy can modulate the electronic structure of the active center.^[^ ^77^ ^]^ In some transition metal oxide catalysts, the electron‐filling status of the *e_g_* orbitals in metal ions influences their adsorption capacity for reactant molecules. Beyond affecting the adsorption performance of active centers, the *e_g_* occupancy plays a key pivotal in regulating reaction pathways and overall catalytic efficiency more comprehensively. It can determine the balance between different reaction steps and the interaction with reaction intermediates. Additionally, the *e_g_* occupancy impacts the energy barrier of key reactions. An appropriate *e_g_* occupancy can optimize the reaction energy profile, facilitate the formation of reaction intermediates at favorable energy levels, and thereby enhance catalytic activity.

**Figure 4:**
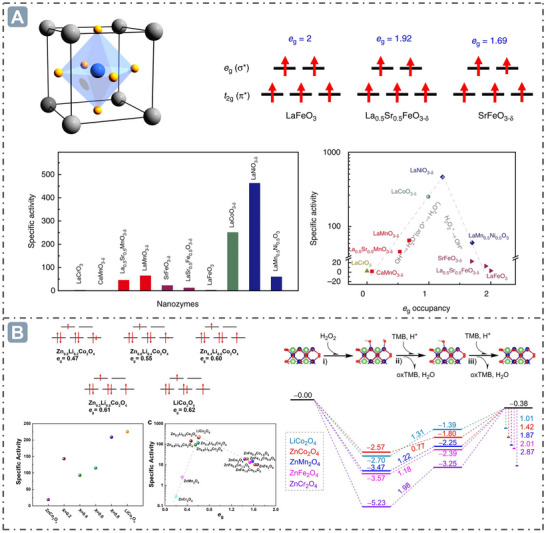
*e_g_* occupancy for boosting nanozyme catalysis. A) The relationship between *e_g_* Occupancy and the POD‐like activity of perovskite oxide nanozymes. Reproduced with permission.^[^ ^82^ ^]^ *Copyright 2019, The Author(s)*. B) The regulation of *e_g_* occupancy for boosting the spinel oxide nanozymes catalysis. Reproduced with permission.^[^ ^78^ ^]^ *Copyright 2022 American Chemical Society*.

The *e*_g_ represent two antibonding molecular orbitals, specifically $\sigma_{x^{2}-y^{2}}^{*}$ and $\sigma_{z^{2}}^{*}$, generated by the splitting of the *d*‐orbitals in an octahedral crystal field, which can be used to describe the catalytic activity of catalysts.^[^ ^78^, ^79^, ^80^, ^81^ ^]^ The *e_g_* occupancy serves as a predictive descriptor for the POD‐like activity of transition metal oxide nanozymes (containing perovskite oxide), with a volcano relationship observed between *e*_g_ occupancy and nanozyme activity. Wei's group first investigated *e_g_* occupancy as a predictive descriptor for POD‐like nanozymes in 2019. Their experiments revealed a volcanic relationship between POD‐like activity and *e_g_* occupancy. Specifically, when *e_g_* occupancy is ≈1.2, the nanozymes exhibited the highest POD‐like activity. The possibility of other parameters, such as the oxidation state of transition metals, 3*d* electron number in B‐site ions, the O 2*p* band center, and B‐O covalence, as candidate descriptors was evaluated. This analysis showed that no significant volcanic relationship between these parameters and POD‐like activity. *e_g_* occupancy influences the POD‐like activity of perovskite‐type transition metal oxide nanozymes by altering the adsorption energy of reaction intermediates and the RDS of catalytic reaction. An *e_g_* occupancy of ≈1.2 enables nanozymes to achieve optimal adsorption energy and effectively facilitate the RDS, thereby yielding high POD‐like activity. Density functional theory (DFT) studies revealed that the calculated adsorption energy (*E_ads_*) for O (*E*_ads, O_) and OH (*E*_ads, OH_) exhibit a volcanic relationship with *e_g_* occupancy. Perovskites with *e_g_* occupancy below 1.2, such as LaCrO_3_, CaMnO_3_, La_0_._5_Sr_0_._5_MnO_3_, LaMnO_3_, and LaCoO_3_, show strong adsorption energies for OH* and O*. In contrast, those with an *e_g_* occupancy above 1.2, such as LaMn_0_._5_Ni_0_._5_O_3_, SrFeO_3_, La_0_._5_Sr_0_._5_FeO_2_._5_,La_0_._5_Sr_0_._5_FeO_3_ and LaFeO_3_, and LaNiO_3_, exhibit weak adsorption energies for OH* and O*. Perovskite with an *e_g_* occupancy of ≈1.2 display the weakest adsorption of OH* and O* species, which facilitates the transfer of oxygen species to TMB substrate, consistent with their highest POD‐like activity. For nanozyme with an *e_g_* occupancy below 1.2, the RDS of the reaction is the oxidation of the substrate. For perovskite with an *e_g_* occupancy above 1.2, the RDS involves the cleavage of the O─O bond in the absorbed H_2_O_2_*. LaNiO_3_ serves as the turning point for this shift in the RDS (**Figure** **4**A).^[^ ^82^ ^]^ Besides, Wei's group also identified a volcanic relationship between the POD‐like activity of spinel oxide nanozymes and *e_g_* occupancy. This work successfully predicted that the *e_g_* occupancy corresponding to the peak catalytic activity of spinel oxide nanozymes is ≈0.6, which was validated by doping Fe and Co at octahedral positions, revealing a strong volcanic curve between *e_g_* occupancy and the POD‐like activity of spinel oxides. Consistent with the prediction based on the *e_g_* descriptor, the *e_g_* value of spinel oxide nanozymes was modulated by introducing stable monovalent lithium at tetrahedral sites to synthesize Zn_1‐x_Li_x_Co_2_O_4_. It was observed that the activity of the synthesized compounds increased with the elevation of doping level. Notably, LiCo_2_O_4_ exhibited the highest POD‐like activity, which was approximately one order of magnitude higher than that of ZnCo_2_O_4_. DFT calculations showed that the reaction energies (*E*_r_) of RDS increased in the order: LiCo_2_O_4_ < ZnCo_2_O_4_ < ZnMn_2_O_4_ < ZnFe_2_O_4_ < ZnCr_2_O_4_. This suggests that *e_g_* occupancy influences the *d*‐band energy on the surface of materials and the adsorption affinity of surface OH*, thereby affecting catalytic activity (Figure 4B).^[^ ^78^ ^]^

In summary, the concept of *e_g_* occupancy has emerged as a powerful tool for understanding and predicting the catalytic behavior of transition metal oxide nanozymes. The discovery of the volcanic relationship between *e_g_* occupancy and catalytic activity in perovskite and spinel oxide nanozymes has opened new avenues for the rational design and optimization of catalytic materials. Future research efforts should focus on further unraveling the underlying mechanisms through which *e_g_* occupancy influences the electronic structure and reaction kinetics at the atomic level. By continuously deepening our understanding of the relationship between *e_g_* occupancy and catalytic performance, we can expect to advance strategies for enhancing nanozyme catalysis.

### 3.3 Electronic Spin

Electron spin is an intrinsic property of electrons, with two states of electron spin typically denoted as by spin‐up (↑) and spin‐down (↓).^[^ ^83^ ^]^ In atomic or molecular systems, the electron spin state can influence electron distribution and energy levels. The electron spin state permeates the interaction network between nanozymes and substrate molecules in a sophisticated and profound manner, profoundly affecting every step of the reaction process, from substrate adsorption and activation, through intermediate transformation, to final product and desorption.^[^ ^23^ ^]^ Notably, the electron spin state plays an irreplaceable guiding role in the rational design of high‐performance nanozymes.

**Figure 5:**
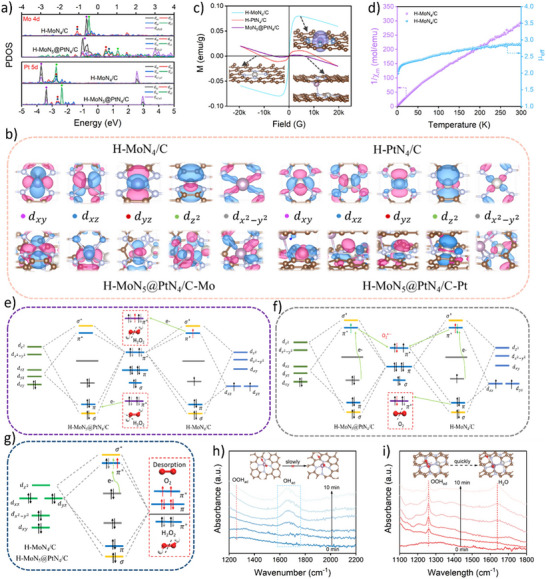
Mechanism of hollow axial Mo‐Pt SAzymes (H‐MoN_5_@PtN_4_/C) with rearranged spin electrons for boosting nanozyme catalysis. Reproduced with permission.^[^ ^84^ ^]^ *Copyright 2024 American Chemical Society*.

Electron spin state influences the interactions between nanozymes and substrate molecules, thereby affecting nanozyme catalytic activity. For example, in the constructed hollow axial Mo‐Pt SAzymes (H‐MoN_5_@PtN_4_/C), a unique structure was formed via a specific synthesis strategy. Within this structure, Mo atoms and Pt atoms coordinate with surrounding N atoms to form porphyrin‐like structures, which induce the splitting of Mo *4d* orbitals and rearrangement of spin electrons from a high‐spin state (*d_xz_* ↑↑*d_yz_*) to a low spin state (*d_xy_* ↑↓). Changes in the spin pairing of single electrons were observed via the superparamagnetic and weakly ferromagnetic characteristics of H‐MoN_4_/C, whereas the diamagnetic contribution of H‐MoN_5_@PtN_4_/C was identified, using vibrating sample magnetometry (VSM) and electron paramagnetic resonance (EPR) spectroscopy. The calculated unpaired spin electrons number (*n*) for H‐MoN_4_/C was about 2 (↑↑), while that for H‐MoN_5_@PtN_4_/C was 0 (↑↓), further confirming the change in electron spin configuration. The change in electron spin configuration contributed to enhanced nanozyme catalysis. The interaction between the empty orbital of the Mo atom in H‐MoN_5_@PtN_4_/C (*d_yz_* and $d_{z^{2}}$) and the occupied π* orbitals of H_2_O_2_ enable cleavage of the H─OOH bond to generate O_2_, whereas H‐MoN4/C activates the HO─OH bond to produce adsorbed OH. This demonstrates that changes in spin electron state can effectively regulate the catalytic activity of SAzymes (**Figure** **5**).^[^ ^84^ ^]^ Besides, electron spin state can also regulate nanozyme catalytic activity by influencing the product desorption step. For example, the electron‐withdrawing properties of Pd nanoclusters (Pd_NC_) can induce a shift in the spin electron occupation of Fe (II) in Fe‐SAzymes (FeNC‐Pd_NC_) from low spin state (LS) to middle spin state (MS). This transition arises from electron transfer due to the electronegativity difference between Fe and Pd, which alters the electron density at the Fe center. According to frontier molecular orbital theory, the catalytic process is influenced by electron transfer between reactants/intermediates and metal centers, and spin‐related electron transfer in the reaction affects both the reaction kinetics and thermodynamics. In the H_2_O_2_ reduction process, the 3d orbits of the Fe site hybridize with the 2p orbitals of O in H_2_O_2_/intermediates, with the 3d electronic state acting as a spin‐dependent “gate” that controls that controls electron transfer and orbital interactions. The half‐filling of $d_{z^{2}}$ orbit in MS Fe (II) reduces the number of valence electrons accepted from adsorbed O species and facilitates σ orbit formation, which promotes the desorption of H_2_O_2_/intermediate to drive the catalytic cycle, enabling spin reconfiguration and nanozyme activity regulation. DFT calculation showed that the heterogeneous dissociation pathway of H_2_O_2_ is favorable for both FeNC and FeNC‐Pd_NC_. For FeNC, the desorption of H_2_O is the RDS and consumes energy, whereas this step is exothermic in FeNC‐PdNC, favoring H_2_O desorption. Additionally, Pd_NC_ reduces the energy barrier for the dehydration of H_2_O_2_ dehydration to form the Fe (IV)O intermediate in FeNC‐PdNC. The spin state transition enhances the oxidation ability of TMB, and Pd_NC_ suppresses TMB dissociation to facilitate its oxidation, collectively demonstrating that FeNC‐Pd_NC_ with a high proportion of MS Fe (II) exhibits high POD‐like activity.^[^ ^85^ ^]^

In conclusion, the electron spin state is a crucial factor regulating the catalytic activity of nanozymes, whose influence permeates every step of the catalytic process, from substrate adsorption and activation to product desorption, as well as the overall reaction kinetics and thermodynamics. The examples of hollow axial Mo‐Pt SAzymes and FeNC‐Pd_NC_ demonstrate how changes in electron spin configuration lead to significant variations in catalytic performance. Understanding and precisely controlling the electron spin state lays the foundation for the rational design and construction of nanozyme systems with specific catalytic activities.

### 3.4 Electrons Transfer

Electron transfer is pivotal in regulating the activity of nanozymes. It permeates every aspect, from fundamentally shaping the catalytic activity and performance of nanozymes, to being intricately intertwined with their structural features, to serving as the central driver underlying the expression of diverse enzyme‐like activities, to playing a critical role in controlling the catalytic activity and selectivity during interaction between nanozymes and substrates, and even extending to the cascade effects induced by the unique electron transfer modes between nanozymes and catalytic intermediates. Electron transfer is ubiquitous and exerts far‐reaching impacts.

**Figure 6:**
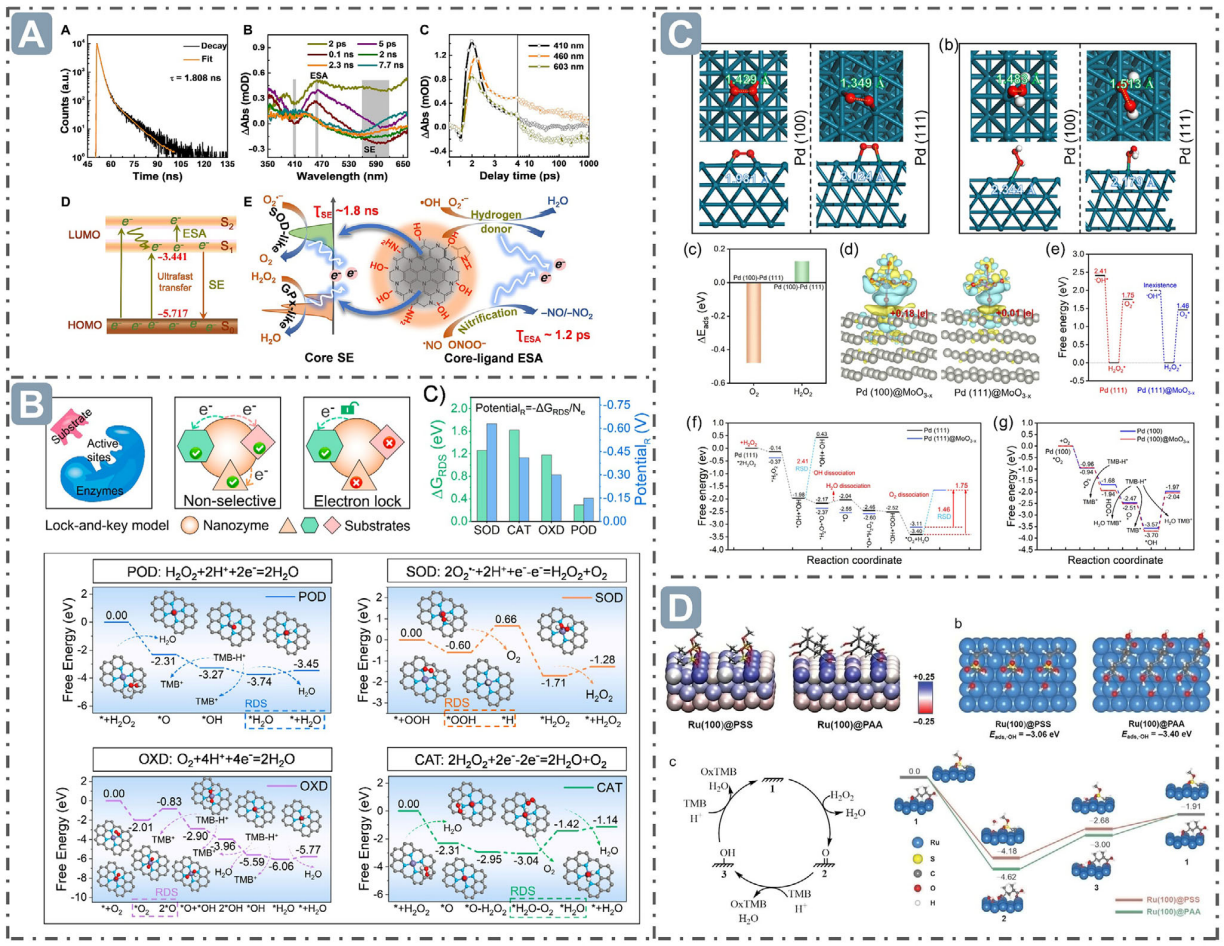
Electron transfer for boosting nanozyme catalysis. A) The oligomeric nanozymes with π‐conjugated domain‐mediated electron transfer for boosting nanozyme catalysis. Reproduced with permission.^[^ ^86^ ^]^ *2021 The Authors*. B) The energy controls the electron transfer between substrates and nanozymes for boosting nanozyme catalysis. Reproduced with permission.^[^ ^87^ ^]^ *Copyright 2024 American Chemical Society*. C) The electron transfer in Pd@MoO_3–_ *_x_* nanozyme for boosting nanozyme catalysis. Reproduced with permission.^[^ ^88^ ^]^ *Copyright 2023 American Chemical Society*. D) Surface ligands modification‐mediated electron transfer for boosting nanozyme catalysis. Reproduced with permission.^[^ ^89^ ^]^ *2023 Wiley‐VCH GmbH*.

In nanozyme catalysis, electron transfer significantly affects the catalytic activity and performance of nanozymes. The π‐conjugated domain of oligomeric nanozymes facilitates charge transfer and electron storage, creating favorable conditions for electron transfer. Moreover, structural defects and surface functional groups, such as amide, hydroxyl, and pyrrolic nitrogen etc., can promote interfacial electron transfer and act as electron donors, jointly facilitating the occurrence of electron transfer. Benefiting from the relationship between electron transfer mechanism and structure, the electron transfer rate reaches 1.8 ns in the internal cores and 1.2 ps between core and ligand molecules, achieving comparable SOD‐like and GSH POD‐like activities (**Figure** **6**A).^[^ ^86^ ^]^ Electron transfer between nanozymes and substrates is crucial to their catalytic activity and selectivity. For example, the energy governs the electron transfer between substrates and nanozymes, thereby controlling their catalytic activities, which is because the electron transfer can only occur when the electron energy in nanozymes is sufficient to overcome the energy barrier. DFT calculation revealed that the free energy of RDS (ΔG_*RDS*_) for the POD‐like, SOD‐like, OXD‐like, and CAT‐like activity of Fe‐N_4_ model is 0.29, 1.26, 1.18, and 1.62 eV, respectively. According to the calculated ΔG_*RDS*_results, RDS potentials (Potential_R_) were inferred that which can give guidance for the development of a matching conduction band to control the electron transfer and achieve the nanozyme catalytic activity (Figure 6B).^[^ ^87^ ^]^ Moreover, MoO_3–_ *_x_* acts as a reversible electron reservoir, acquiring electrons from the decomposition of H_2_O_2_ on Pd (111) or the degradation of GSH, then transferring them to Pd (100) via oxygen bridges or a small number of Mo–Pd bonds. This process enhances the enzyme‐like activity of the dual active centers and GSH degradation ability, while promoting the accumulation of O_2_^•−^. Pd (100) and Pd (111) facets tend to adsorb O_2_ and H_2_O_2_, respectively, which can be attributed to differences in adsorption energy. Consequently, the Pd (100) is primarily responsible for OXD‐like activity, while Pd (111) dominates the CAT‐like activity. Charge density difference and the Bader charge analysis revealed that in Pd (100)@MoO_3–_ *_x_*, MoO_3–_ *_x_* can transfer 0.18|e| electrons to Pd (100) surface, while the transfer amount is only 0.01|e|, strongly supporting the electron transfer theory. Energy barrier analysis also supports the enhancement of nanozyme catalysis via this electron transfer mechanism (Figure 6C).^[^ ^88^ ^]^ Furthermore, electron transfer also influences the absorption energy of the intermediate to regulate the nanozyme catalytic activity. For example, electron transfer between Ru and surface ligands can significantly modulate POD‐like catalysis. DFT calculations indicated that polymers, such as PSS and PAA, interacting with Ru induce the electron transfer from Ru sites to polymers, rendering Ru sites lacking electrons and weakening the affinity for •OH (*E_ads,_*_•OH_) to boost POD‐like catalytic activity, which was verified by electron spin resonance (Figure 6D).^[^ ^89^ ^]^

To sum up, electron transfer holds a pivotal role in the field of nanozymes, exerting a profound and multifaceted influence on their functions. Not only does it play an essential role in regulating the catalytic activity and performance of nanozymes, but it is also closely associated with the structural features. In addition, the interaction between nanozymes and substrates is largely dependent on electron transfer, with parameters such as energy and free energy playing a decisive role in shaping catalytic selectivity and activity.

### 3.5 Defect Structure

Defect structure refers to region in nanomaterials where atomic arrangements deviate from the ideal lattice structure.^[^ ^90^ ^]^ In nanozymes, the presence of such defects can induce local changes in the atomic environment and modifications to the electronic state.^[^ ^91^ ^]^ Various types of defects, such as vacancy defects, substitutional defects, and strain defects, act like opening new avenues for improving the nanozymes catalytic performance. They can alter the electronic structure of nanozymes, adjust their lattice environment, and optimize the energy states of substrates and intermediates. Consequently, these defects exhibit notable activity‐enhancing effects in various simulated catalytic reactions, offering new prospects for boosting the catalytic performance of nanozymes.

**Figure 7:**
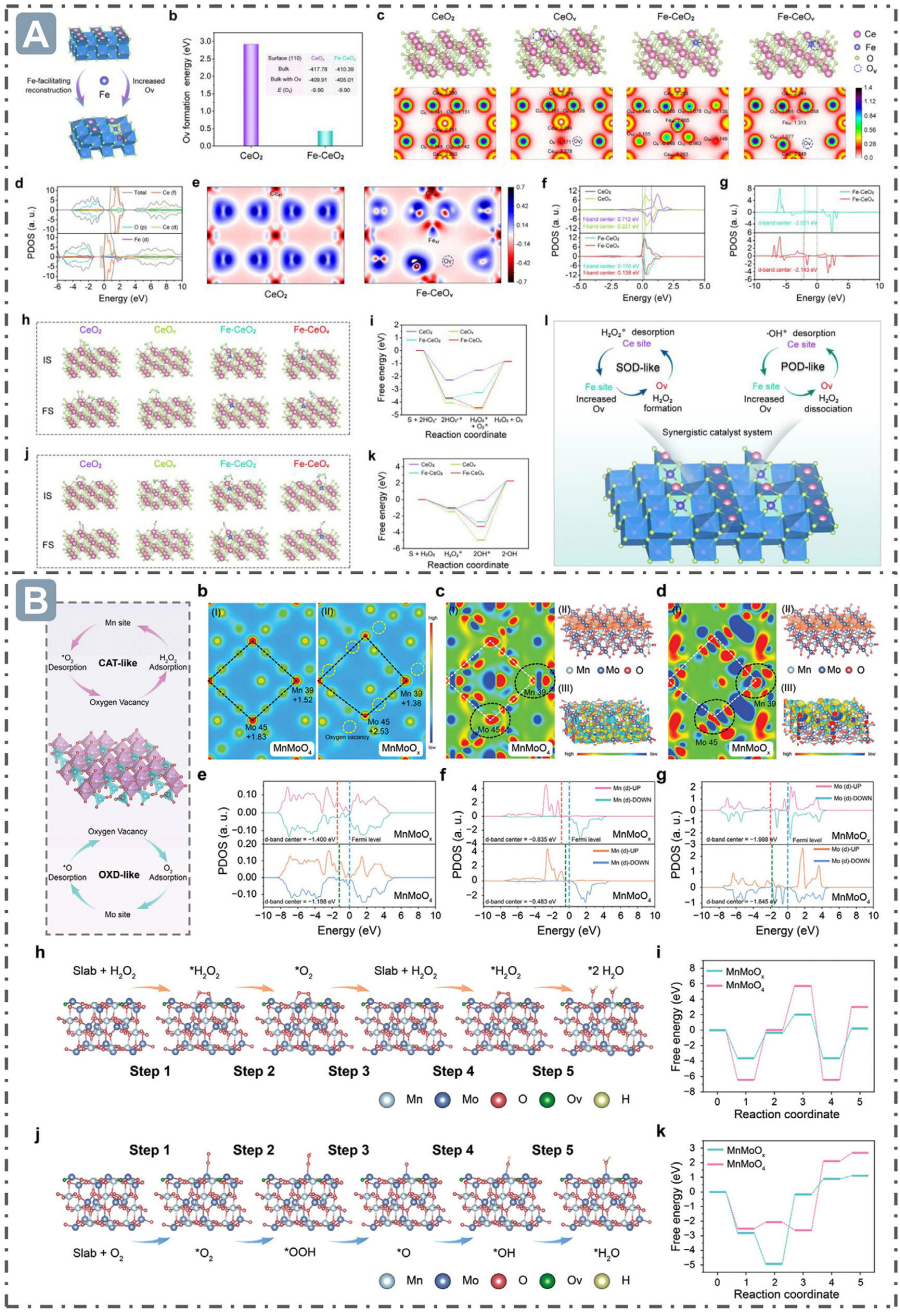
Defect structure for boosting nanozyme catalysis. A) Defect structure through Fe‐doped into the CeO_2_ lattice for boosting nanozyme catalysis. Reproduced with permission.^[^ ^94^ ^]^ *Copyright 2023 American Chemical Society*. B) MnMoO_x_ nanozymes with strain‐defect through lattice distortion for boosting nanozyme catalysis. Reproduced with permission.^[^ ^99^ ^]^ *2024 Wiley‐VCH GmbH*.

Vacancy defects refer to defects formed by the absence of a certain atom (or ion) at lattice points in the crystal structure of nanozymes, and can effectively regulate the nanozyme catalytic activity.^[^ ^92^, ^93^ ^]^ For example, doping Fe into the CeO_2_ lattice enables the formation of a unique Ce─O─Fe arrangement on the surface of Fe‐CeO_2_, reducing the formation energy of oxygen vacancies (O_v_) from 2.92 eV on the surface of ideal CeO_2_ to 0.44 eV, indicating that O_v_ is more easily generated. Besides, Fe introduction shortens the Ce–O bond length and lengthens the Fe─O bond length, which not only induces electron redistribution but also alters the valence state of the surface Ce ions. Projected density of states (PDOS) analysis revealed Ce^4+^→Ce^3+^ reduction on the Fe–CeO_v_ surface, which was further confirmed by X‐ray photoelectron spectroscopy (XPS) results. Moreover, the band centers of both Fe and Ce on the Fe–CeO_v_ surface are significantly downshifted, thereby markedly increasing the adsorption energy of intermediate species and enhancing the catalytic activity. Thermodynamically, for SOD‐like activity, the HO_2_^•−^ radical is strongly adsorbed on the surfaces of CeO_v_ and Fe‐CeO_v_ (with adsorption energies (△G_ads_) of −4.06 and −3.71 eV, respectively), followed by a disproportionation reaction to generate H_2_O_2_ and O_2_ (with exothermic energies (△G_a_) of −0.45 and −0.72 eV, respectively). However, on the surfaces of CeO_2_ and Fe‐CeO_2_ without O_v_ structures, the energy barriers for this reaction are relatively high (0.75 and 0.38 eV, respectively). For POD‐like activity, the △G_ads_ of CeO_v_ and Fe–CeO_v_ for H_2_O_2_ molecule are −1.49 and −0.99 eV, respectively, and △G_a_ for O─O bond cleavage on CeO_v_ and Fe–CeO_v_ are −3.46 and −2.31 eV, respectively, indicating the H_2_O_2_ molecules are more easily absorbed and desorbed at the O_v_ sites (**Figure** **7**A).^[^ ^94^ ^]^ Besides, doping other atoms into the lattices of nanozymes to construct substitutional defects can alter their electronic structures and chemical properties.^[^ ^95^ ^]^ For example, Cu‐doped MoO_x_ with oxygen defect structures can generate substitutional defects and affect the nanozyme's structure, such as changes in Mo─O bond energy, local lattice environment, electronic structure, and oxygen defect ratio. For the CAT‐like activity, DFT calculations revealed that Cu substitutional defects can induce surface charge redistribution, reducing the free energy of OH* desorption (ΔG_OH*_). For the Cu‐MoO_x_ structure with oxygen defects, the calculated initial state energy for the adsorption and activation of H_2_O_2_ molecules was −2.31 eV, compared to −3.89 eV for the MoO_x_ structure, indicating that Cu substitutional defects contribute to enhancing the catalytic activity. Additionally, Cu substitution in MoO_x_ helps provide free electrons and supports the surface plasmon resonance effect, which enhances OXD‐like activity.^[^ ^96^ ^]^ Besides, N‐doped carbon nanozymes were prepared using polyethylenimine as the carbon and nitrogen sources and natural clay minerals as the hard template, respectively. The POD‐like activity of these nanozymes can be regulated by pyrolysis temperature and polyethylenimine dosage, due to the formation of different defects under varying conditions, evidenced by the intensity ratio of Raman peaks at 1360 and 1580 cm^−1^.^[^ ^97^ ^]^ Moreover, strain‐defects can regulate the nanozyme activity by inducing oxygen defects in nanomaterials.^[^ ^98^ ^]^ For example, oxygen defects in MnMoO_x_ nanozymes are generated by strain‐defect through lattice distortion, making the oxygen atom in a metastable state to form oxygen defects at high temperature. Metal glycerates exhibit a lamellar structure, where metal hydroxide sheets are stacked and interspersed with glycerate ions. As temperature increases, organic components decompose, and stress is generated via extrusion and accumulation between layers, inducing strain and consequently leading to lattice distortion at the microscopic scale. Bader charge analysis revealed that the Bader charge values of Mn and Mo in MnMoO_x_ differ from those in MnMoO_4_ due to variations in electron transfer. The presence of O_v_ in MnMoO_x_ alters its electronic structure, resulting in large surface charge accumulation regions that can donate and accept electrons to optimize the free energy of substrate adsorption and reduce the formation energy of intermediates. The downshift of the *d*‐band center of MnMoO_x_ facilitates the desorption of catalytic products from the catalyst surface, enabling MnMoO_x_ to exhibit superior performance in CAT‐like and OXD‐like catalytic reactions (Figure 7B).^[^ ^99^ ^]^

In summary, the exploration of defect structures in nanozymes has emerged as a dynamic and promising research area. Vacancy defects, substitutional defects, and strain defects each have distinct mechanisms of action, and all have demonstrated the ability to precisely modulate the catalytic performance of nanozymes. By tuning the electronic structure, modifying the lattice environment, and optimizing the energy states, these defects effectively enhance the activity of nanozymes in various simulated catalytic reactions.

### 3.6 Coordination Microenvironment

The coordination microenvironment occupies a central position in nanozyme research, serving as a critical factor that intricately links the electronic structure of nanozymes to their catalytic activity.^[^ ^100^, ^101^ ^]^ By emulating the coordination microenvironment of natural enzymes, researchers can optimize the electronic structures of nanozymes, thereby enhancing their interaction with substrates.^[^ ^102^ ^]^ This approach has opened up new avenues for tailoring nanozyme performance to rival or even surpass that of their natural counterparts. Tuning the coordination number of metal atoms enables the achievement of optimal electronic configuration between the active center and ligands, thereby improving nanozymes’ substrate recognition and catalytic ability.^[^ ^18^ ^]^ In addition, optimizing the coordination environment can enhance oxygen activation ability and facilitate the nanozyme catalytic process. Furthermore, regulating the second coordination microenvironment can modulate electronic structure and promote electron transfer, which is more conducive to substrate adsorption and key reaction steps. Accordingly, optimizing the coordination microenvironment of nanozymes offers a promising opportunity to boost nanozyme catalysis.

**Figure 8:**
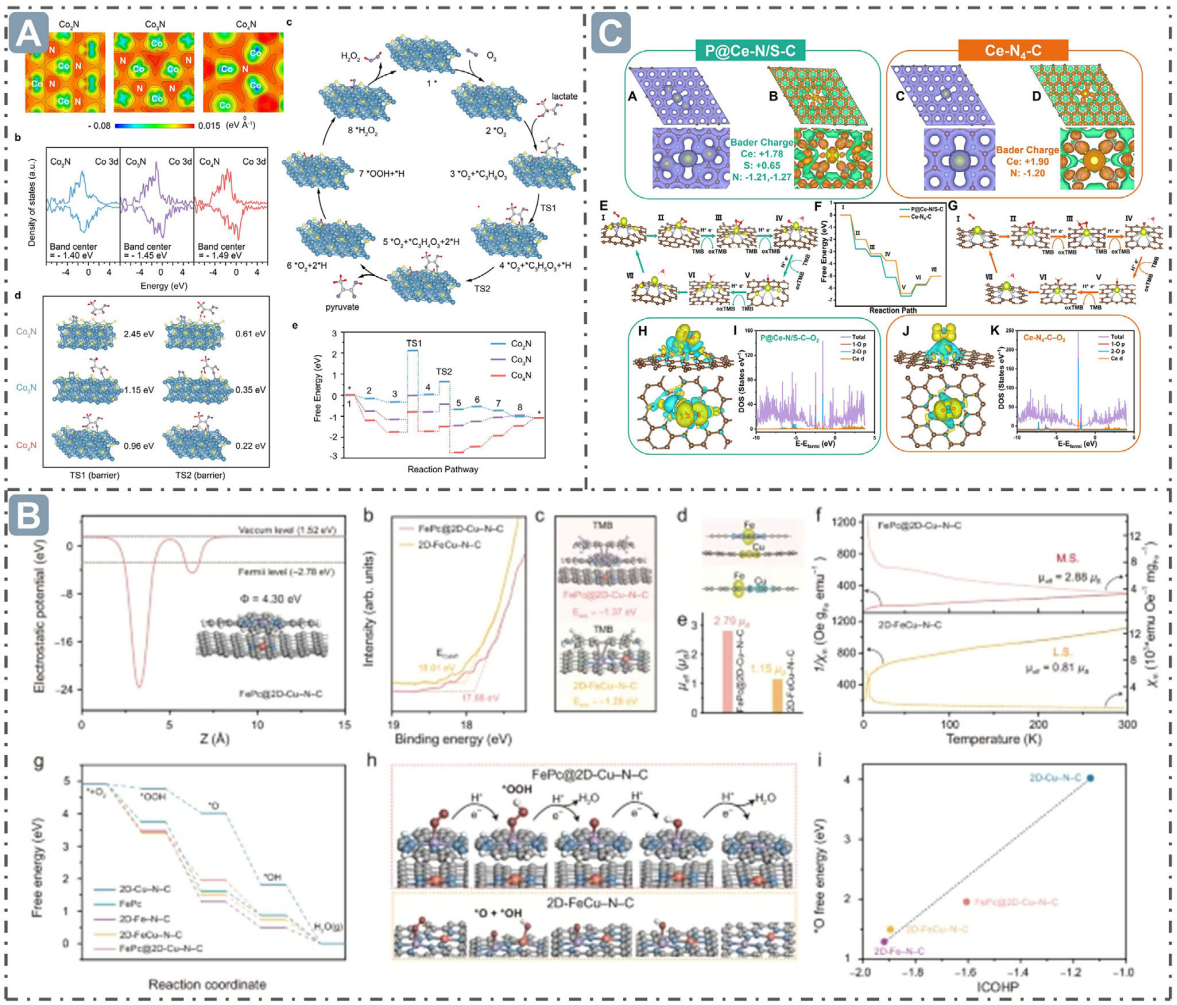
Coordination microenvironment for boosting nanozyme catalysis. A) Coordination microenvironment regulation through the coordination number of the metal Co atoms for boosting nanozyme catalysis. Reproduced with permission.^[^ ^104^ ^]^ *Copyright 2023 American Chemical Society*. B) Vertically stacked Fe–N_4_ and Cu–N_4_ geometry (FePc@2D‐Cu–N–C) for boosting nanozyme catalysis. Reproduced with permission.^[^ ^106^ ^]^ *Copyright 2024, The Author(s)*. C) Second coordination layer of Ce‐SAzymes for boosting nanozyme catalysis. Reproduced with permission.^[^ ^109^ ^]^ *Copyright 2024 American Chemical Society*.

By mimicking the coordination microenvironment of natural enzymes, nanozymes can be developed to optimize their electronic structures and enhance interactions with substrates.^[^ ^103^ ^]^ For Co_4_N/C nanozymes, their electronic structure characteristics play a crucial role in lactate oxidase (LOX)‐mimicking activity and catalytic process. Tuning the coordination number of the metal Co atoms enables the achievement of an optimal electronic configuration between non‐metal N active centers and metal Co ligands, thereby enhancing the recognition and catalytic ability towards lactate. As the number of Co atoms coordinated with N increases, the electron density at the N center in Co_2_N, Co_3_N, and Co_4_N gradually rises, facilitating the abstraction of α‐C–OH and α‐C–H protons from lactate. The *d*‐band center of Co in the Co_4_N model is positioned lowest, accelerating the RDS. Experiments showed that the Co_4_N/C nanozymes exhibit a low *E_a_* (7.77 kJ mol^−1^) during the lactate catalytic oxidation, with lactate consumption ability increasing with pH and temperature. DFT calculations indicated that the catalytic process of the Co_4_N/C nanozymes follows a Ping‐Pong‐like mechanism, based on the carbanion formation mechanism of natural LOX. The N atom of Co_4_N abstracts protons from lactate to form transition states TS1 and TS2. Subsequently, pyruvate is released, and O_2_ oxidizes the H atom into H_2_O_2_, restoring Co_4_N to its initial state (**Figure** **8**A).^[^ ^104^ ^]^ Besides, Cu atoms in the interlayer and in‐plane of poly (heptazine imide) (donated as Cu_L_/PHI and Cu_P_/PHI, respectively) exhibit distinct coordination environments (CuN_4_ and CuN_2_, respectively). Cu_L_/PHI shows higher OXD‐like activity than CuP/PHI, which can be attributed to the stronger O_2_ activation ability. The activation energy barrier of CuL/PHI is 0.52 eV, lower than the 1.08 eV of CuP/PHI. Moreover, electrons at the Cu site in Cu_L_/PHI are more concentrated on O_2_, demonstrating that optimizing the coordination environment enhances nanozyme catalysis.^[^ ^105^ ^]^ Furthermore, compared to single‐site SAzymes, dual‐site SAzymes can better simulate the function of natural enzymes through the specific active‐site topology and microenvironments. A vertically stacked Fe–N_4_ and Cu–N_4_ geometry (FePc@2D‐Cu–N–C) was prepared by assembling FePc molecules on 2D‐Cu‐N‐C, with a configuration similar to that of natural Cyt c oxidases. Work function calculations exhibited that the work function (4.30eV) of FePc@2D‐Cu‐N‐C is higher than that of 2D‐FeCu‐N‐C, which was confirmed by the cut‐off energy measured via ultraviolet photoelectron spectroscopy (UPS), showing that the electron transfer of FePc@2D‐Cu‐N‐C from substrate to metal site is more favorable. The absorption energy of FePc@2D‐Cu‐N‐C toward TMB is higher than that of 2D‐FeCu‐N‐C, and the positive spin moment is mainly concentrated at the Fe site, which facilitates oxygen adsorption and intermediate formation. Fe‐N_4_‐O is the key intermediate, and the reaction follows the pathway O_2_ → OOH → O* → OH* → H_2_O. Analysis of adsorption energies and crystal orbital Hamiltonian populations explains the difference in catalytic activity (Figure 8B).^[^ ^106^ ^]^ The asymmetric electronic distribution of nanozymes can effectively optimize the free energy of each transition state, exhibiting superior enzyme‐like catalytic activity compared to their symmetrical counterparts, because the strong electron‐donating effect of additional axial coordinating atoms.^[^ ^107^ ^]^ Ir‐N_5_ SAzymes feature a unique electronic structure with a coordination structure is Ir‐N_5_, distinct from the symmetric Ir‐N_4_ SAzymes. This asymmetric electron distribution enables a synergistic effect between the central Ir atom and the axial N‐coordination structure, optimizing the free energy of the transition state. X‐ray absorption near‐edge structure (XANES) and K‐edge extended X‐ray absorption fine structure (EXAFS) spectroscopy showed that the average valence state of Ir in both Ir‐N_5_ and Ir‐N_4_ SAzymes lies between Ir⁰ and Ir⁴⁺, confirming the presence of only isolated Ir single atoms, and the introduction of melamine promotes the formation of the Ir‐N_5_ structure. DFT calculations indicated that in OXD catalysis, Ir‐N_5_ SAzymes exhibit a stronger ability to adsorb O_2_ and weaken the O‐O bond, with a low free energy of *O, enabling more efficient cleavage of the O─O bond in *OOH. In POD‐like catalysis, they show strong adsorption of H_2_O_2_ and a low energy barrier for *OH formation. DOS analysis showed that Ir‐N_5_ SAzymes have a high density of states near the Fermi level, leading to a stronger substrate interaction and more efficient charge transfer.^[^ ^108^ ^]^ Moreover, regulating the secondary coordination microenvironment of nanozymes can co‐regulate their electronic structures and enhance electron transfer efficiency.^[^ ^9^ ^]^ Embedding S atoms in the secondary coordination layer of Ce‐SAzymes to construct the Ce‐N_4_S_2_‐C SAzymes, which enhances the interaction between O_2_ and the orbitals of the central atom, reducing the reaction energy barrier and facilitating charge transfer. Bader charge analysis revealed that the Bader charge of the Ce atom in the P@Ce–N/S–C is +1.78, lower than that in Ce–N_4_–C (+1.9), indicating stronger O_2_‐reducing ability in P@Ce–N/S–C. Additionally, higher electrons in the *d* orbitals of Ce atoms in P@Ce–N/S–C is demonstrated by the PDOS analysis. Thermodynamically, in the OXD‐like reaction of P@Ce–N/S–C, O_2_ first forms O_2_^•−^ and then binds to the Ce site. After multiple reaction steps, it returns to its initial state, a spontaneous exothermic process. P@Ce–N/S–C is more favorable for O_2_ adsorption and *OOH cleavage, with structural, charge, and orbital analyses all confirming its r advantages in the reaction (Figure 8C).^[^ ^109^ ^]^

In summary, exploring the coordination microenvironment in nanozyme research holds profound significance, as it serves as a cornerstone for enhancing nanozyme performance, underpinning the optimization of their electronic structures and catalytic capabilities. Whether through adjusting the coordination number of metal atoms, optimizing the overall coordination environment, or regulating the second‐layer coordination, such fine‐tuning of the coordination microenvironment can markedly enhance nanozyme catalytic activity. This not only deepens our understanding of the structure‐function relationship in nanozymes but also lays the groundwork for designing and developing more efficient, versatile, and cost‐effective nanozyme‐based catalytic systems.

## 4 External Stimulation for Boosting Nanozyme Catalysis

In the field of nanozyme research, regulating their catalytic activity via external stimulations has emerged as a key research direction.^[^ ^110^, ^111^ ^]^ Owing to their unique physicochemical properties, nanozymes exhibit great application potential across diverse fields; thus, accurately regulating their catalytic activity to meet varied application requirements is of great significance. External stimulations, such as US, light, electricity, magnetic field, X‐ray, and thermal energy, exert profound impacts on nanozyme catalytic activity through their unique and complex mechanisms. The interaction between these stimulations and nanozymes encompasses multiple aspects, from altering the microstructure and electronic state of nanozymes to influencing substrate diffusion and adsorption to participating in electron transfer and ROS generation processes during catalytic reactions. In‐depth investigation into the regulatory mechanism of these external stimulations on nanozyme catalytic activity not only helps reveal the intrinsic nature of nanozyme catalysis but also provides a solid theoretical foundation and innovative technical means for their efficient application in biomedicine, food science, environmental science, energy, and other fields. This, in turn, will promote further development and breakthroughs in nanozyme technology.

### 4.1 Ultrasonic Stimulation

In the frontier field of nanozyme catalysis, US stimulation, as an influential external intervention method, is gradually becoming a research hotspot.^[^ ^112^, ^113^ ^]^ US stimulation does not act simply on the nanozyme systems, but has a subtle interaction with the unique properties of the nanozyme, particularly piezoelectric properties.^[^ ^114^ ^]^ This interaction has brought new opportunities and breakthroughs for improving the catalytic activity of nanozyme. For pressure‐sensitive nanozymes, such as piezoelectric nanozymes, US stimulation can penetrate into their microstructure and trigger a series of chain reactions. From generating enhanced built‐in electric field to promoting electron‐hole separation, to affecting the diffusion of substrate in solution and the mode of contact with nanozyme, US stimulation affects the catalytic process of nanozyme in a comprehensive manner.^[^ ^115^, ^116^ ^]^ Meanwhile, US stimulation also exerts unique effects in other types of nanozyme systems, such as promoting the separation of electrons and holes and preventing their recombination, assisting nanozyme in acting as an electron trap site, and altering nanozyme conformations to render their active sites more conducive to substrate binding.^[^ ^117^, ^118^ ^]^ Additionally, US stimulation can also release energy to accelerate the conversion of reaction intermediates and promote the generation of ROS in cooperation with nanozyme and US sensitizer.^[^ ^117^, ^118^ ^]^ These multi‐dimensional mechanisms collectively form a complex landscape of interaction between US stimulation and nanozyme, opening up new ways for further understanding and optimizing the catalytic performance of nanozyme.

**Figure 9:**
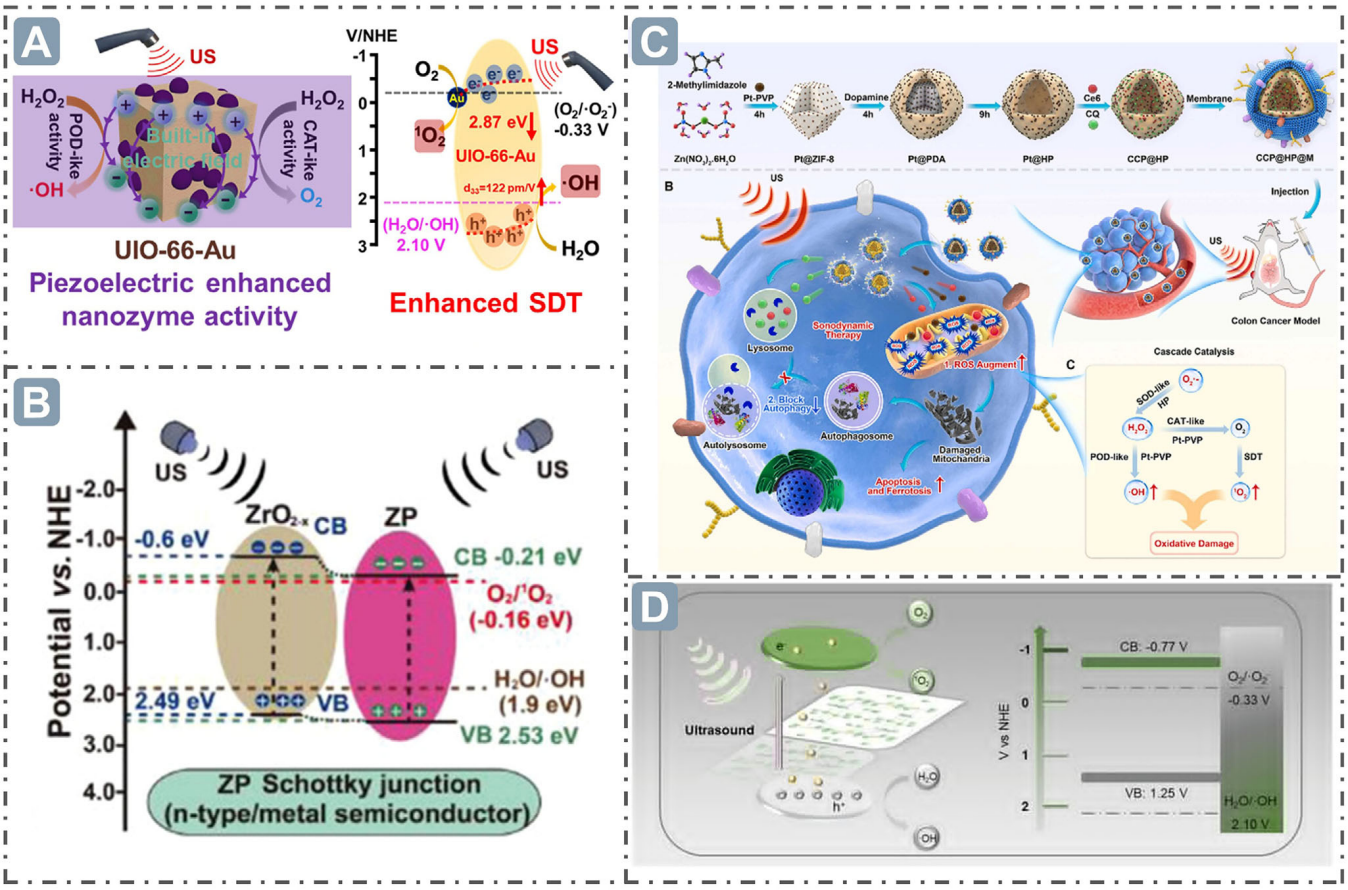
US stimulation for boosting nanozyme catalysis. A) Ultrasonic stimulation‐mediated the piezoelectric UIO‐66‐AuNPs for boosting nanozyme catalysis. Reproduced with permission.^[^ ^19^ ^]^ *Copyright 2023 American Chemical Society*. B) ZrO_2−_ *_x_* @Pt NPs with ultrasonic irradiation to boost nanozyme catalysis. Reproduced with permission.^[^ ^123^ ^]^ *2022 Wiley‐VCH GmbH*. C) Pt@HP loaded sonosensitizer for boosting nanozyme catalysis. Reproduced with permission.^[^ ^124^ ^]^ *2023 Elsevier Ltd. All rights reserved*. D) Ultrasound irradiation‐accelerated the transformation of the reaction intermediate to boost nanozyme catalysis. Reproduced with permission.^[^ ^127^ ^]^ *2022 Wiley‐VCH GmbH*.

US stimulation can interact with the piezoelectric properties of pressure‐sensitive nanozyme. Under the action of the US, the built‐in electric field generated by nanozymes via the piezoelectric effect can be enhanced, which is conducive to accelerating the separation of electrons and holes. For a catalytic reaction, more separated electrons and holes can participate in redox reactions with the substrate. For example, piezoelectric Hf‐based metal‐organic framework (MOF) (UIO‐66) was coupled with AuNPs to boost the CAT‐like and POD‐like activities of AuNPs. The built‐in electric field of UIO‐66 can be generated by US irradiation to promote the separation of electron–hole and generate ROS. UIO‐66 exhibited a piezoelectric coefficient of 71 pmV^−1^, higher than conventional piezoelectric materials, such as BaTiO_3_ (40 pmV^−1^) and ZnO (12.4 pmV^−1^). After depositing AuNPs on UIO‐66, the piezoelectric coefficient increased to 122 pmV^−1^, as AuNPs enhanced the imbalance of charge distribution on UIO‐66. By virtue of the piezoelectric field generated under US irradiation, the catalytic activity of UIO‐66‐AuNPs nanozymes was improved twofold compared with bare AuNPs (**Figure** **9**A).^[^ ^19^ ^]^ Additionally, MoS_2_ NSs were integrated with piezoelectric tetragonal barium titanate (T‐BTO) to boost the POD‐like nanozyme catalysis through reducing the binding energy between MoS_2_ and H_2_O_2_ and promoting the dissociation of H_2_O_2_ via positive and negative charges from T‐BTO under US irradiation.^[^ ^119^ ^]^ The cavitation effect of US stimulation can enhance the diffusion of substrate in solution and enabling more thorough contact between substrates and nanozymes. This not only increases the collision frequency between substrate and nanozyme, but also improves the reaction efficiency, thus efficiently boosting nanozyme catalytic activity. For example, sonosensitizer *meso*‐tetra(4‐carboxyphenyl)porphine (T790)‐modified Pd@Pt nanoplates were developed that can inhibit the CAT‐like activity of Pd@Pt nanoplates. Their CAT‐like activity can effectively recover upon the existence of US stimulation to catalyze the decomposition of H_2_O_2_ to generate O_2_. The blocking CAT‐like activity of Pd@Pt nanoplates could possibly be attributed to the diffusion hindrance of H_2_O_2_ by T790 for contacting with nanozymes. Upon the US irradiation, the diffusion of H_2_O_2_ increases, thereby enhancing its contact with H_2_O_2_ and nanozyme. This US‐switchable nanozyme activity can effectively increase the application targeting and reduce the nanozyme side effects.^[^ ^120^ ^]^ Similarly, US irradiation, as an exogenous energy input, can increase collision and interaction between H_2_O_2_ and active sites of CaF_2_ nanozymes, ultimately enhancing the catalytic activity of nanozymes.^[^ ^121^ ^]^ US stimulation can effectively promote the separation of electrons and holes, and nanozyme can act as a role of an electron trap site to hinder the recombination of the electron/hole pair recombination.^[^ ^122^ ^]^ For example, ZrO_2−_ *_x_* @Pt NPs were used as nano‐sonosensitizers, with a bandgap of 2.74 eV compared with 3.09 eV of the ZrO_2−_ *_x_*. The reductive cocatalyst Pt acts as an electron sink to hinder the recombination of electron/hole pair, and the narrow bandgap of ZrO_2−_ *_x_* @Pt NPs enhances the charge utilization efficiency. Under US stimulation, the generated electron/hole pair can initiate the redox reactions on the surface of ZrO_2−_ *_x_* @Pt NPs, and O_2_ molecules are converted into •O_2_^−^ by the hot excited electrons and reacted with holes in the valence band to generate ^1^O_2_. The US irradiation also enhances ^1^O_2_ generation via local oxygenation. Additionally, the valence band edge of ZrO_2_₋_x_@Pt NPs is higher than the 1.9 eV redox potential of H_2_O/•OH couple, which facilitates •OH generation (Figure 9B).^[^ ^123^ ^]^ Additionally, nanozyme can also be loaded with sonosensitizer to synergetically enhance the nanozyme catalysis. Pt@hollow polydopamine (Pt@HP) NPs were incorporated with sonosensitizer, chlorine e6 (Ce6), to enhance the ROS generation under US irradiation (Figure 9C).^[^ ^124^ ^]^ The transmission of US vibration to nanozyme molecules can induce conformational changes in nanozyme, which may render the spatial structure of active sites more conducive to substrate binding. For example, zeolitic imidazolate framework (ZIF‐8) was used to rapidly lock the active conformation of horseradish peroxidase that was treated by US irradiation through a one‐pot immobilization method. Molecular dynamics simulations and experimental testing demonstrated that the active conformation within the metalloenzymes was changed from “close” to “open” after the treatment of US irradiation. This efficiently enhanced the catalytic activity of ZIF‐8/enzyme nanohybrids because of enhanced substrate‐enzyme binding.^[^ ^125^ ^]^ US irradiation can release energy to accelerate the conversion of the reaction intermediate, thereby boosting nanozyme catalysis.^[^ ^126^ ^]^ For example, US irradiation boosts the catalytic activity of LaFeO_3_ perovskite nanocrystals with multiple enzyme‐like activities by accelerating the transformation of the intermediate complex to Fe^2+^. Under US irradiation, a one‐electron oxidation step occurred between Fe^3+^ and H_2_O_2_, converting Fe^3+^ into Fe^2+^, simultaneously, the intermediate (Fe^−^OOH^2+^) was converted into HOO•, which facilitates the generation of •OH radical from the reaction of Fe^2+^ and H_2_O_2_. The reaction steps are expressed (Figure 9D)^[^ ^127^ ^]^:

(2) $$Fe^{3+}+H_{2}O_{2}\to Fe^{-}OOH^{2+}+H^{+}$$

(3)

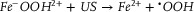



(4)

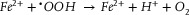



(5)

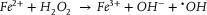




In conclusion, US stimulation has demonstrated extraordinary potential and value in the field of nanozyme catalysis. It can enhance the catalytic activity of nanozyme through multiple interactions by interacting with the piezoelectric properties of piezoelectric nanozyme, enhancing the built‐in electric field, promoting the diffusion of substrate through cavitation effect, increasing the contact between substrate and nanozyme, assisting electron‐hole separation and utilization at the electronic level, changing the conformation of nanozyme to optimize substrate binding, and accelerating the transformation of reaction intermediates. These research results not only deepen our understanding of the interaction mechanism between ultrasound and nanozyme, but also provide solid theoretical support and practical guidance for the application and expansion of nanozyme in more fields.

### 4.2 Photostimulation

**Figure 10:**
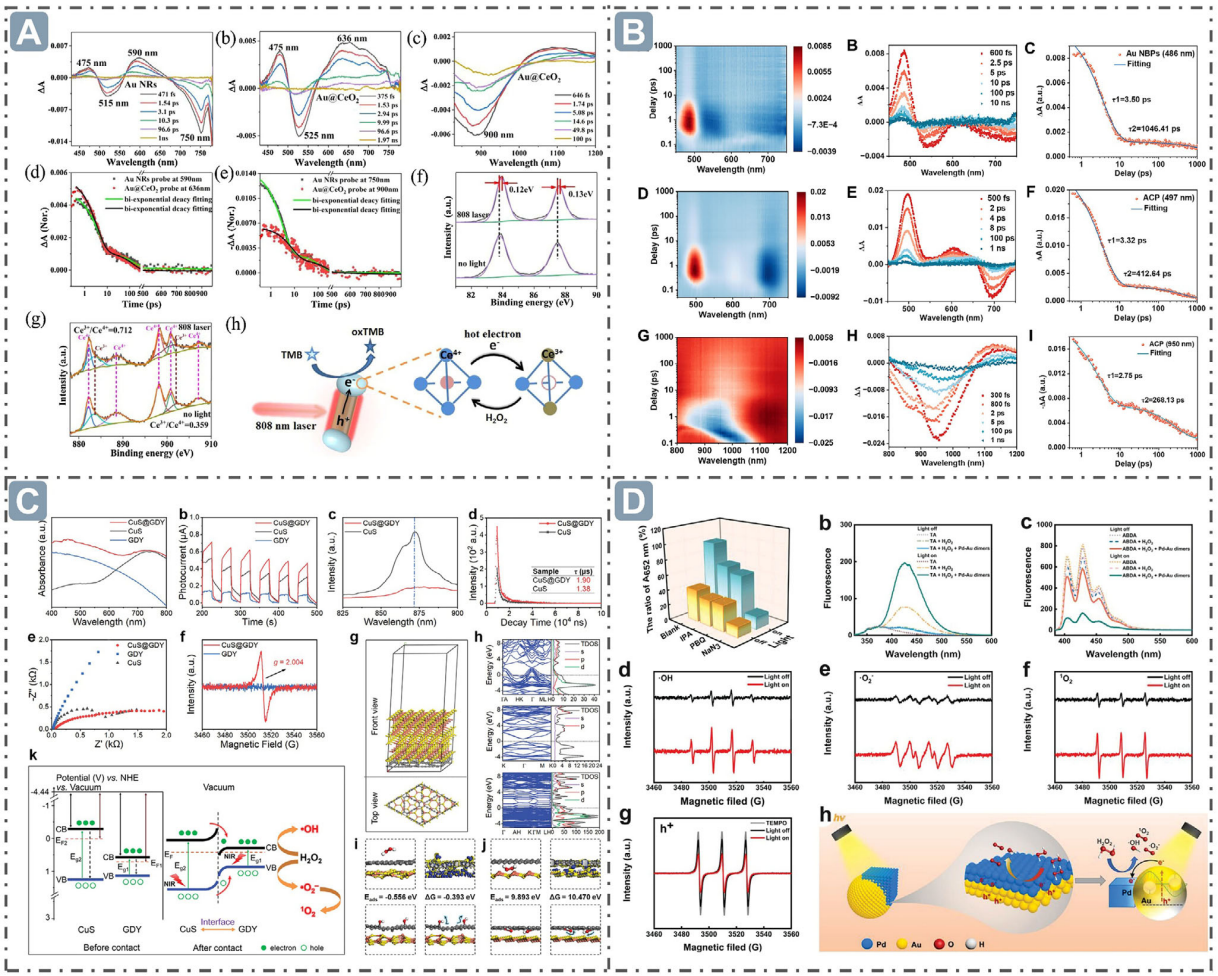
Photostimulation for boosting nanozyme catalysis. A) Photostimulation for boosting Au@CeO_2_ hybrid nanozyme catalysis. Reproduced with permission.^[^ ^136^ ^]^ *2021 Elsevier B.V. All rights reserved*. B) Femtosecond transient absorption spectroscopy measurements revealing the mechanism of photostimulation‐enhanced Au NBPs@Cu_2_O nanozyme catalysis. Reproduced with permission.^[^ ^137^ ^]^ *2023 Acta Materialia Inc. Published by Elsevier Ltd. All rights reserved*. C) Mechanism of NIR light for boosting peroxidase‐like activity of CuS@GDY. Reproduced with permission.^[^ ^138^ ^]^ *2022 Wiley‐VCH GmbH*. D) Mechanism of plasmon‐enhanced Pd‐Au dimers nanozyme catalysis. Reproduced with permission.^[^ ^139^ ^]^ *2023 Wiley‐VCH GmbH*.

With the rapid development of nanotechnology, the interaction between light and nanozyme has emerged as a research hotspot, with its scientific principles and application potential being striking.^[^ ^128^ ^]^ Light acts as a magic “key” for nanozymes with special properties, unlocking a new “door” to enhance catalytic activity. Whether it is the local surface plasmon resonance (LSPR) induced by light irradiation of plasmonic nanozymes,^[^ ^129^, ^130^ ^]^ the hot electron‐hole pair generated by light excitation of nanozymes, or the photothermal effect generated by light irradiation, etc., they all play a key role in the catalytic process of nanozyme. These light‐related mechanisms are intricately intertwined, profoundly influencing the interaction between nanozyme and substrate, reaction pathway, and catalytic efficiency. They thus offer a crucial foundation and direction for our understanding and further development of high‐performance nanozymes. For nanomaterials with plasmonic characteristics, light irradiation can induce the LSPR phenomenon,^[^ ^131^ ^]^ significantly enhancing the electromagnetic field intensity on the surface of nanozymes.^[^ ^132^ ^]^ This near‐field enhancement effect can strengthen the interaction between nanozyme and substrate, increase the adsorption capacity and reaction activity of substrate on the surface of nanozyme, and thus enhance the catalytic activity of nanozyme.^[^ ^133^ ^]^ For example, AuNPs with glucose oxidase‐like activity and LSPR can significantly enhance their catalytic activity under visible light, through adsorbing and activating glucose molecules by oscillating the local electromagnetic field under visible light.^[^ ^134^ ^]^ When light irradiates nanozyme materials, electrons on the material's surface are excited to generate a hot electron‐hole pair with high energy and activity. enabling them to transfer to the active sites of nanozymes or reaction substrates, thereby facilitating the progress of catalytic reactions.^[^ ^135^ ^]^ Additionally, a dumbbell‐like Au@CeO_2_ hybrid nanozyme with strong LSPR effect in the near‐infrared (NIR) window can generate the plasmon‐induced hot carriers due to its spatially separated nanostructure, thereby effectively enhancing its POD‐like activity under 808 nm irradiation. CeO_2_, as an n‐type semiconductor, can form a superior Schottky barrier when integrated with Au, which improves the hot electron injection. The hot electrons injected into CeO_2_ can reduce Ce^4+^ to Ce^3+^ and form O_v_. Oxygen‐deficient sites on nanozyme can increase the separation efficiency of photogenerated electron‐hole pairs and prevent electron–hole recombination.^[^ ^63^ ^]^ With the assistance of H_2_O_2_, the reduced Ce^3+^ is oxidized back to Ce^4+^, exhibiting POD‐like activity. The generated hot electron and H_2_O_2_ synergistically promote the electron transfer efficiency of Au@CeO_2_ hybrid nanozyme, thus increasing its catalytic activity (**Figure** **10**A).^[^ ^136^ ^]^ Meanwhile, Cu_2_O, as a p‐type semiconductor, can couple with gold nanobipyramid (Au NBPs) to form an appropriate Schottky barrier, enhancing the hot electron injection and thereby boosting nanozyme catalysis. Under 1064 nm laser irradiation, the interband transition of Au NBPs can be excited, generating electron‐hole pairs. The produced hot electrons can then be transferred into the conduction band (CB) of Cu_2_O to reduce Cu^2+^ and form O_v_. These generated O_v_ can efficiently promote the capture of photogenerated hot electrons and oxygen molecules, initiating the enhancement of OXD‐like activity and the formation of ROS (Figure 10B).^[^ ^137^ ^]^ Similarly, hollow copper sulfide nanocubes (CuS@GDY) wrapped in graphdiyne nanowalls (CuS@GDY) exhibit a strong LSPR effect under the NIR irradiation, enabling the generation of hot carriers. This significantly enhances the POD‐like activity of CuS@GDY under 808 nm NIR light. In the CuS@GDY nanostructure, CuS in the nanostructure serves to maximize interfacial area, promote plasmon–exciton coupling, and facilitate effective energy transport, while GDY, with its intrinsic narrow, functions to trap NIR light. The integration of CuS and GDY forms a suitable p‐p type heterojunction, which enhances the separation and injection of hot carrier. Additionally, carbon vacancies at the CuS and GDY interfaces act as reaction sites for H_2_O_2_, thus improving POD‐like activity (Figure 10C).^[^ ^138^ ^]^ Hot carriers (high‐energy electrons and holes) generated by light excitation can provide additional energy for the nanozyme‐catalyzed reactions. In some reactions, the presence of a high‐energy barrier limits the reaction rate. Photogenerated carriers can participate in forming the reaction's transition state and reduce the *E_a_* of the catalytic reaction, thus promoting the nanozyme catalysis. For example, under visible light irradiation, hot electron‐hole pairs generated on the Pd‐Au heteromeric NPs (Pd‐Au dimers) efficiently promote the decomposition of H_2_O_2_ to generate ROS, thus enhancing their POD‐like activity. Upon visible light irradiation, the calculated *E*_a_ of the developed nanozyme decreased from 47.02 to 32.77 kJ mol^−1^, indicating that hot carriers alter the catalytic reaction pathway and thus enhance POD‐like activity (Figure 10D).^[^ ^139^ ^]^ Besides, when light irradiates nanozymes, part of the light energy can be converted into thermal energy, which increases the local temperature around the nanozyme. This accelerates the movement of reaction molecules, thereby improving the reaction rate and substrate conversion efficiency. Meanwhile, elevated temperature may alter the structure and conformation of nanozymes, making them more conducive to binding to substrates and facilitating catalytic reactions. For example, Cu_2_MoS_4_ with NIR‐II light responsiveness exhibits a high photothermal conversion efficiency (37.8%) and excellent photothermal stability under NIR‐II light. This significantly enhances the nanozyme catalytic activity by increasing the local temperature of the reaction system according to the Arrhenius equation.^[^ ^140^ ^]^ Similarly, the catalytic activity of ultrasmall Ir nanocrystals (IrNCs) can also be boosted by mild photothermal heating under the NIR laser irradiation. Under 785 nm laser irradiation, the CAT‐like activity of IrNCs is promoted by this photothermal effect, accelerating the decomposition of H_2_O_2_ to generate O_2_.^[^ ^141^ ^]^

In conclusion, the action mechanism of light on nanozymes is multidimensional and interrelated, ranging from LSPR effect to hot carrier generation, from photothermal effect to the modulation of reaction energy barrier. These mechanisms synergistically enhance the catalytic activity of nanozymes and alter reaction pathways. These research findings not only deepen our understanding of nanozyme catalytic processes, but also provide abundant theoretical guidance for the design and application of novel, high‐efficiency nanozyme.

### 4.3 Electrical Stimulation

In the frontier research field of nanozymes, electrical stimulation plays a critical role in enhancing nanozyme activity, which is of great significance for advancing nanozyme‐related technologies and expanding their application scope. Electrical stimulation functions through multiple complex and subtle mechanisms: First, it modulates the chemical properties of nanozymes, including adjusting the electronic structure of active site and optimizing their reaction potentials^[^ ^142^ ^]^; Promoting the charge transfer between nanozymes and substrates to accelerate the reaction processes^[^ ^143^ ^]^; Altering reaction pathways to reduce the *E_a_* of the reactions while influencing substrate adsorption. Second, it triggers redox reactions on the nanozyme surface, generating ROS and simultaneously changing valence states of active sites.^[^ ^144^ ^]^ Additionally, for nanozymes with specific structures, electrical stimulation can accelerate electron movement, promote electron migration and energy conversion under the action of an electric field, and enhance electron transfer by means of atomic layer interfaces, thus significantly boosting nanozyme activity.^[^ ^145^ ^]^

**Figure 11:**
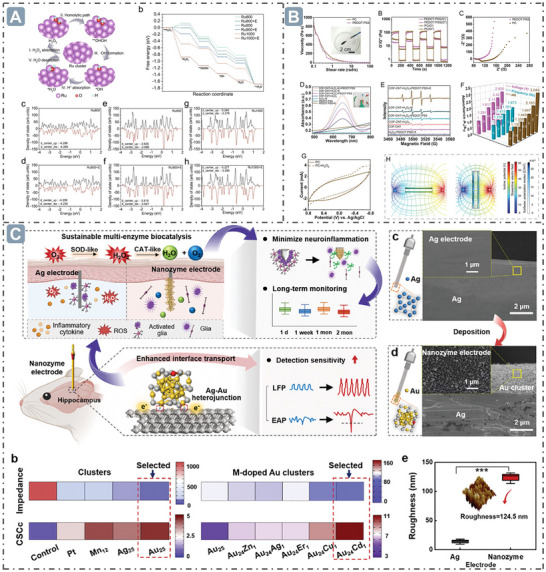
Electrical stimulation for boosting nanozyme catalysis. A) The mechanism of lattice expansion‐enhanced electro‐responsiveness for boosting nanozyme catalysis. Reproduced with permission.^[^ ^146^ ^]^ *Copyright 2024, The Author(s)*. B) Polarization effect under an electric field to boost nanozyme catalysis. Reproduced with permission.^[^ ^143^ ^]^ *2022 Wiley‐VCH GmbH*. C) The enhancement of quantum electronic transfers between the atomic layer interface for boosting nanozyme catalysis. Reproduced with permission.^[^ ^149^ ^]^ *2023 Wiley‐VCH GmbH*.

The principle by which electrical stimulation enhances nanozyme activity mainly involves the following aspects. On the one hand, electrical stimulation can modulate the electronic structure of the active sites of nanozymes, rendering them more prone to react with substrates and thereby increasing the catalytic activity. Simultaneously, it can also promote the charge transfer between nanozymes and substrates, accelerating the reaction rate. On the other hand, electrical stimulation can alter the reaction pathway of nanozyme‐catalyzed reactions, reducing the *Ea* of the reaction to improve the catalytic efficiency. Furthermore, it can affect the substrate adsorption capacity of nanozymes, enabling substrates to be more easily adsorbed and thus enhancing the catalytic activity. Benefiting from such electrical stimulation‐enhanced nanozyme catalysis, triboelectric nanogenerators, acting as self‐driven electric field systems, have attracted tremendous interest in biomedical fields.^[^ ^145^ ^]^ For example, Zhong et al. have studied the mechanism by which lattice expansion enhances electro‐responsiveness to boost nanozyme catalysis. Ru nanocrystals carbonized at 800, 900, and 1000 °C were designated as Ru800, Ru900, and Ru1000, respectively. DFT calculations of the conversion from H_2_O_2_ to •OH showed that the adsorption of H_2_O_2_ on Ru800 and Ru900 was a RSD, whereas it spontaneously adsorbs on Ru1000. The adsorption energy of the three samples decreased under an f electric field, with Ru1000 exhibiting a further reduction in reaction, indicating that lattice expansion and electrical stimulation reduce the electron density of Ru nanocrystals, thereby promoting their interaction with H_2_O_2_ and amplifying this effect. Comparison of the electronic density of states of Ru800, Ru900 and Ru1000, revealed that the *d*‐band center of Ru1000 was closer to the Fermi level than those of Ru800 and Ru900, with more electrons occupying states near the Fermi level. Upon electric field stimulation, the *d*‐band center of Ru1000 shifted even closer to the Fermi level, showing that lattice expansion and electric field stimulation promote the transfer of active electrons during catalysis. This was beneficial for catalyzing •OH formation, thus enhancing nanozyme catalysis (**Figure** **11**A).^[^ ^146^ ^]^ The electric field can induce the generation of plentiful ROS through triggering the redox reactions on the surface of certain nanozyme, which can also change the valence state of active sites in nanozyme, improving their catalytic activity. For example, under an electric field, PtMnIr nanozymes generate ROS via surface catalytic redox reactions. Their highly exposed active centers facilitate the electron/charge transfer under an alternating electric field. The local electric field elevates the valence state of highly active Mn^3+^ and Ir^3+^ in PtMnIr nanozymes, thereby accelerating their cascade enzymatic reactions alongside the electrodynamic effect. Besides, the high electronic conductivity and excellent catalytic activity of PtMnIr nanozymes endow them with superior electrodynamic performance, which is further enhanced by electric field stimulation to promote the electrons/charges transfer in the catalytic reaction.^[^ ^147^ ^]^ Additionally, nanozymes with high π‐electron delocalization and electron transport capacity can accelerate electron generation and migration of electrons via the polarization effect under an electric field, thereby enhancing their catalytic activity. For example, covalent organic framework (COF)‐carbon nanotube (CNT) (COF‐CNT) was fabricated by encapsulating CNT within 1D COF, and iron porphyrin (TAPP‐Fe) was the building block of COF, and Fe in the porphyrin ring served as the catalytically active center, showing POD‐like activity. Their 1D closed π‐conjugated structure, featuring high π‐electron delocalization and electron transport ability, provides an ideal platform for rapid migration and separation of electrons under an external electric field. Upon application of a 30 V voltage, 2D electrostatic simulations revealed increased polarization of COF‐CNT (acting as a dielectric), with this polarization unaffected by the orientation of COF‐CNT in the electric field. Surface charge polarization of COF‐CNT occurred with a magnitude of 23 Cm^−2^, which further accelerated electron generation and migration, enhancing their POD‐like activity (Figure 11B).^[^ ^143^ ^]^ Besides, hollow COF (hCOF) nanocages with a fully π‐conjugated nanostructure exhibit high electron mobility and low electron transfer resistance, and are more favorable for electron transfer near the Fe active centers, thereby enhancing their POD‐like catalysis. The surface energy density of hCOF was calculated by a 2D electrostatic simulation. In an ideal environment, application of 30V and 60V voltages led to obvious energy accumulation on the surface of fully π‐conjugated hCOF, which further promoted electron migration and electron‐mediated energy transfer. Besides, the hollow and fully conjugated structure of hCOF can improve the sensitivity of the nanocatalyst to external electric field and enhance energy conversion from the electric field, thus promoting catalytic reaction. Moreover, the hollow structure of hCOF can rearrange the local electric field, thus optimizing the utilization of electric field energy and enhancing its catalytic performance under the electric field.^[^ ^148^ ^]^ Enhanced quantum electron transfer across layer interfaces can greatly improve nanozyme catalytic activity. For example, nanozyme electrodes were prepared by depositing metal clusters (e.g., Au, Ag, Pt, Mn, etc.) onto silver electrode surfaces by the multi‐potential step method to form a functional layer. The atomic clusters on these nanozyme electrodes were uniformly distributed, and their surface roughness was much higher than that of Ag electrodes. This high roughness and large surface area exposed a large number of active catalytic sites, which were beneficial for improving the cathode charge storage capacity and reducing the impedance, thus enhancing nanozyme activity. Additionally, Au nanoclusters preferentially localized near stacking faults in Ag electrode substrates, attributed to strong Ag‐Au bonds. Ag dislocations at Au‐Ag junctions exposed high‐energy catalytic sites, forming new catalytically active sites and faster electron transfer pathways, thus enhancing the nanozyme activity through atomic‐level electrode interfaces (Figure 11C).^[^ ^149^ ^]^

Summarily, electrical stimulation enhances nanozyme activity through multi‐faceted mechanisms. From altering the electronic structure of nanozymes' intrinsic active site, modifying reaction pathway, regulating substrate adsorption ability, and promoting charge transfer, to inducing redox reaction to alter valence states, and exerting unique effects on nanozyme with special structures. These mechanisms are intertwined and jointly boosting nanozyme catalytic activity. This series of research results not only deepen our understanding of the principle underlying electrical stimulation‐mediated nanozyme activation nanozyme activation, but also lay a solid foundation for the design of next‐generation nanozymes.

### 4.4 Magnetic Field Stimulation

As a unique external stimulus, the magnetic field shows significant potential in regulating the catalytic activity of nanozyme.^[^ ^150^ ^]^ Numerous studies have demonstrated that magnetic fields can affect the activity of nanozyme through multiple mechanisms, among which the magnetothermal effect and electron transfer effect are particularly critical. The magnetothermal effect originates from hysteresis loss,^[^ ^151^ ^]^ altering the local temperature environment around nanozymes and thereby influencing their catalytic performance. The electron transfer effect involves spin polarization and vortex Lorentz electric field,^[^ ^152^ ^]^ modulating the electron transfer process within nanozymes, and ultimately enabling the regulation of catalytic activity.

**Figure 12:**
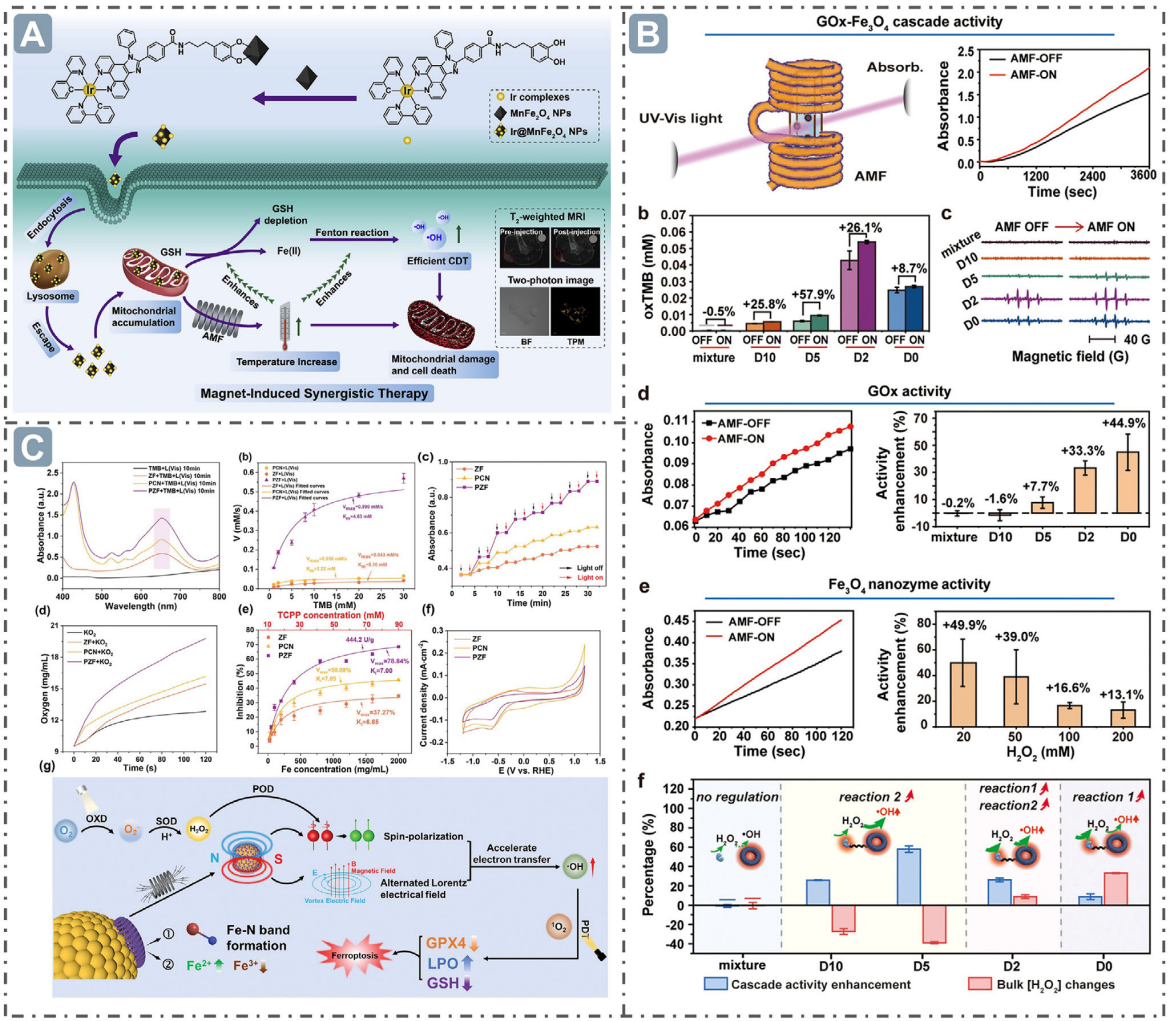
Magnetic field stimulation for boosting nanozyme catalysis. A) The magnetocaloric effect for the boosting POD‐like catalysis of Fe_3_O_4_ nanozymes. Reproduced with permission.^[^ ^155^ ^]^ *2020 Elsevier Ltd. All rights reserved*. B) Boosting catalytic activities of four kinds of Fe_3_O_4_ nanoring@GOx nanozymes with different connection distances through alternating magnetic field. Reproduced with permission.^[^ ^156^ ^]^ *Copyright 2021 American Chemical Society*. C) Magnetic field‐enhanced the electron transfer efficiency for boosting nanozyme catalysis. Reproduced with permission.^[^ ^157^ ^]^ *2024 Wiley‐VCH GmbH*.

The magnetothermal effect, generated from hysteresis loss, can increase the local temperature of nanozymes to improve their catalytic activity, with the specific absorption rate used to evaluate the magnetothermal conversion efficiency. For example, as the amplitude of the alternating magnetic field increases, the POD‐like catalytic activity of iron oxide NPs is significantly enhanced, while the bulk solution temperature remains unchanged during the measurement. This demonstrates that the enhancement of nanozyme catalysis is attributed to the localized heating of the surface‐active sites of Fe_3_O_4_ NPs by the alternating magnetic field, and the corresponding nanozyme kinetics also conforms to Michaelis‐Menten. The magnetothermal enhancement of nanozyme catalytic performance has also been shown to depend on the field strength and the specific absorption rate of iron oxide NPs.^[^ ^153^ ^]^ Besides, the magnetothermal effect induced by the magnetic field can raise the local temperature to a mild temperature of 42 °C, which promotes the diffusion of nanozymes and increases the POD‐like catalytic activity of Fe_3_O_4_ nanozyme.^[^ ^154^ ^]^ Moreover, the local temperature of Ir@MnFe_2_O_4_ NPs under alternating magnetic field can be elevated and accelerate the transformation of Fe^3+^ to Fe^2+^ owing to the local magnetic heat, which boosts nanozyme catalytic activity and increases •OH generation (**Figure** **12**A).^[^ ^155^ ^]^ Besides, an alternating magnetic field can also boost the catalytic activity of both nanozymes and enzymes through magnetothermal effect. For example, four kinds of Fe_3_O_4_ nanoring@GOx nanozymes with different connection distances were prepared by functionalizing with polyethylene glycol (PEG) spacers with different molecular weights, followed by covalent anchoring of GOx onto the Fe_3_O_4_ nanoring. Adjusting the molecular weight of the PEG spacers regulated the distance between GOx and Fe_3_O_4_ nanoring surface. Fe_3_O_4_ nanoring with high saturation magnetization and large hysteresis area exhibited strong heat conversion ability under alternating magnetic field. The heat generated by Fe_3_O_4_ nanoring forms a thermal gradient around the nanozyme, affecting the activity of nearby enzymes, and the enhancement degree exhibits an inverse relationship with distance from Fe_3_O_4_ nanoring. Alternating magnetic fields also enhance the catalytic activity of Fe_3_O_4_ nanoring through the magnetothermal effect (Figure 12B).^[^ ^156^ ^]^ Additionally, magnetic fields can promote the electron transfer efficiency, thereby enhancing nanozyme catalytic activity, primarily involving spin polarization effects and vortex Lorentz electric field effects. For example, an alternating magnetic field of relatively low intensity to avoid magnetothermal effect was used to investigate the mechanism of magnetic dipole interactions‐enhanced nanozyme catalysis. Under this alternating magnetic field, strong electron spin polarization occurred in the MOF‐based magnetic nanozyme (PZFH), leading to well‐arranged spin configurations in contrast to the irregular counterpart. This facilitates electron transfer and further improves the ROS yield. Furthermore, the alternating magnetic field generates a vortex Lorentz electric field around PZFH, and accelerates interface charge transfer and diffusion between nanozymes and substrates, thereby enhancing the production of ROS (Figure 12C).^[^ ^157^ ^]^

To sum up, remarkable progress has been made in the study of the magnetic field in regulating the catalytic activity of nanozyme. Through magnetothermal effects, such as increasing local temperature based on hysteresis loss, spin polarization, and vortex Lorentz electric field in electron transfer effect, many ways are provided for precise regulation of nanozyme catalytic activity. These research results not only deepen our understanding of the interaction mechanism between magnetic field and nanozyme, but also expand new possibilities for the application of nanozyme in more fields through magnetic field regulating nanozyme activity.

### 4.5 X‐ray Stimulation

X‐ray plays a crucial role in enhancing the catalytic activity of nanozymes. Unlike ultraviolet radiation, X‐ray exerts its influence on nanozymes by virtue of its high‐energy radiation features, which are readily and effectively absorbed by the nanozymes.^[^ ^158^ ^]^ By exciting electrons within the nanozymes, X‐ray induces alterations in the valence state of their active sites. Moreover, the frequency of these valence state changes is enhanced, thereby exerting a positive and profound impact on the catalytic activity of the nanozymes.^[^ ^159^ ^]^ This process encompasses multiple mechanisms, including the variations in the activity of diverse types of nanozymes, as well as the functions in electron transfer, ROS generation, and photo‐generated electron‐hole pair separation processes.

**Figure 13:**
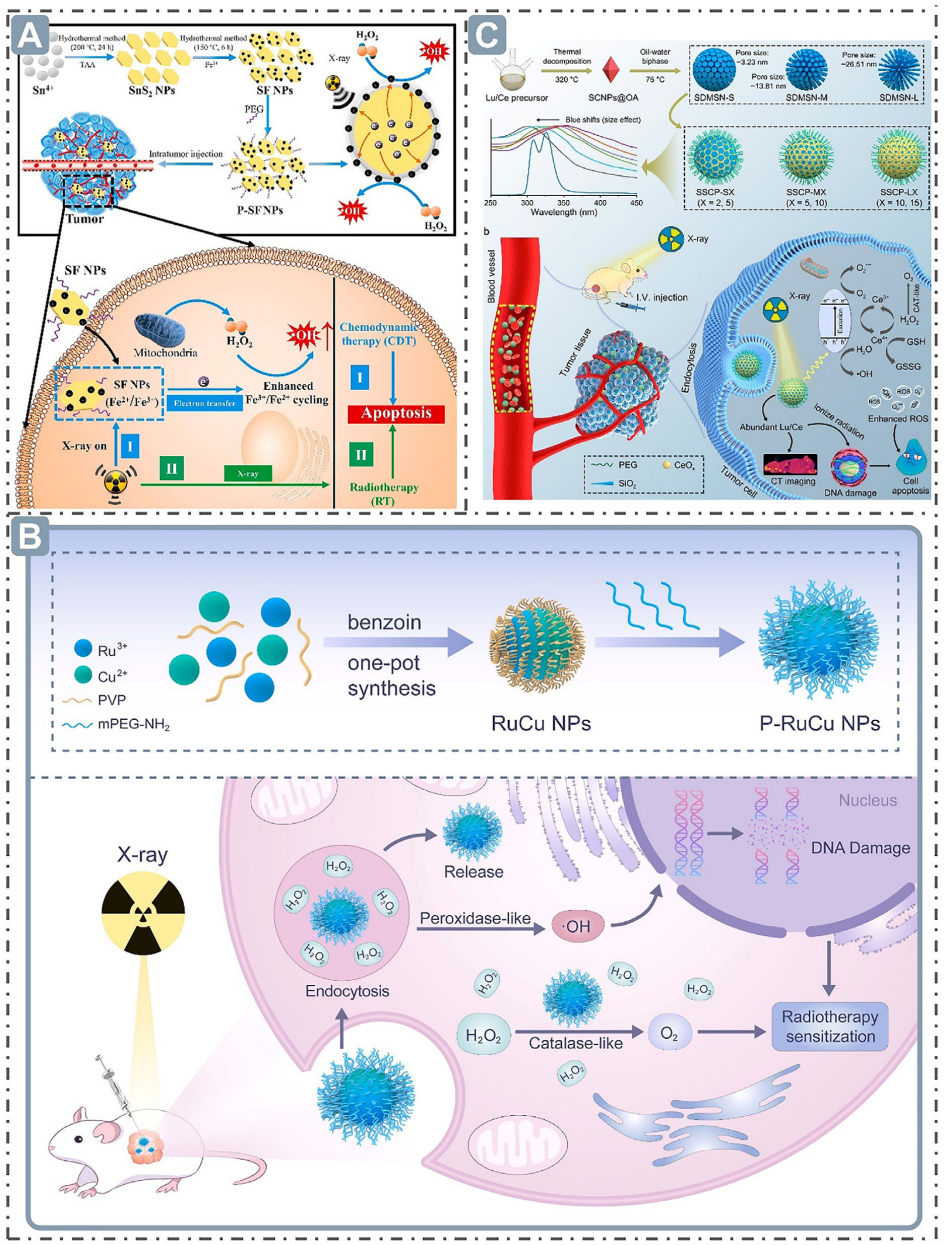
X‐ray stimulation for boosting nanozyme catalysis. A) X‐ray excitation‐accelerated electron transfer efficiency for boosting nanozyme catalysis. Reproduced with permission.^[^ ^161^ ^]^ *2021 Elsevier Ltd. All rights reserved*. B) X‐ray irradiation for boosting RuCu nanozyme catalysis through accelerating the electron transfer between the nanozyme and substrates. Reproduced with permission.^[^ ^163^ ^]^ *2022 Elsevier Ltd. All rights reserved*. C) X‐ray irradiation promoted the separation of the photoinduced electron–hole pair for boosting nanozyme catalysis. Reproduced with permission.^[^ ^164^ ^]^ *Copyright 2022 American Chemical Society*.

Compared with ultraviolet irradiation, X‐ray, with higher‐energy radiation, can be absorbed by nanozymes and excite their electron electrons, thereby accelerating the valence state transformation frequency of active sites in nanozymes and enhancing their catalytic activity. For example, X‐ray stimulation can accelerate the transformation frequency Fe^2+^/Fe^3+^ in FeN_4_ SAzymes due to its high‐energy radiation, which promotes the enhancement of self‐cascade enzyme‐like activity. At pH 6.49, this increases the POD‐like activity from 52.69 to 60.51 U mg^−1^ and the GSH OXD (GSHOx)‐like activity from 1.18 to 1.57 U mg^−1^. This self‐cascade enzyme‐like activity arises because X‐ray stimulation enhances FeN_4_ SAzymes, enabling them to efficiently catalyze H_2_O_2_ into •OH. Simultaneously, Fe^2+^ at the active site is transformed into Fe^3+^, which participates in subsequent GSHOx‐like reactions to consume GSH, thereby preventing GSH from scavenging ROS.^[^ ^160^ ^]^ The electron transfer efficiency of nanozyme can be enhanced after X‐ray excitation. For example, Zhang et al. proposed a method to accelerate the redox cycle of SnS_2_@Fe_3_O_4_ NPs nanozymes, consisting of SNS_2_ NSs and Fe_3_O_4_ quantum dots with variable/mixed redox states, under X‐ray irradiation, thereby enhancing their nanozyme activity. Under external X‐ray irradiation, SnS_2_, as an electron donor, can be triggered to transfer electrons to Fe_3_O_4_ quantum dots because of the matching electronic band structure. This electron transfer accelerates the reduction of Fe^3+^ to Fe^2+^ on the Fe_3_O_4_ surface, promotes the regeneration of Fe^2+^ active sites, and thus maintains the high catalytic activity of Fe_3_O_4_. The regenerated Fe^2+^ sites can react with H_2_O_2_ to continuously produce more ROS, such as •OH (**Figure** **13**A).^[^ ^161^ ^]^ The outer‐layer electrons in nanozymes can be excited to form photoelectrons via the photoelectric effect by X‐ray irradiation, which participate in nanozyme catalysis. For example, DOX@hollow mesoporous silica NPs/Mn_3_O_4_ (DOX@HMSN/Mn3O4(R)) nanozyme can combine with X‐ray radiation to effectively promote photoelectron generation to participate in their OXD‐like catalysis, thereby enhancing their catalytic activity.^[^ ^162^ ^]^ Moreover, the mechanism by which X‐ray radiation enhances nanozyme catalysis involves accelerating ROS generation and electron transfer between the nanozyme and substrates. For example, RuCu nanozyme, containing high atomic number, can efficiently absorb the radiation energy of X‐rays, exciting the inner‐shell electrons to generate photoelectrons and Auger electrons. This electron excitation and transition process provides an additional energy source for subsequent chemical reactions, potentially enhancing nanozyme catalytic activity. These high‐energy photoelectrons and Auger electrons migrate through the crystal structure of the nanozyme and interact with active sites. For RuCu NPs with POD‐like and CAT‐like activities, these electrons alter the electronic structure and charge distribution of active sites, facilitating interactions with substrate molecules (such as H_2_O_2_) and promoting the catalytic reaction. In POD‐like catalytic processes, H_2_O_2_ is decomposed by nanozyme, and the electron transfer process is enhanced by X‐ray accelerates this reaction and increases the production rate of •OH. Additionally, CAT‐like activity involves decomposing H_2_O_2_ into O_2_ and H_2_O, a process also influenced by X‐ray irradiation. This further regulates ROS balance and enhances tumor cell killing, indirectly confirming enhanced nanozyme catalytic activity (Figure 13B).^[^ ^163^ ^]^ X‐ray irradiation can promote the separation of the photoinduced electron–hole pair to enhance nanozyme catalysis. For example, scintillating NPs (LiLuF_4_:2%Ce nanoscintillator) can convert high‐energy X‐ray into emitted light in ultraviolet‐visible light (308 nm and 326nm). When X‐ray interacts with the Lu atom in LiUF_4_, plentiful secondary electrons and Auger electrons are ejected on the surface of scintillating NPs through Compton scattering and photoelectric effect. Ce^3+^ in the ground state can be excited by absorbing the energy of these electrons and transitions, and then emits the ultraviolet light because the excited state of Ce^3+^ returns to the energy levels of ^2^F_5/2_ and ^2^F_7/2_ in 4f^1^ configuration. This emitted ultraviolet light induces the separation of electron‐hole pair by driving the transition of photogenerated electrons in CeO_x_ from the valence band to the conduction band, which then triggers the ROS generation through the transfer of electron‐hole pairs on CeO_x_ surface (Figure 13C).^[^ ^164^ ^]^

To sum up, the enhancement of nanozyme catalytic activity by X‐ray is a complex process involving multiple mechanisms and diverse nanozyme types. From changing the valence state of active sites to affecting electron transfer, ROS generation, and photogenerated electron‐hole pair separation, numerous research examples clearly show the unique significance of X‐ray in the field of nanozyme catalysis. These research advances not only deepen our understanding of nanozyme catalytic mechanisms, but also lay the groundwork for future efforts to utilize X‐rays in regulating nanozyme performance, enabling more efficient catalytic applications.

### 4.6 Thermal Stimulation

Increasing temperature intensifies the thermal movement of substrate molecules, and accelerates their movement in solution and thereby increasing the collision frequency with the active site of nanozymes.^[^ ^117^, ^165^ ^]^ More frequent collisions mean substrate molecules are more likely to bind to nanozymes and undergo reactions, thus accelerating the catalytic reaction rate. According to Arrhenius formula (where is the reaction rate constant, which refers to the pre‐factor, *Ea*, the gas constant, and the temperature), the reaction rate constant increases with rising temperature (Equation (6)).^[^ ^166^ ^]^ Recently, photothermal conversion and magnetothermal effect, raising the temperature around nanozymes, have been widely used as efficient thermal stimulation methods to boost nanozyme catalysis. Notably, cold stimulation can also boost the catalytic activity of certain specific nanozymes through their unique mechanisms.

(6) $$k=Ae^{\frac{-E_{a}}{RT}}$$

**Figure 14:**
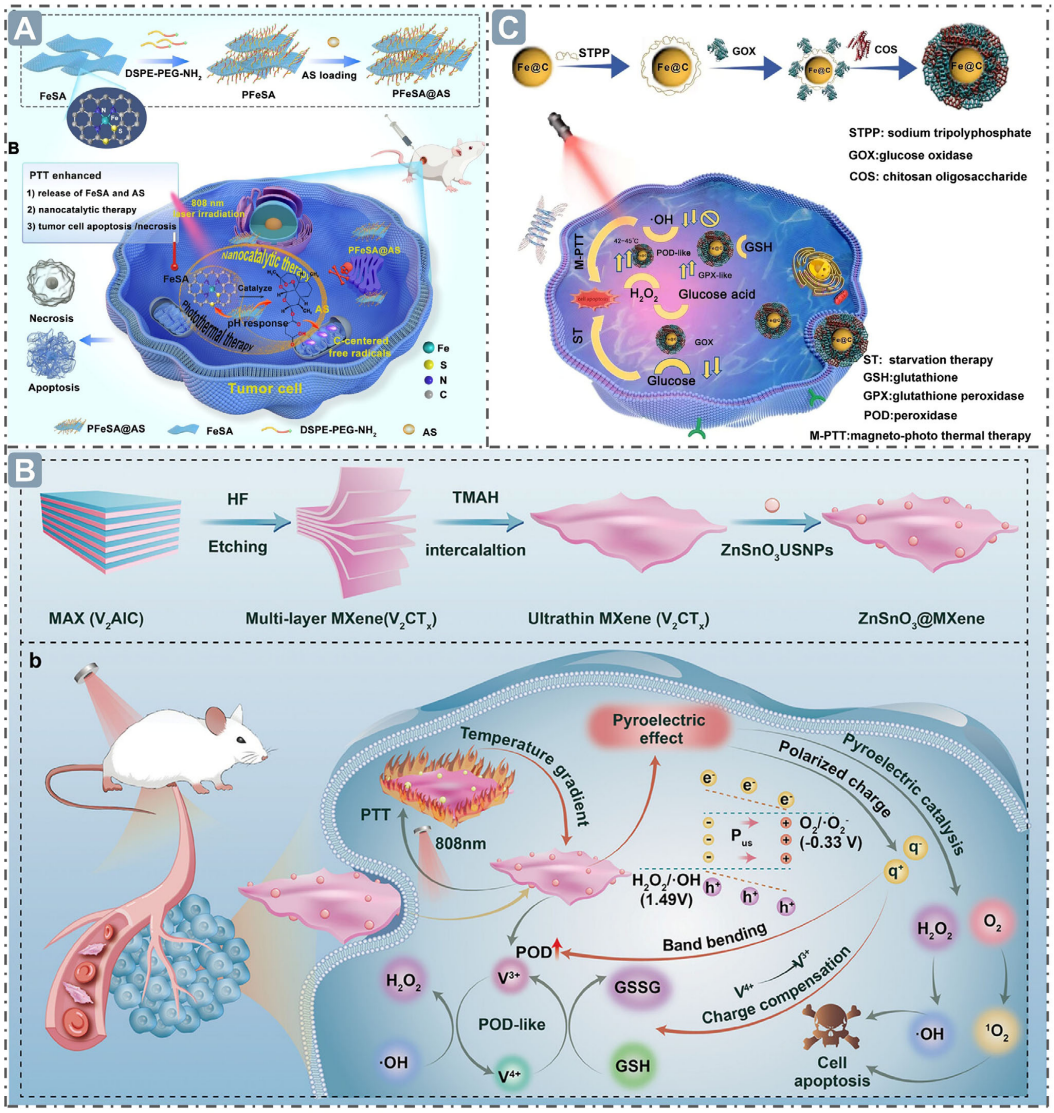
Thermal stimulation for boosting nanozyme catalysis. A) Fe‐SAzymes with obvious light absorption in NIR region and good photothermal conversion property for boosting nanozyme catalysis. Reproduced with permission.^[^ ^168^ ^]^ *2022 Published by Elsevier Ltd on behalf of Acta Materialia Inc*. B) ZnSnO_3_@MXene with good NIR light absorption for boosting POD‐like catalysis. Reproduced with permission.^[^ ^170^ ^]^ *2024 Wiley‐VCH GmbH*. C. Magnetocaloric effect iron carbon NPs/GOx/chitosan oligosaccharide for boosting nanozyme catalysis. Reproduced with permission.^[^ ^172^ ^]^ *2024 Elsevier B.V. All rights are reserved, including those for text and data mining, AI training, and similar technologies*.

Nanozymes with photothermal properties can enhance their catalytic activity through photothermal conversion, which elevates the local reaction temperature. For example, the *V*_max_ and *K*_m_ of Fe‐SAzymes with POD‐like activity increased from 4.05 × 10^−7^ to 7.13 × 10^−7^ M s^−1^ and decreased from 4.73 to 1.49 mM after 808 nm laser irradiation, respectively. This demonstrates that thermal stimulation can boost the POD‐like catalysis of Fe‐SAzymes.^[^ ^167^ ^]^ Similarly, Fe‐SAzymes exhibit significant light absorption in the NIR region and excellent photothermal conversion characteristics, which can promote their catalytic reaction (**Figure** **14**A).^[^ ^168^ ^]^ Modification with polydopamine (PDA) to enhance the NIR light absorption ability can improve the photothermal performance of nanozymes to boost their catalysis. In PDA@C3N4@MIL/glucose oxidase (PCMG) composites, PDA exhibits excellent NIR light absorption ability, and the temperature of PCMG increases with the increasing PCMG concentration under the irradiation of 808nm laser irradiation. The catalytic activity of PCMG can be enhanced via its photothermal conversion, which elevates the catalytic temperature, a finding supported by evaluating the catalytic ability at the same reaction temperature. The •OH level at 50 °C was equivalent to that of 200 µg mL^−1^ PCMG under 808 nm laser irradiation, indicating that the enhancement of catalytic reaction by photothermal stimulation is primarily due to increased temperature.^[^ ^169^ ^]^ Moreover, the POD‐like activity of ZnSnO_3_@MXene, with good NIR light absorption, can be enhanced via photothermal conversion under 808 nm laser irradiation, as evidenced by increased •OH concentration under both heating and 808 nm laser irradiation (Figure 14B).^[^ ^170^ ^]^ Besides, under an alternating magnetic field, magnetic nanomaterials can generate heat via hysteresis loss. This localized thermal effect raises the temperature around nanozymes, accelerating the thermal motion of substrate molecules, increasing the collision frequency and energy between substrate molecules and active sites of the nanozyme, and thus facilitating the reaction to enhance the catalytic activity. For example, the catalytic activity of Fe_2_C@Fe_3_O_4_‐PEG nanozymes, endowed with highly magnetothermal performance, can be boosted under an alternating magnetic field. Due to their higher magnetic energy product, yolk–shell‐like Fe_2_C@Fe_3_O_4_‐PEG nanozymes exhibit better magnetothermal performance than Fe_2_C or Fe_3_O_4_, thereby further improving nanozyme catalytic activity under alternating magnetic field.^[^ ^171^ ^]^ At magnetothermal temperatures, the catalytic performance of iron carbon NPs/GOx/chitosan oligosaccharide nanozyme can be enhanced by increasing local temperature and promoting ROS generation (Figure 14C).^[^ ^172^ ^]^ Additionally, cold can also activate the catalytic activity of certain nanozymes. For example, cold temperature could activate the GSH OXD‐like (GSHOx)‐like activity of Bi_2_Fe_4_O_9_ NSs owing to their pyroelectricity. Finite element simulation revealed that Bi_2_Fe_4_O_9_ NSs exhibit a pyroelectric potential of 3.1 eV under cold conditions. Thermodynamic calculations showed that the adsorption energy of GSH on Bi_2_Fe_4_O_9_ NSs was −4.15 eV, indicating good catalytic selectivity for their GSHOx‐like activity. Notably, this GSHOx‐like activity is only observed under cold temperature fluctuations and vanishes at constant temperatures, with its enzymatic kinetics strongly correlated to the frequency of temperature variation.^[^ ^173^ ^]^

In conclusion, increasing temperature intensifies the thermal movement of substrate molecules, accelerates their movement in solution, raises the frequency of collisions with the active site of nanozyme, thereby enhancing the catalytic reaction rate. Photothermal conversion and magnetothermal effect, which elevate the temperature around the nanozyme, serve as effective thermal stimulation methods to boost nanozyme catalysis. Notably, certain nanozymes can also be activated by cold temperature stimulation. These studies not only deepen our understanding of the catalytic mechanism of nanozyme from the thermal perspective but also provide an important way for the design and development of thermally stimulated nanozymes in highly catalytic applications.

## 5 Machine Learning‐Assisted Design for Boosting Nanozyme Catalysis

The conventional rational design of nanozymes with high catalytic activities has long been constrained by the empirical “trial‐and‐error” approaches, which struggle to systematically decode the intricate relationships between material properties and catalytic performance.^[^ ^174^ ^]^ Recent advancements in ML have revolutionized this field by enabling data‐driven exploration of structure‐activity relationships across multiple scales.^[^ ^175^, ^176^, ^177^ ^]^ Compared with conventional methods, ML algorithms can rapidly analyze massive datasets encompassing materials, activity, and other parameters to predict potential high‐activity nanozymes and narrow down the candidate range, thus greatly improving efficiency and get rid of the plentiful “trial‐and‐error” experiments. More importantly, ML algorithms can achieve rational design and break the bottleneck of empirical dependence, which can reveal the hidden laws between material parameters and catalytic activity through establishing a quantitative structure‐activity relationship mode. The features of nanozymes can also be visualized by interpretable ML analysis and achieve the “on‐demand design”. Crucially, the traditional “performance trade‐off” problem that it is difficult to give consideration to catalytic activity and feasibility, can be effectively solved by ML algorithms. Therefore, ML significantly reduces the experimental costs and resource consumption of traditional high‐throughput screening of nanozyme development. Summarily, from constructing standardized databases that merge experimental characterization with theoretical insights to developing predictive models for catalytic activity and functional types, ML technology provides a transformative framework for accelerating nanozyme design. By bridging computational screening, experimental validation, and mechanism revelation, ML‐driven approaches not only enhance catalytic efficiency but also enable the customization of nanozymes for complex applications, highlighting the synergy between ML and nanozyme engineering, underscoring its potential to unlock next‐generation nanozymes with tailored functionalities.

### 5.1 Feature Extraction of Nanozymes and Database Construction

**Figure 15:**
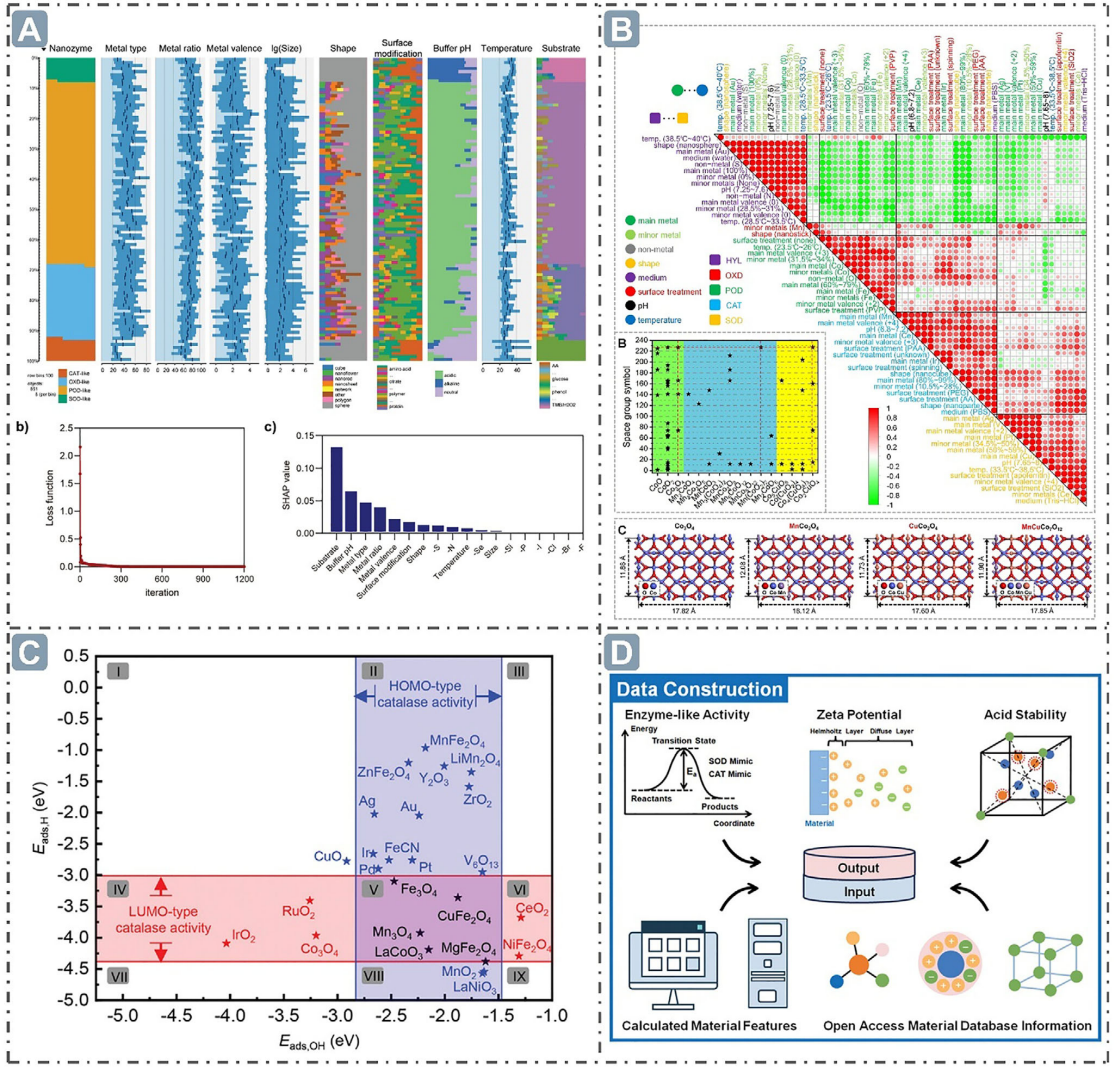
Features extraction of nanozyme and prediction databases construction. A) The construction of a database from experimental results in literature. Reproduced with permission.^[^ ^20^ ^]^ *2022 Wiley‐VCH GmbH*. B) The cluster analysis of the nanozyme features from experimental results in literatures. Reproduced with permission.^[^ ^179^ ^]^ *Copyright 2024 American Chemical Society*. C) *E_a_*‐based descriptor for the prediction of CAT‐like nanozymes. Reproduced with permission.^[^ ^180^ ^]^ *2023 Wiley‐VCH GmbH*. D) Database construction based on the application requirements. Reproduced with permission.^[^ ^181^ ^]^ *2025 Wiley‐VCH GmbH*.

Data‐driven modeling and feature engineering are the core sections of ML‐assisted nanozyme design, with their core lies in the integration of multi‐source data. The material information of nanozymes includes numerous indexes, such as morphology, elemental composition, crystal structure, particle size, and surface modification, which can purposefully manifest in the catalytic performance, such as catalytic activity, substrate affinity, and catalytic types.^[^ ^10^, ^178^ ^]^ As discussed in the context of the relationship between electronic structure and nanozyme catalysis, electronic information of nanozymes derived from theoretical calculations, containing *d*‐band center, *e_g_* occupancy, and electronic spin, can dramatically affect the indexes of reaction process, such as *E_ads_*, free energy, thus regulating the catalytic performance‐related data of nanozymes. Therefore, a standardized database can be built by integrating these multi‐source data, including material information from experiments and structure and reaction information from theoretical calculations. The key features of nanozymes can be drawn from the standardized database to establish a high‐precision prediction model, enabling the rational design of high‐performance nanozyme. For example, a standardized database was built by 920 pieces of data through considering the internal factors, such as size, shape, metal‐containing number, metal type, metal valence, nonmetal element, surface modification, and catalytic type, and external factors, including pH, temperature, and substrate, which were extracted from over 300 papers (**Figure** **15**A).^[^ ^20^ ^]^ Besides, 1000 kinds of nanozyme material information, containing element type, element proportion, chemical valence state, shape, pH value, etc., were collected from 316 relevant documents selected from 4159 papers to construct a standardized database (Figure 15B).^[^ ^179^ ^]^ In addition to the experimental database, data information from theoretical calculations constituted another pathway approach to building nanozyme databases. For example, *E_a_* was predicted by ML through the existing material structural features, such as lattice parameters and electronic structure, in the Computational 2D Materials Database (C2DB). These predicted *E_a_* values were used as new data to enrich the C2DB database (Figure 15C).^[^ ^180^ ^]^ The database can also be customized based on application requirements to develop high‐performance nanozymes. For example, the potential elemental combinations and electronic features, such as orbital energy levels and electron density, were calculated by first‐principles calculations and wavefunction analysis, respectively. The fabricated database was linked to ulcerative colitis treatment‐related indexes, such as SOD‐, CAT‐like activities, zeta potential, and acid stability, thus establishing structure–activity relationships between nanozyme structural parameters and treatment requirements (Figure 15D).^[^ ^181^ ^]^ In summary, extracting features from nanozyme information for database construction lays the foundation for establishing accurate models to predict high‐performance nanozymes. The integration of experimental and theoretical calculation data not only deepens our understanding of the structure‐catalytic relationship in nanozymes but also aligns closely with practical application needs, providing key insights into the structure‐activity relationship for addressing complex biomedical challenges.

### 5.2 Prediction of Nanozyme Catalytic Activity

**Figure 16:**
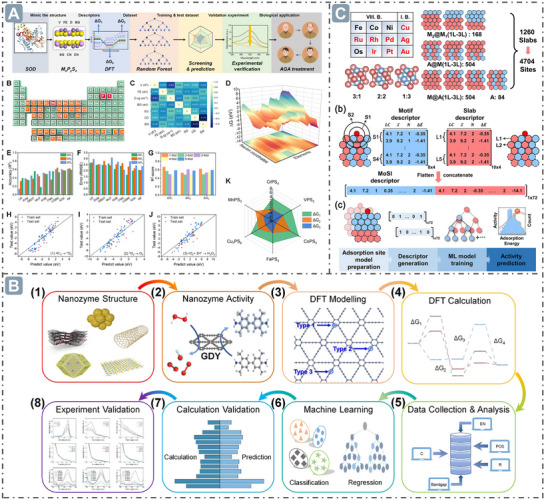
ML models for the prediction of nanozyme catalytic activity. A) ML models for the prediction of SOD‐like activity of *MnPS*_3_. Reproduced with permission.^[^ ^182^ ^]^ *Copyright 2022 American Chemical Society*. B) ML frameworks for the catalytic activity of GYD‐based nanozymes. Reproduced with permission.^[^ ^183^ ^]^ *Copyright 2022 American Chemical Society*. C. CatBoost algorithm based on “Mo‐Sl” descriptor for the prediction of Bimetallic nanozymes with POD‐like activity. Reproduced with permission.^[^ ^184^ ^]^ *Copyright 2024 American Chemical Society*.

ML technology has demonstrated remarkable advantages in predicting nanozyme activity and revealing the structure‐activity relationship. Based on multi‐scale features of nanozymes, such as material composition, electronic structure and geometric parameters, a nanozyme catalytic activity prediction model can be constructed by establishing a quantitative correlation between the database and catalytic activity. Regression models are often used to bridge the database and nanozyme catalytic activity, thereby enabling nanozyme catalytic activity prediction. For example, a stretch of ML regression models, containing support vector machine (SVM), logistic regression (LR), eXtreme gradient boosting (XGBoost), gradient boosting decision tree (GBDT), convolutional neural network (CNN), multilayer perceptron (MLP), adaptive boosting (ADA), light gradient boosting machine (LGBM), and random forest (RF), were used to test the *M_x​_P_y_​S_z_* ​ database. After optimizing hyperparameters via statistical Bayesian optimization, RF model outperformed others in terms of decision coefficient (R^2^) and root‐mean‐square error (RMSE). It accurately predicted that *MnPS*_3_ exhibits the highest SOD‐like activity, which is consistent with experimental results (**Figure** **16**A).^[^ ^182^ ^]^ Additionally, 168 non‐metal atom‐doped graphdiyne models were fabricated to develop the database, containing electronegativity, doped atomic radius, doped position, concentration, and bandgap of doped atoms. After determining hyperparameters via Bayesian optimization, LR, SVM, GBDT, k‐nearest neighbor (KNN), RF, decision tree (DT), LGBT, and XGBoost models were used to train and test the database. XGBoost model showed the best performance, with accuracy of 65.8%, in the classification of the maximum energy‐consuming step in aspects of accuracy, F1 score, AUC, and mean absolute error (MAE), mean square error (MSE), and R^2^. Moreover, it showed low sensitivity to outliers and no overfitting. The predicted reaction steps were consistent with the actual steps, confirming the effectiveness of the correlation‐based feature selection strategy and the accuracy of ML model predictions. In predicting the maximum energy barrier, XGBoost model also performed well, with R^2^ = 0.781, MAE = 6.76, MSE = 172.18, and the feature importance and Shapley Additive exPlanations (SHAP) analysis were consistent with those of the classification model. The predicted trend aligned with DFT calculation results, verifying the feasibility of ML model in nanozyme catalytic prediction (Figure 16B).^[^ ^183^ ^]^ Additionally, the volcanic type correlation between POD‐like activity and O* adsorption energy of pure mental nanozyme laid the foundation for constructing a subsequent ML model, which can simplify complex reaction calculations and reduce the prediction dimension. Based on this relationship, a new descriptor named “Mo‐Sl” was proposed by extracting key attributes from adsorption sites and substrate materials. This descriptor better reflects the chemical environment information, thereby improving the prediction accuracy. An efficient prediction model was constructed by integrating the CatBoost algorithm, selected via the automatic ML package PyCaret. After processing and analyzing a large amount of data, the developed ML model screened bimetallic nanozymes with high POD‐like activity, w showing high consistency with experimental results and thus demonstrating the effectiveness of the Mo‐Sl descriptor. Specifically, alloys containing elements, such as Au, Ag, Pd, and Pt, generally exhibited high POD‐like activity in nanozymes, whereas those containing more elements, such as Cu, Rh, Ir, and Ru, showed low activity. Additionally, feature importance analysis of the descriptor revealed that lattice constant played a key role in influencing surface O* adsorption energy, whereas electronegativity had a relatively minor impact (Figure 16C).^[^ ^184^ ^]^ In conclusion, not only ML model demonstrate the transformative power in deciphering the complex catalytic mechanisms of nanozymes, but they also brings great hope for accelerating the discovery of the next generation of nanozymes with customized catalytic characteristics.

### 5.3 Prediction of Nanozyme Types

**Figure 17:**
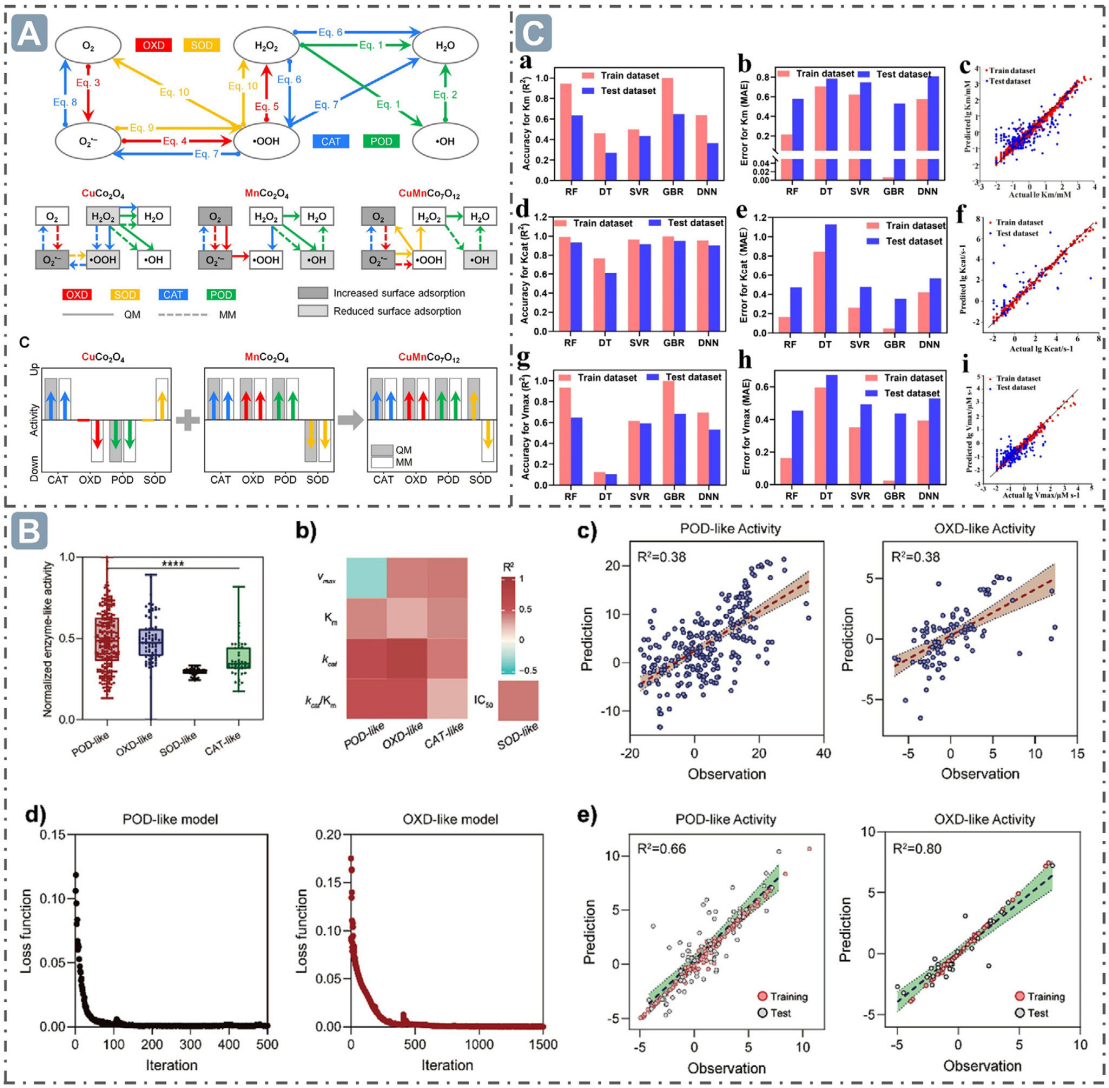
ML model for the prediction of nanozyme types. A) ML model for the prediction of multienzyme‐like activities. Reproduced with permission.^[^ ^179^ ^]^ *Copyright 2024 American Chemical Society*. B) Classification model for the prediction of nanozyme catalytic types. Reproduced with permission.^[^ ^20^ ^]^ *2022 Wiley‐VCH GmbH*. C) Adaboost‐DT model for the prediction of OXD‐like, POD‐like, CAT‐like, and SOD‐like activities. Reproduced with permission.^[^ ^185^ ^]^ *Copyright 2024 American Chemical Society*.

Classification ML models have also been widely applied in predicting nanozyme types, effectively enabling the design of nanozymes to advance from a “trial and error” approach to “precise customization”. For example, nanozyme‐related information extracted from 4159 papers, containing element type, proportion, chemical valence state, morphology, pH, and other parameters, was used to construct a structured database. Following cluster analysis, the key material characteristics of different nanozymes were determined, which further facilitated the determination of composition factors for multienzyme‐like nanozymes. Subsequently, quantum mechanics/molecular mechanics methods were employed to unravel the catalytic mechanisms through analyzing the *E_ads_* and binding energy of substrate, transition state, and products during the reaction process. These insights were then utilized to assist the development of multienzyme‐like nanozymes. Rapid‐exploring random trees (RRT) model and Dijkstra's algorithm were utilized to explore the possible catalytic pathways, whether the binding energy was considered or not, respectively. Ultimately, CuMnCo_7_O_12_ nanozyme, with multiple enzyme‐mimicking activities, was successfully developed through the integration of the developed ML model, whose design concept is analogous to genetic evolution (**Figure** **17**A).^[^ ^179^ ^]^ Additionally, 920 data entries were selected from numerous documents to build a nanozyme database. Factors influencing nanozymes were categorized into internal and external factors, and the data were digitized. Based on this, deep neural network (DNN) models were developed for both classification (predicting enzyme simulation types) and quantification (predicting nanozyme activity levels). The classification model achieved a prediction accuracy of 90.6%. Through SHAP analysis, it was found that internal factors related to transition metals and external factors, such as substrate and buffer pH, exerted significant impacts on the enzyme‐mimicking types. Among the quantitative models, POD‐like and OXD‐like models exhibited favorable predictive performance, with R^2^ values reaching 0.66 and 0.80, respectively. In contrast, SOD‐like and CAT‐like models showed limited effectiveness due to the data limitations. Experimental validation confirmed that the model's predictions of enzyme‐mimicking types and activity levels for various nanozymes were highly consistent with the experimental results. Furthermore, leveraging the model, researchers also explored the relationship between transition metals from different periods and nanozyme performance, successfully designing and validating Ru‐based nanomaterials with high POD‐like activity and Mn‐based nanomaterials with high OXD‐like activity (Figure 17B).^[^ ^20^ ^]^ Besides, a nanozyme database, containing chemical formula, metal type, metal ratio, metal valent, nonmetal doping, surface modification, size, nonmetal doping, synthesis pathways, temperature, buffer pH, dispersion medium, substrate type, substrate concentrations, nanozyme types, *K*_m_, *V*_max_, *K*_cat_, and *IC*_50_, was built to predict the nanozyme types through DT, RF, adaboost‐RF, and adaboost‐DT ML model. After optimizing the hyperparameters of the adaboost‐DT model using BayesSearchCV, the adaboost‐DT model achieved excellent performance in predicting nanozyme types in terms of accuracy, F1 score, recall, and precision, which yielded the prediction accuracy of 78%, 85%, 75%, and 55% for OXD‐like, POD‐like, CAT‐like, and SOD‐like nanozyme types, respectively (Figure 17C).^[^ ^185^ ^]^ Through multi‐dimensional data integration and algorithm innovation, breakthroughs have been achieved in the entire chain of nanozyme type prediction, catalytic mechanism analysis, and performance optimization.

### 5.4 Prediction of Nanozyme Other Properties

**Figure 18:**
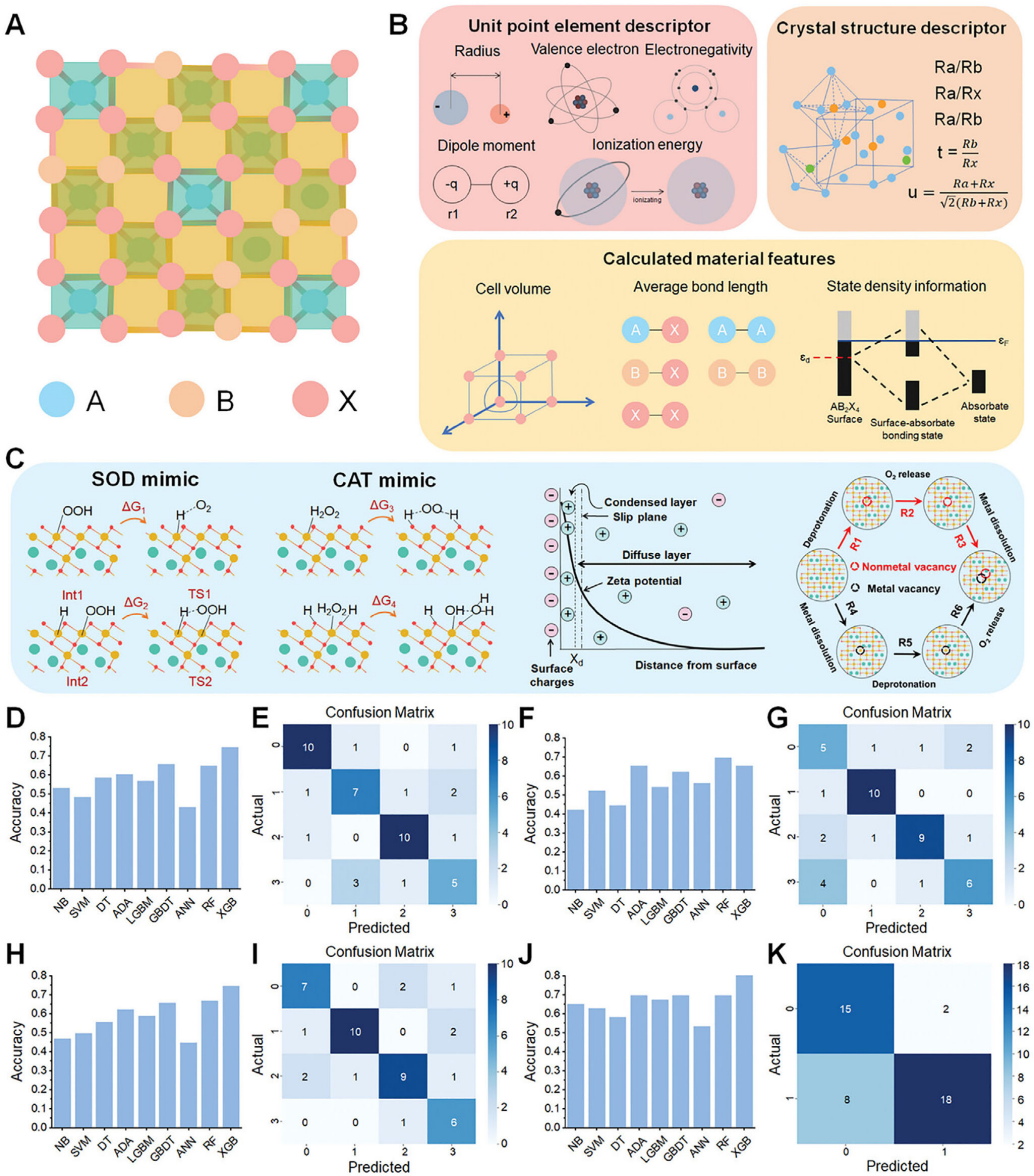
The translation of the application requirements into actionable material descriptors for boosting nanozyme feasibility. Reproduced with permission.^[^ ^181^ ^]^ *2025 Wiley‐VCH GmbH*.

In practical applications, nanozymes must also take other properties into consideration. ML technology boasts a powerful ability to convert real requirements into operable material descriptors, providing a universal paradigm for the design of nanozyme in real‐world applications. For example, AB_2_X_4_ nanozymes with SOD‐like and CAT‐like activities are promising candidates for ulcerative colitis treatment, owing to their distinctive crystal structures and nanozyme activities. To meet the multifunctional requirements of ulcerative colitis treatment, antioxidant activity, acid stability, and targeting ability were translated into corresponding material simulation indexes for subsequent ML model testing, such as SOD‐CAT reaction barrier, lattice anion stability, and zeta potential. First, 4104 AB_2_X_4_ crystal structures were generated by combining known atomic types. Subsequently, a comprehensive material characterization database was constructed, integrating open‐access data and high‐throughput computations. This database included over 30 descriptors, such as atomic radius, electronegativity, and tolerance factor, enabling systematic analysis of site‐specific properties and macroscopic material behaviors. SOD‐like and CAT‐like activities were characterized by the maximum energy barrier of the two‐step transition state reaction energy (△G_1_ and △G_2_) and the maximum energy barrier of △G_3_ and △G_4_, respectively. Zeta potential and lattice anion dissolution reaction energy were used to describe the mucosal adhesion and drug retention capacity, and the acid stability, respectively. By using 213 stable AB_2_X_4_ compounds from the Materials Project as a training set, feature redundancy was eliminated using Pearson correlation coefficient screening (threshold <0.3). After hyperparameter optimization through grid search, Native Bayes (NB), SVM, DT, ADA, LGBM, GBDT, CNN, RF, and XGBoost models were used to evaluate the database. Among these, the XGBoost model showed the optimal performance in terms of accuracy, F1 score, and confusion matrices. SrDy_2_O_4_ was identified as the optimal nanozyme candidate, showing balanced SOD‐like/CAT activities (ΔG <0.8 eV), robust acid stability (dissolution energy >1.2 eV), and a strong negative zeta potential (‐28 mV). In vitro simulations validated its enhanced mucus penetration and drug retention in inflamed intestinal environments, offering a generalizable paradigm that integrates computational screening, experimental validation, and clinical relevance in nanomedicine development (**Figure** **18**).^[^ ^181^ ^]^ In conclusion, ML‐driven multi‐scale modeling in translating the application requirements into actionable material descriptors, enabling rational design of high‐performance nanozymes for complex applications. In conclusion, ML‐driven multi‐scale modeling has succeeded in translating application requirements into actionable material descriptors, enabling the rational design of high‐performance nanozymes for complex applications.

### 5.5 Interpretable Analysis and Mechanism Revelation

**Figure 19:**
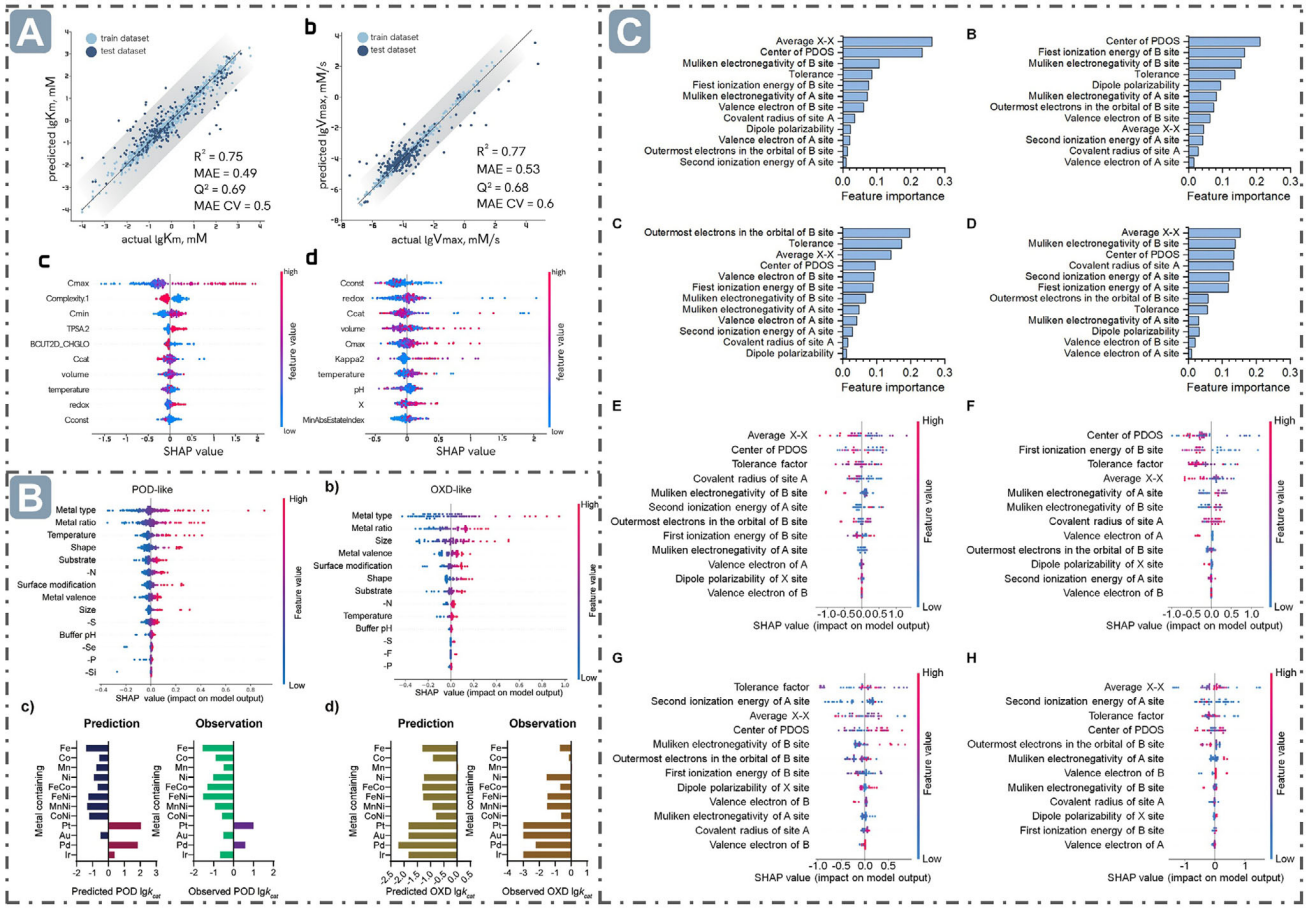
The interpretation of ML models for boosting nanozyme catalysis. A) The ML model prediction and feature importance explanation. Reproduced with permission.^[^ ^186^ ^]^ *Copyright 2024 American Chemical Society*. B) The model explanation for the features of metal‐based nanozymes through importance quantification. Reproduced with permission.^[^ ^20^ ^]^ *2022 Wiley‐VCH GmbH*. C) Black‐box explanation for four optimal ML models of nanozyme application features. Reproduced with permission.^[^ ^181^ ^]^ *2025 Wiley‐VCH GmbH*.

To further understand the structure‐activity relationship of nanozymes, interpreting the relationship between ML prediction results and nanozyme features has become a significant subject for revealing nanozymes’ catalytic mechanisms. For example, SHAP was employed to analyze the average feature influence (AFI) of nanozyme, thereby clarifying the mechanism underlying ML models for the prediction of nanozyme catalytic activities. SHAP analysis revealed that the critical intrinsic determinants of nanozyme performance are redox potential (SHAP AFI = 0.17) and electronegativity (AFI = 0.10): lower electronegativity can enhance catalytic efficiency by reducing electron transfer resistance, while redox potential positively correlates with the material's tendency to participate in redox reactions. Among molecular substrate parameters, complexity (AFI = 0.25) and topological polar surface area (TPSA, AFI = 0.10) emerged as dominant factors: substrates with high complexity and TPSA (e.g., TMB, ABTS) exhibit stronger nanozyme affinity but lower reaction rates. Additionally, coating topological descriptors Kappa2 (AFI = 0.11) and BlalabanJ (AFI = 0.09) significantly regulate catalytic kinetics through interfacial interactions. This is exemplified by CTAB‐, BSA‐, and PVP‐coated nanozymes, where high Kappa2 values correlate with reduced *K*_m_/*V*_max_. These findings not only unravel the microscopic mechanisms underlying nanozyme‐substrate interactions but also provide quantitative evidence for rational interface engineering, highlighting the unique capability of ML models in deciphering complex structure‐activity relationships (**Figure** **19**A).^[^ ^186^ ^]^ Besides, SHAP analysis revealed that metal‐based factors, particularly transition metal types, exerted dominant influences on POD/OXD‐like activity levels, which were consistent with the ML prediction results. To validate the rationality of SHAP analysis and the model's robustness, ferritin nanocages‐based (FTn‐based) nanozymes incorporating distinct metals (e.g., Ru, Pd, Ag) were synthesized, and their observed activities showed high consistency with the predicted results. Subsequently, significant correlations were identified between transition metal periods and enzyme‐like activities: period V metals (e.g., Ru) demonstrated optimal POD‐like performance (R^2^ = 0.89), while period IV metals (e.g., Mn) excelled in OXD‐like activity (R^2^ = 0.85). By fixing parameters, such as pH and size, Ru NPs with the highest POD‐like activity (*K*_cat_ = 6.25 s^−1^) was successfully predicted by the ML model, which was experimentally validated by Michaelis‐Menten kinetics curves of Ru/Pd/Ag NPs. Additionally, OXD‐like activities predicted by the ML model were also demonstrated by experiments. Collectively, explaining the features underlying the model's prediction results can reveal the catalytic mechanisms of nanozymes and pave a new way for designing high‐performance nanozymes (Figure 19B).^[^ ^20^ ^]^ Additionally, to reveal the structure‐activity relationships between nanozymes and ulcerative colitis treatment features, the average influence of features, such as average X‐X, PDOS center, and Muliken electronegativity at B site, on the prediction models for SOD‐like barrier, CAT‐like barrier, acid stability, and zeta potential prediction model was analyzed. Notably, the average X─X bond length and PDOS center showed the strongest correlation with SOD‐like activity, while other features also played an important role in regulating their corresponding properties. Furthermore, the SHAP method was used to account for interactions between features, revealing that the PDOS center, average X─X bond length, and Mulliken electronegativity and tolerance factor at the B site were key features in all properties. These features were closely related to the nanozyme characteristics required for ulcerative colitis treatment. Additionally, the influence ranges of these critical features were analyzed by a correlation diagram, and the optimal ranges for the electronegativity and tolerance factor of Muliken at PDOS center, average X‐X, and B sites were determined. Within these ranges, nanozymes were more likely to exhibit properties related to ulcerative colitis treatment (Figure 19C).^[^ ^181^ ^]^ In summary, the interpretation of ML models can successfully elucidate the structure‐activity relationship of nanozymes, providing a methodological breakthrough for the rational design of nanozymes.

## 6 Challenges and Perspectives

**Figure 20:**
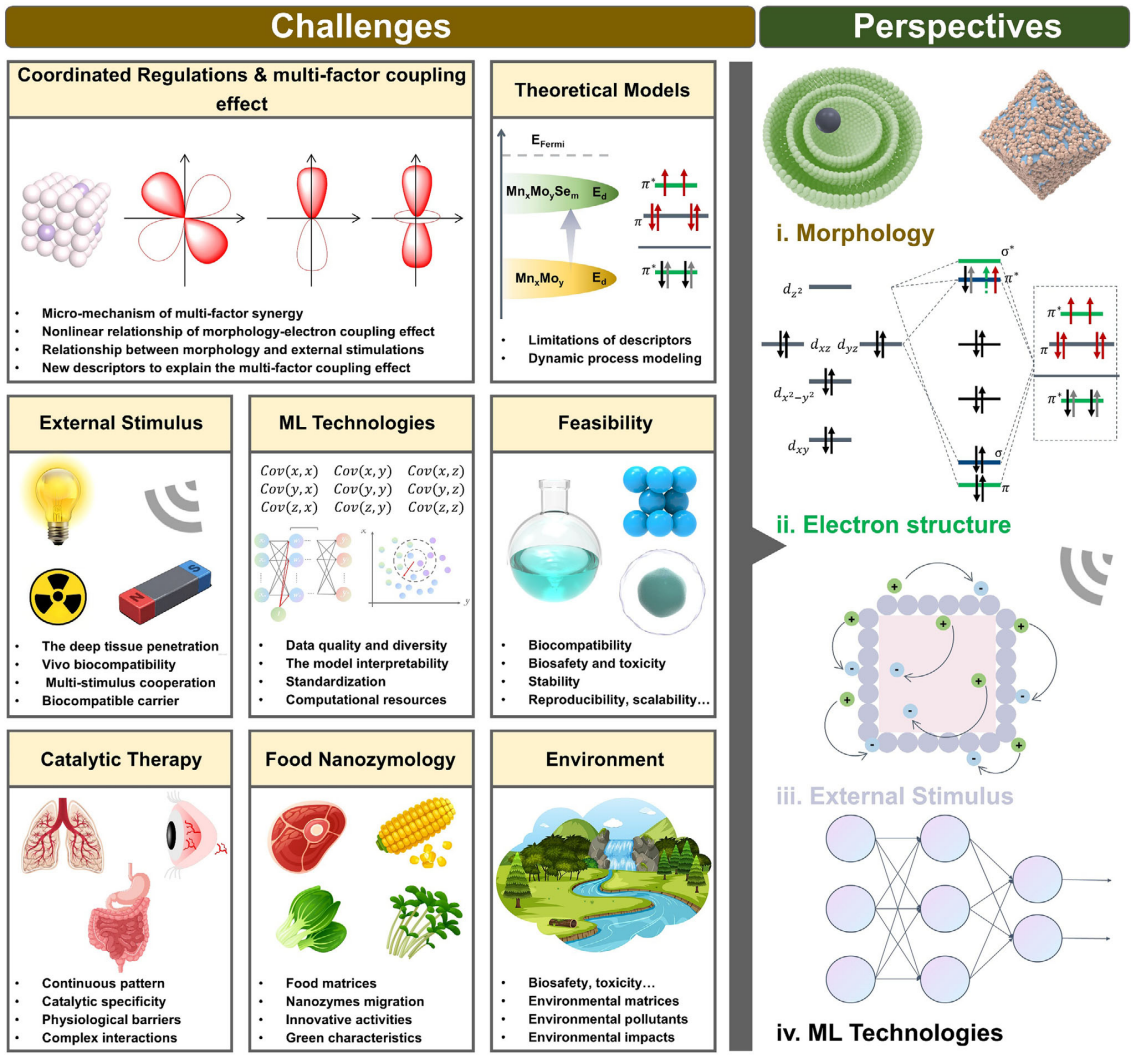
Challenges and perspectives of multi‐dimensional synergistic engineering for boosting nanozyme catalysis.

Although transdisciplinary breakthroughs have been made to advance nanozyme catalysis, persistent challenges still hinder the development of high‐activity nanozymes for practical applications (**Figure** **20**). Herein, we propose the key challenges and prospects as follows:

1. The dilemma of coordinated regulation. Although regulatory strategies targeting morphological structures, such as size, morphology, hollow, and pore, and electronic structures, containing *d*‐band center, *e_g_* occupation, defects, etc., have significantly enhanced nanozyme activity, the micro‐mechanism underlying multi‐factor synergy remains unclear. For example, the nonlinear relationships in morphology‐electron coupling effects have yet to be formalized into a universal theoretical model, restricting the rational design of high‐performance nanozymes. Specifically, nanozyme morphologies, such as NSs and NRs, and defect structures, can alter the coordination environment of surface atoms, thereby further influencing the electronic structure, such as the *d*‐band center and the *e_g_* occupancy. Another case in point: external stimuli like light and electricity can not only promote nanozyme catalysis but also have the potential to alter nanozyme morphology. Another example, external stimulations, such as light and electricity, can not only promote nanozyme catalysis, but also have the potential to alter nanozyme morphology. Future research can focus on modular design, such as surface modification, doping strategies, and support engineering, to independently regulate nanozyme catalytic activity. For example, decoupling morphological regulation from electronic structure regulation can reduce the complexity of multi‐factor synergistic optimization.
2. Ambiguity in the micro‐mechanisms of multi‐factor coupling effects. The active sites of nanozymes often exhibit uneven distributions of chemical composition and electronic distribution. For example, in Janus‐structured nanozymes, the electronic distribution of the nanozyme is challenging to accurately characterize using traditional methods. Furthermore, for nanozymes with multiple catalytic sites, such as HEzymes containing diverse active sites, the random distribution of various metal atoms gives rise to complex synergistic effects among the electronic structures of active sites, which cannot be fully elucidated by a single descriptor. Future research can focus on ML techniques, such as dimensionality reduction algorithms, to extract feature values and develop new descriptors for elucidating multi‐factor coupling effects.
3. Lack of universality in theoretical models. i) Limitations of descriptors. Currently, widely used descriptors, such as *d*‐band center and *e_g_* occupancy, are only applicable to specific nanozymes, making it difficult to universally account for the catalytic behavior of complex nanozymes. For example, the *d*‐band center theory excels in explaining the adsorption behavior of noble metal nanozymes, such as Pt‐based nanozymes, the capacity for H_2_O_2_ adsorption, and O─O bond cleavage can be optimized by tuning the *d*‐band position relative to the Fermi level, yet its explanatory power for other nanozymes is markedly diminished. Similarly, the *e_g_* occupancy theory, which establishes a volcanic curve relationship with a peak value eg≈1.2 in perovskite oxides, has not been widely validated across other nanozyme types. ii) The challenge of modeling dynamic processes is even more complicated. Nanozyme catalysis involves multi‐step reactions, dynamic adsorption/desorption of intermediates, and responses to external stimuli, features that traditional steady‐state models struggle to capture dynamically. Moreover, electron transfer pathways under external stimuli, such as electric fields and US, are directional and time‐dependent, rendering them challenging to describe with traditional models. Future efforts should focus on developing dynamic descriptor systems and integrating more advanced characterization techniques to track catalytic processes in real time. This includes building multi‐scale coupling models to unravel the micro‐mechanisms of dynamic processes, leveraging ML to integrate experimental data and theoretical calculations for constructing dynamic response network models, and employing innovative in situ characterization techniques to observe the real‐time structural evolution of nanozymes. Overcoming these bottlenecks is expected to enable the establishment of a more universal theoretical framework for nanozyme catalysis, providing a scientific foundation for the rational design of high‐performance nanozymes.
4. Engineering challenges in external stimulus responsiveness. Nanozymes face multiple engineering hurdles in practical applications when responding to external stimuli, such as US, light, electricity, primarily manifested in energy conversion efficiency, in vivo stability, spatiotemporal precise regulation, and large‐scale fabrication. For example, deep tissue penetration of photothermal nanozymes is constrained by light absorption efficiency and wavelength selection, particularly in solid tumors. Additionally, energy attenuation in US and electrical stimulation is significant, leading to diminished therapeutic efficacy for deep‐seated tumors. Moreover, in vivo stability and biocompatibility pose another core challenge: nanozymes are susceptible to enzymatic degradation, pH fluctuations, or protein adsorption in physiological environments, while the biological toxicity of stimulus‐responsive materials cannot be overlooked. Furthermore, the complexity of multi‐stimulus collaborative systems exacerbates engineering difficulties. Future research should focus on developing high‐efficiency energy conversion materials, such as NIR‐II‐responsive photothermal materials and high‐frequency US piezoelectric NPs. Constructing biocompatible carrier systems can enhance in vivo stability. Innovations in spatiotemporal precise control technologies, such as integrating miniaturized photoacoustic imaging with magnetic field‐guided ultrasonic focusing, will enable real‐time monitoring and accurate positioning of treatment regions. For multi‐stimulus collaborative systems, optimizing energy complementary mechanisms is critical, as exemplified by photo‐magnetic synergistic nanozymes that utilize magnetic fields to guide photothermal therapy. Through material and device innovations, the translation of stimulus‐responsive nanozymes from laboratory to clinical applications can be promoted. Thus, future efforts should be dedicated to developing high‐efficiency energy‐converting nanozymes, constructing biocompatible carrier systems, and optimizing energy complementary mechanisms.
5. Application bottleneck of ML technologies. Although the application of ML technologies in nanozyme design shows revolutionary potential, its development is still restricted by multiple bottlenecks: i). The lack of data quality and diversity is the core challenge. The existing databases often concentrate on the traditional materials database, such as Fe_3_O_4_ and CeO_2_ nanozymes, but lack coverage of new materials, such as HEzymes and support‐based nanozymes, resulting in the limited generalization ability of the ML model. Besides, the multi‐resources data from experiment and calculation is difficult to integrate, and the format differences need to be manually aligned, which easily introduces deviations. ii). The lack of model interpretability hinders the mechanism analysis. Although black‐box models, such as ANN exhibit high accuracy in nanozyme prediction, they fail to reveal the physicochemical essence underlying feature importance. For example, the relationship between “transition metal period” can be revealed by SHAP analysis, but the internal relationship with *d*‐band center is failed to predicted, thereby increasing the cost of experimental validation. iii). Standardization and repeatability cannot be ignored. The catalytic activity of the same nanozyme reported by different laboratories can vary significantly, potentially due to inconsistent experimental conditions, which further impairs the accuracy of nanozyme activity predictions. vi). Computational resources are as significant as efficiency. The high‐dimensional feature space of nanozymes can lead to dimension disaster. The high cost of theoretical calculation, such as DFT, further limited the data scale. The lack of high‐throughput calculation technology can prolong the screening cycle for new materials. Future research should focus on the following: constructing a multimodal collaborative database that integrates experimental, computational, and application data, developing interpretable enhanced models that combine physicochemical mechanisms with ML, optimizing high‐throughput computational workflows and accelerating data acquisition; establishing dynamic environmental response models to capture real‐time parameter changes, and promoting standardization and open‐source collaboration. By overcoming these bottlenecks, ML can truly empower the precise design of nanozymes and advance their translation from laboratory research to practical applications.
6. Biocompatibility, biosafety, and toxicity. Although nanozymes have been frequently and enthusiastically recognized as potential to address “global challenges”, some critical translational hurdles from laboratory to industry still exist. Although the initial cytotoxicity screening of nanozymes in medicine has often evaluated and demonstrated their biological safety, the truly comprehensive biocompatibility assessment requires more in‐depth research. First, the long‐term biodistribution and fate of nanozymes in in vivo should be considered and make sense of the long‐term consequences of accumulation. Besides, nanozymes may be as immunogens, causing immune responses in human beings and triggering hypersensitivity to damage the organisms. Moreover, the toxic effects of the degradation products and metabolites of nanozymes in physiological environments should be further studied. Therefore, a comprehensive therapeutic evaluation index should be considered to construct the balance between therapeutic efficacy and toxicity, which requires extensive in vivo toxicology studies across multiple models and doses.
7. Stability under physiological conditions, reproducibility, and scalability of synthesis. Nanozymes may exhibit reduced catalytic activity or structural degradation in complex biological environments, which may be attributed to non‐specific adsorption, ionic strength, pH variations, aggregation, and etc. The instability under physiological conditions can lead to unpredictable catalytic behavior, and thus reduce the therapeutic efficacy or diagnostic performance. Therefore, the long‐term stability of nanozymes under physiological conditions is essential for their sustained function. To achieve this goal, surface engineering can be considered to modify nanozymes for enhancing their stability, such as using biocompatible polymer coatings or biomimetic membranes. Besides, the nanozyme compositions can be carefully selected for the specific physiological condition to enhance their stability. Besides, some subtle variations in synthesis parameters, such as precursor concentration, temperature, reaction time, pH, and etc., can significantly alter the nanozyme performance, such as size, morphology, crystal facets, defect density, and ultimately catalytic activity, which greatly hinders reliable comparison from development to application. Therefore, the strict protocol standardization, detailed reporting of all synthesis and purification steps, advanced process control, and comprehensive characterization techniques should be conducted to standardize the nanozyme synthesis to improve the reproducibility of nanozyme synthesis. Besides, many promising nanozymes synthesis are dependent on small lab‐scale batches and are unable to achieve industrial‐scale production. Therefore, future works must actively explore and develop the industrial‐scale production of nanozymes to bridge the gap between lab‐scale discovery and industrial‐scale production.
8. In the field of nanozyme catalytic therapy. Nanozyme, benefiting from the enzyme‐mimicking activity, is one of the important catalytic materials of pancatalytic therapy.^[^ ^187^, ^188^ ^]^ Although nanozymes have shown great potential in catalytic therapy, they still face many limitations and challenges in basic research and clinical translation. First, the catalytic reactions of nanozymes mostly follow a continuous pattern, making it difficult to precisely control the start/stop timing. For example, nanozyme with POD‐like activity can catalyze the production of excessive ROS in advance before reaching the lesion, resulting in oxidative damage to normal tissues. Therefore, how to realize the “on‐demand activation” and “precise shutdown” of nanozyme catalysis can be responsible for the improvement efficiency of catalytic therapy. Future research can improve the catalytic precision of nanozyme therapy through external stimulations, such as light and US, and endogenous signals, such as lesion‐specific molecules. Additionally, after entering the body, nanozymes can be easily cleared by the reticuloendothelial system, resulting in an extremely low proportion reaching the lesion sites. Therefore, the specificity of nanozyme catalytic therapy should be enhanced, which can be achieved by surface modification with targeting ligands, such as antibodies and peptides. Besides, for central nervous system diseases, such as Alzheimer's disease or deep tissue lesions, nanozymes have difficulty penetrating physiological barriers, such as the blood‐brain barrier and the vascular endothelial barrier. More importantly, the interaction between nanozymes and biological systems involves complex multi‐scale and multi‐target processes. For example, they not only act directly through catalytic reactions, but may also affect cellular signaling pathways through non‐catalytic pathways, such as binding to cell membrane receptors. Meanwhile, the abovementioned biocompatibility, biosafety, toxicity, stability, reproducibility, and scalability of nanozymes should also be considered.
9. The extension of food nanozymology. With Huang's group clearly and systematically defining the concept of food nanozymology, nanozymes in the application of food science have entered a new era of rapid development,^[^ ^189^ ^]^ which also puts forward new requirements for food nanozymology. Nanozymes provide revolutionary new tools and ideas for solving many problems in food science, especially rapid detection and intelligent preservation. Food matrices are usually very complex, containing a large number of proteins, fats, carbohydrates, pigments, minerals, and other small molecules, and these components may compete for catalytic sites, alter the surface properties of nanozymes, or inhibit their activity. Thus, the efficiency of nanozyme detection and processing methods can be decreased. Moreover, the color, turbidity, or fluorescent background of the food matrix itself may generate interfering signals, affecting the accuracy and reliability of the detection results. Besides, it is necessary to ensure extremely low or even zero migration of nanozymes into food through material design and processing technology when used for food contact materials (packaging). Therefore, it is necessary to clarify the residue limit and be highly sensitive detected when directly added to food or used as a processing aid. Additionally, future innovation should not be limited to optimizing the common activities of existing POD‐like, OXD‐like, etc., but should boldly explore new catalytic capabilities to address the pain points and demands of the food industry. For example, the development of nanozymes with strong cellulase‐like, hemicellulose‐like, pectinase‐like, protease‐like, or lipase‐like activity can greatly promote the utilization of food processing, such as more mild and efficient juice clarification, dairy product modification, oil refining, and meat tenderization. Moreover, the synthesis of nanozymes with both high activity and green characteristics can be considered by using sustainable raw materials. Therefore, the development of these innovative active nanozymes will push the application of nanozyme technology in food science to a deeper and broader dimension, and solve the insurmountable challenges of traditional technology.
10. In the field of environment. Although nanozymes hold immense promise for revolutionizing environmental monitoring and remediation, their practical deployment faces significant hurdles beyond the abovementioned universally applicable challenges of biocompatibility, biosafety, toxicity, stability, reproducibility, and scalability. The complex environmental matrices, such as soil, air, and water, contain high concentrations of dissolved organic matter, inorganic ions, and suspended particles, which have a great interference in nanozyme catalytic activities through adsorbing onto nanozyme surfaces, blocking active sites, even reacting with intermediates, etc., significantly reducing detection sensitivity and degradation efficiency. Additionally, the diverse environmental pollutants, such as persistent organic pollutants, heavy metals, emerging contaminants, and antibiotic residues, require some nanozymes with abundant catalytic activities to enhance the specificity of nanozymes towards pollutants, especially in a mixed pollutants environment. Crucially, the potential environmental impact of nanozymes demands equal attention. Any large‐scale application inevitably leads to its release into ecosystems, posing complex risks to environmental and human health. For example, nanozymes exposed to the environment may be bioaccumulated in animals and plants and bring the long‐term and low‐dose effects to ecosystem, which even convey this effect to human beings through the food chain. Therefore, rigorous assessment of nanozyme environmental impact is not merely an afterthought—it is a fundamental prerequisite for responsible innovation. Prioritizing this research will be instrumental in bridging the gap between promising laboratory results and viable, sustainable industrial applications.

## 7 Conclusion

In conclusion, the cutting‐edge strategies in boosting nanozyme from four synergistic dimensions were systematically examined, which developed a theoretical framework integrating morphological structure engineering, electronic structure optimization, external stimulation responses, and ML‐assisted design. Initially, the structure‐activity relationship between nanostructures and catalytic performance was comprehensively discussed, revealing the regulation mechanism of active site exposure, substrate accessibility, and electron transfer kinetics. After comprehensively discussing the electronic structure of nanozymes, the mechanism of electronic structure‐governed catalytic activities through all kinds of pathways was deeply summarized. Focusing on external stimulations, the recent progress and mechanism of dynamic regulation through external stimulations to boost nanozyme catalysis were analyzed in detail. Notably, we have also highlighted the transformative potential of ML‐accelerated high‐throughput screening in deciphering multidimensional structure‐activity correlations and streamlining the development of optimized high‐performance nanozymes. These strategies not only elucidate fundamental mechanisms underlying enhanced nanozyme catalysis but also establish actionable frameworks for designing application‐tailored nanozymes with programmable functionalities. Finally, we have proposed that interdisciplinary convergence spanning materials innovation, external regulations, and artificial intelligence will be pivotal in overcoming current limitations, ultimately unleashing the full potential of nanozymes to address pressing global challenges, while establishing nanozymology as an autonomous discipline through paradigm‐shifting methodologies. It is anticipated that innovative, high‐performance nanozymes will bring greater well‐being to human health, particularly in the fields of environmental monitoring and control.

## Conflict of Interest

The authors declare no conflict of interest.
